# Transcription Factor Networks derived from Breast Cancer Stem Cells control the immune response in the Basal subtype

**DOI:** 10.1038/s41598-017-02761-6

**Published:** 2017-06-06

**Authors:** W. A. da Silveira, P. V. B. Palma, R. D. Sicchieri, R. A. R. Villacis, L. R. M. Mandarano, T. M. G. Oliveira, H. M. R. Antonio, J. M. Andrade, V. F. Muglia, S. R. Rogatto, C. Theillet, S. du Manoir, D. G. Tiezzi

**Affiliations:** 1 Ribeirão Preto Medical School, University of São Paulo, Ribeirão Preto, Brazil; 2 INSERM U1194, 34298 Montpellier cedex 5, Montpellier, France; 3 National Institute of Science and Technology in Stem Cell and Cell Therapy, Center for Cell Therapy and Regional Blood Center, Ribeirao Preto, Brazil; 4 Department of Clinical Genetics, Vejle Sygehus, Vejle, and Institute of Regional Health, University of Southern, Denmark, Denmark; 5 Department of Genetics and Morphology, Institute of Biological Sciences, University of Brasília - UnB, Brasília, DF Brazil; 6 Department of Urology, Faculty of Medicine, UNESP, Sao Paulo State University, Botucatu, Sao Paulo Brazil; 7 Institut de Recherche en Cancérologie de Montpellier, Université de Montpellier, 34298 Montpellier cedex 5, Montpellier, France; 8 Institut de Cancérologie de Montpellier (ICM), 34298 Montpellier cedex 5, Montpellier, France; 9 CISBi – Center for Integrative Systems Biology – Ribeirão Preto Medical School, University of São Paulo, Ribeirão Preto, Brazil

## Abstract

Breast cancer is the most common cancer in women worldwide and metastatic dissemination is the principal factor related to death by this disease. Breast cancer stem cells (bCSC) are thought to be responsible for metastasis and chemoresistance. In this study, based on whole transcriptome analysis from putative bCSC and reverse engineering of transcription control networks, we identified two networks associated with this phenotype. One controlled by *SNAI2*, *TWIST1*, *BNC2*, *PRRX1* and *TBX5* drives a mesenchymal or CSC-like phenotype. The second network is controlled by the *SCML4*, *ZNF831*, *SP140* and *IKZF3* transcription factors which correspond to immune response modulators. Immune response network expression is correlated with pathological response to chemotherapy, and in the Basal subtype is related to better recurrence-free survival. In patient-derived xenografts, the expression of these networks in patient tumours is predictive of engraftment success. Our findings point out a potential molecular mechanism underlying the balance between immune surveillance and EMT activation in breast cancer. This molecular mechanism may be useful to the development of new target therapies.

## Introduction

Breast cancer is the most common cancer in women worldwide and metastatic dissemination is the leading cause of death by this disease^1, 2^. Breast cancer can be subdivided into molecular entities on the basis of expression profiling data^3, 4^.

A classification defining 5 subtypes (Normal-like, Luminal A, Luminal B, ERBB2, Basal subtypes) has reached consensus. Of these the Basal subtype represents ~15% of invasive breast cancers, and is considered to be the most aggressive and bearing the poorest outcome^4^.

The cancer stem cell (CSC) hypothesis proposes the existence of a subpopulation of cells that possess the ability to self-renew and to generate the heterogeneous lineages of cancer cells that comprise the original tumour^5, 6^. Accordingly, these cells, positioned at the apex of cellular hierarchy in the tumour, bear increased resistance to chemotherapy^5, 6^. The mechanisms responsible for the origin and maintenance of the stem cell phenotype are actively studied and not completely elucidated yet^5, 7–9^. The definition of the breast cancer stem cell (bCSC) is still in debate. The identification and/or isolation rely on several breast CSC biomarkers and methodologies, including flow cytometry, *in vivo* tumorigenic models, mammospheres and histological immunostaining^10^. Among a number of proposed biomarkers, elevated activity of aldehyde dehydrogenase 1 (ALDH1) has raised tremendous interest due to the increased sphere forming capacity and tumorigenicity of the ALDH positive cell fraction^10^. Interestingly, similar findings, including tumour initiating capability, were made in pancreatic adenocarcinoma, multiple myeloma and glioblastoma suggesting the subset of ALDH^high^ cells population is enriched for CSCs^5, 6, 11–14^.

Transcription Factors (TFs) are the principal regulators of gene expression in eukaryotic cells^15, 16^. Combinations of TFs act together to build cellular states, regulating their target genes to mobilize the appropriate protein response according to specific signalling^16, 17^. Close to 6% of the protein-coding genes in the human genome are estimated to code for TFs^16^. A network organized around the TF families of *SNAIL*, *ZEB* and TWIST, is well known to be responsible for the epithelial-to-mesenchymal transition^18^(EMT), a process involved in metastasis and closely related to the bCSC phenotype^2, 5^.

Expression of genes defining a phenotype is highly coordinated. Using reverse engineering algorithms of large set of transcriptomes, it is possible to interpret large transcriptional networks in terms of regulons, master TFs regulators and gene circuits as exemplified in the seminal work of Carro and coworkers^15^.

We decided to apply this strategy with the aim to identify TFs acting as master regulators of the bCSC phenotype and evaluate their impact on patient clinical outcome. To achieve this goal, we opted for the analysis of transcriptomes generated from breast cancer cell subsets enriched in CSCs isolated by Fluorescence Activated Cell Sorting (FACS).

Using this strategy, in the context of breast cancer, we identified new TFs as master regulators of the bCSC phenotype and demonstrate their influences on the clinical outcome of patients with the Basal phenotype.

## Results

### Provisional identification of Transcription factors of the breast cancer stem cell phenotype and Inference of its modules

In order to identify TFs that may be master regulators (MRs) of breast cancer stem cells, we assessed the expression of TFs in bCSC-enriched samples sorted by FACS based on ALDH1 activity. We carried out a consensus analysis of our three paired samples of bCSC and total tissue, bCSC/Bulk dataset, and 8 paired samples of bCSC and cancer cells, bCSC/CC dataset. We found 17 TFs differentially expressed in the bCSCs in both comparisons. Most of them (13) were up-regulated (*PRRX1*, *SNAI2*, *TWIST1*, *ID4*, *BNC2*, *GATA6*, *ZNF503*, *FOXF2*, *HOXA5*, *HOXB3*, *TSC22D1*, *TBX5* and *CREB3L1*) and four down-regulated (*SCML4*, *ZNF831*, *IKZF3* and *SP140*). These TFs present a consistent pattern of expression in the two datasets (Pearson correlation coefficient, r = 0.84; p < 0.001; Supplementary Figure S1).

**Figure 1:**
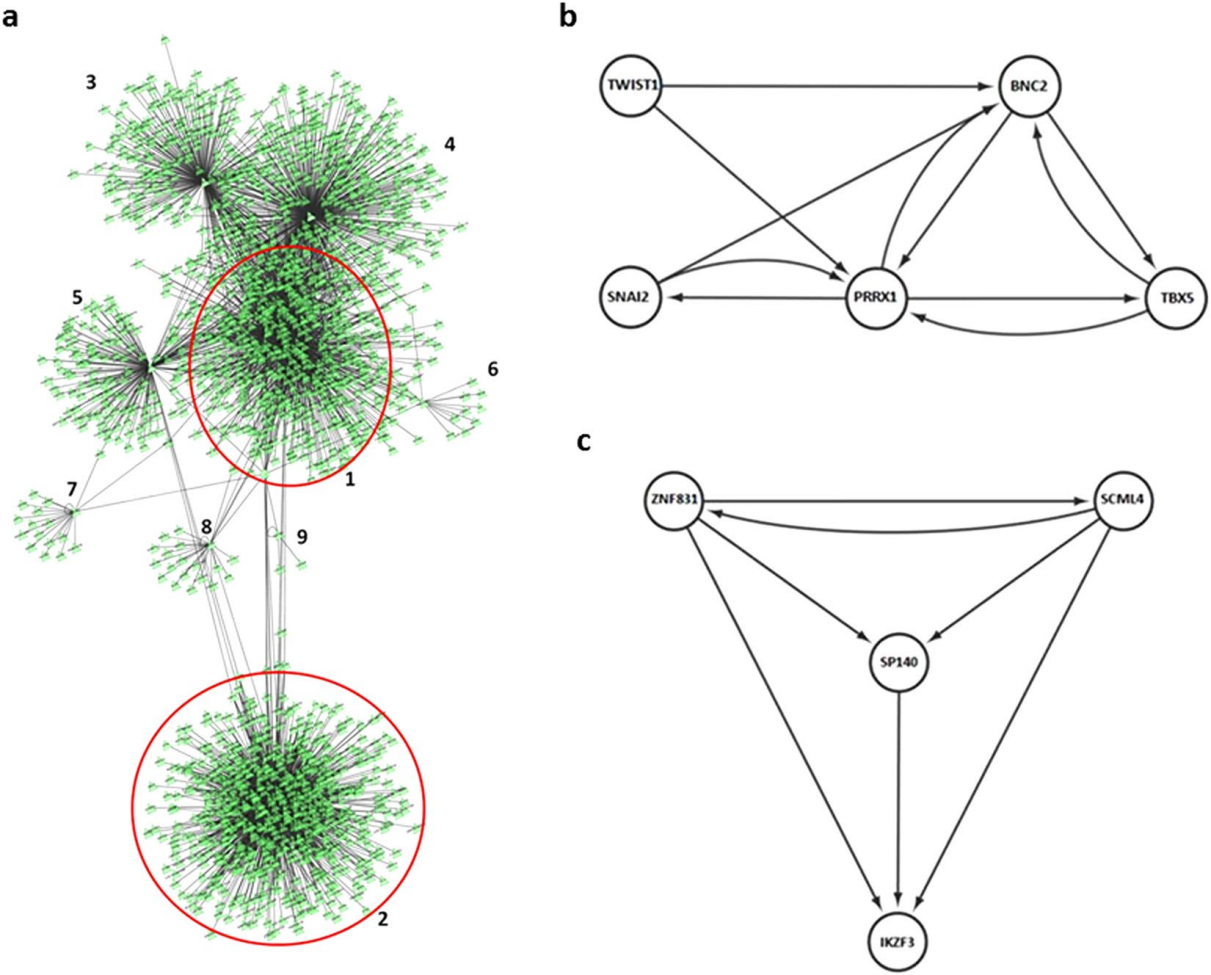
Transcription factor modules. (**a**) - Graphical representation of the regulons of the 17 Transcription Factors in the NACT dataset Inferred by ARACNE algorithm. Regulons: 1 – SNAI2, TWIST1, PRRX1, BNC2, TBX5, 2 – SCML4, ZNF831, SP140, IKZF3, 3 – ID4, 4 – HOXA5, 5 – ZNF503, 6 – TSC22D1. 7 – HOXB3, 8 – CREB3L1, 9 – GATA6. The Regulon of FOXF2 has 32 genes, these genes are shared by the regulons of SNAI2, TWIST1, PRRX1, BNC2, TBX5, ID4 and HOXA5 and are not identifiable in the figure. Green triangles represent genes, the lines linking them represent a relationship between the expression of them. (**b**) - Mesenchymal Transcription Module and (**c**) - Immune Response Transcription Module. Master regulators of the bCSC phenotype inferred by MRA-FET in the bCS/CC dataset.

We wondered what could be the transcriptional programs controlled by these TFs. Consequently, to infer the regulons of each of these TFs, in the context of breast cancer, we applied a reverse engineering strategy on a larger dataset as required by the ARACNe algorithm (breast cancer NACT dataset, Supplementary Table S1)^19, 20^. Regulons of the 17 TFs resulting from the bCSCs consensus analysis (Table 1 and Supplementary Table S1) were used to build a network visualised in Cytoscape (Fig. 1a). In this independent dataset, the four regulons controlled by the TFs downregulated in bCSC (*SCML4*, *ZNF831*, *IKZF3*, and *SP140*) are largely overlapping and set apart from the regulons of TFs up-regulated in bCSC.

**Table 1:** bCSC specific Transcription Factors and Master Regulator.

Genes	Mode	FET P-Value	bCSC/CC	bCSC/Bulk
IKZF3	-	4.60E-152	−7.67	−2.46
SCML4	-	4.49E-147	−4.34	−6.62
SP140	-	9.48E-145	−7.89	−2.66
ZNF831	-	1.74E-138	−5.98	−2.02
BNC2	+	7.24E-22	3.76	2.13
HOXA5	+	4.37E-20	2.74	3.02
PRRX1	+	2.42E-18	7.31	3.22
SNAI2	+	1.23E-12	6.47	2.93
TBX5	+	8.01E-10	2.87	5
TWIST1	+	3.34E-09	5.05	3.31
ID4	+	9.06E-09	3.9	2.38

The 11 transcription factors differentially expressed and with the same pattern, up or down regulated, in both “bCSC/Bulk” and “bCSC/CC” datasets and selected as master regulators in the “bCSC/CC” dataset by MRA-FET. Mode = Positively (+) or negatively (−) correlated with the bCSC phenotype, FET p-Values = P value as possible Master regulator in MRA-FET analysis, bCSC/CC = Fold Change in the bCSC/CC dataset of the determined gene, bCSC/Bulk = Fold Change in the bCSC/Bulk dataset of the determined gene.

**Figure 2:**
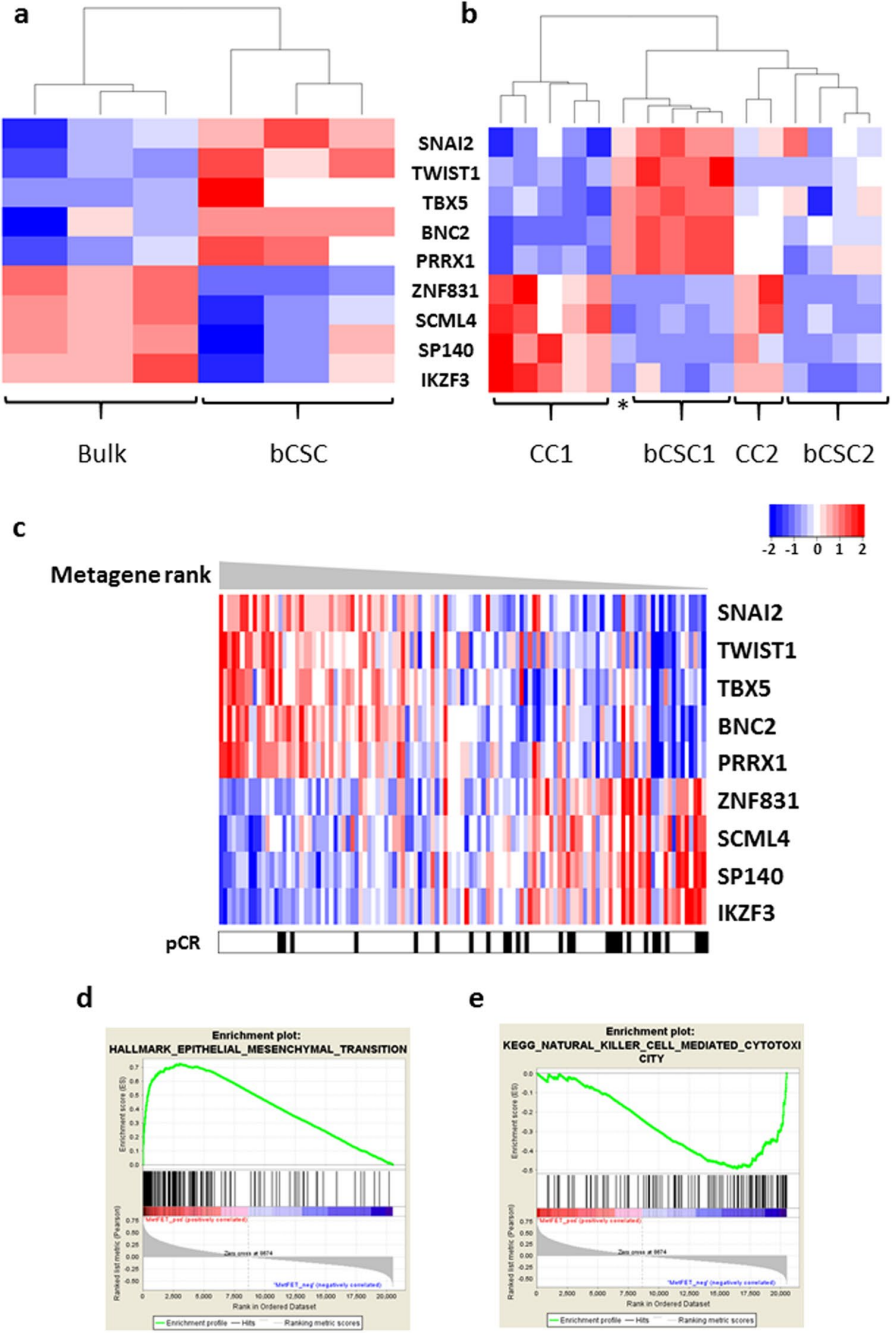
Transcription factor networks derived from breast cancer stem cells and its role in tumour tissues. Hierarchical clustering based in the expression of the TFs of the two modules: (**a**) - “bCSC/Bulk” dataset, (**b**) - “bCSC/CC” dataset, * refer to Biological Replicate 1 CC. (**c**) – NACT dataset showed an inverse coordinated expression of the TFs from the two networks and dataset are correlated to the complete disappearance of the tumour under treatment. When divided into two groups (58 vs 57 samples), there is a ratio of 3.57 of pCR in the group expressing less cancer stem cell TFs, p = 0.0008. **“d”** and **“e”** - GSEA datasets positively (“F), and negatively (“G”) correlated with high expression of the bCSC metagene composed of the 9 TFs in NACT dataset. (**d**) – Hallmark of Epithelial to Mesenchymal transition, (**e**) – KEEG Natural Killer Cell Mediated Cytotoxicity, evaluated by GSEA in a continuous way using Pearson metrics to rank the genes. p ≤ 0.05, FDR ≤ 0.01. bCSC = breast cancer stem cell, CC = cancer cell, pCR = Pathological Complete Response to Chemotherapy. Metagene Rank: Order of the samples as calculated by the expression of the TFs.

The *Master Regulator Analysis – Fisher’s Exact Test (MRA-FET)* algorithm computes the statistical significance of the overlap between the regulon of individual TFs (i.e., its ARACNE-inferred targets) and the signature of interest, giving as output the p-values computed by Fisher’s Exact Test. We used the list of differentially expressed genes from the “bCSC/CC” dataset as a signature for the breast cancer stem cell phenotype. MRA-FET indicates that regulons of 11 TFs contribute significantly to the bCSC signature: eight are positively correlated with the bCSC phenotype (*BNC2*, *PRRX1*, *TBX5*, *SNAI2*, *TWIST1*, *ID4*, *HOXA5*, and four are negatively correlated (*IKZF3*, *SCML4*, *SP140*, *ZNF831*) (Table 1). Of these 11 TFs, 9 are organised in two well-connected modules with the topology of logical circuits^16, 17^, Figure 1b and c display the regulatory circuits. One formed by *BNC2*, *PRRX1*, *TBX5*, *SNAI2*, *TWIST1* (Fig. 1b), and the other by *IKZF3*, *SCML4*, *SP140*, *ZNF831* (Fig. 1c). Consistently, clustering of samples using only these 9 TFs was sufficient to group the bCSC samples and separate them from the bulk tumour samples and other cancer cells (Fig. 2a and b).

### Inference of the biological processes associated with the bCSC Transcription Factor Modules expression

We constructed a bCSC metagene using the level of expression of these nine TFs (coefficient of 1 for bCSC TFs and −1 for immune module TFs) and ranked the samples in the NACT dataset and in TCGA-BRCA (Fig. 2c and Supplementary Figure S2). In these two gene expression datasets generated from distinct platforms (DNA-array and RNAseq), we again confirmed that the TFs *IKZF3*, *SCML4*, *SP140*, *ZNF831* were inversely (and co-ordinately) expressed compared to *BNC2*, *PRRX1*, *TBX5*, *SNAI2* and *TWIST1*. This was in accordance with the predicted structure of the regulons from the bCSCs database (Supplementary Figure S1 and Supplementary Table S1). It was of note that in the neoadjuvant dataset (NACT), low expression of the bCSC metagene correlated with pathological complete response to chemotherapy (3.57 x more frequent, Chi-square statistic = 11.2346, p = 0.0008) (Supplementary Table S3).

We evaluated the biological significance of the bCSC metagene, using the GSEA analysis (Fig. 2d,e, and Supplementary Figures S3–S6) in NACT and TCGA-BRCA datasets. This metagene was correlated with stem cell, EMT and aggressiveness signatures (Fig. 2d and Supplementary Figures S3 and S5), and inversely correlated with the immune response signature (Fig. 2e and Supplementary Figures S4 and S6). We designated the *SNAI2*, *TWIST1*, *BNC2*, *PRRX1* and *TBX5* TFs and their regulons as “mesenchymal transcription module” and the *SCML4*, *ZNF831*, *SP140* and *IKZF3* genes and their regulons as “immune response transcription module”.

**Figure 3:**
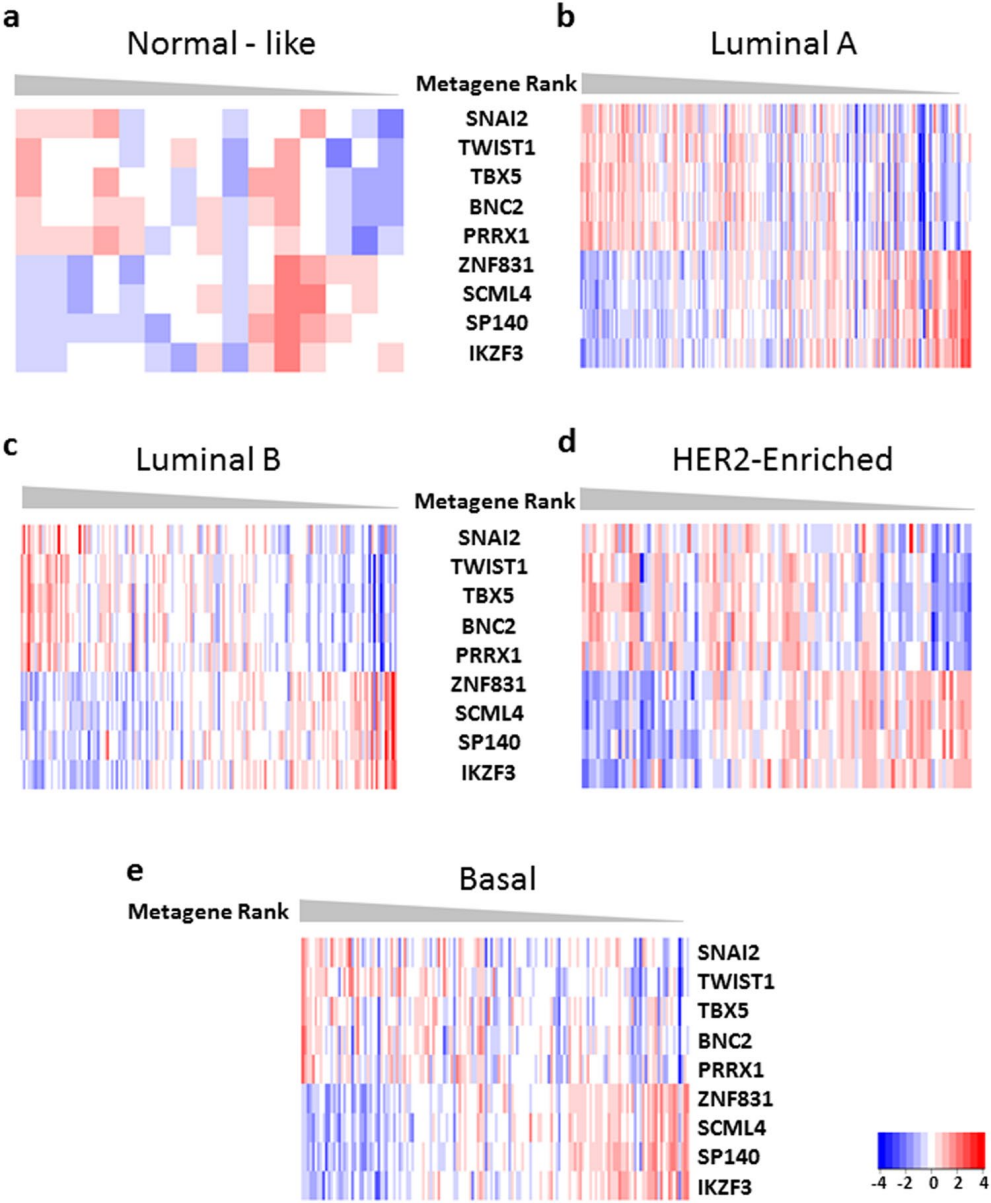
Coordinated expression of the TFs of the two modules is independent of the PAM50 classification in the TCGA-BRCA dataset - Coordinated expression of the TFs in the two validated networks. Heatmap of invasive ductal breast cancer tissue from females of the TCGA-BRCA dataset, ordered by the rank of the metagene of all TFs in the two validated networks. (a): 15 Normal-Like samples, (**b**): 189 Luminal A Samples, (**c**): 162 Luminal B Samples, (**d**): 107 Her2-enriched Samples, (**e**): 148 Basal Samples. Metagene Rank: Order of the samples as calculated by the expression of the TFs.

**Figure 4:**
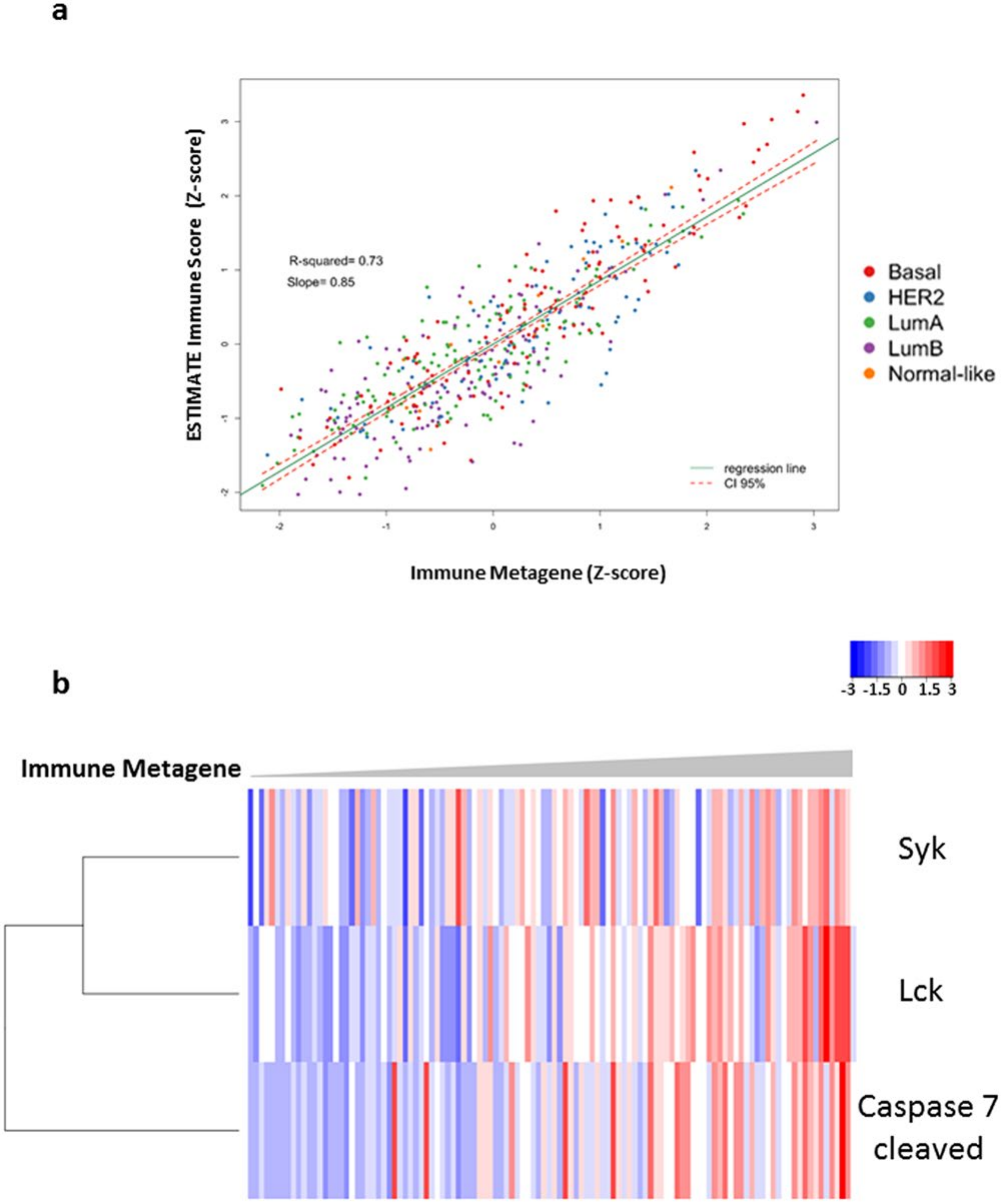
Expression of the immune module in TCGA-BRCA dataset is related to an increase in immune cells and in proteins necessary to an active immune response. (**a**) – Pearson Correlation between the ESTIMATE Immune score and the Immune metagene in the TCGA-BRCA dataset, r^2^ = 0.73, p < 0.01. (**b**) – Heatmap representing a semi-supervised clusterization of the protein levels of SYK, LCK and cleaved Caspase 7 in TCGA-BRCA dataset ordered by the rank of the Immune metagene. The correlation of the proteins level with the Immune metagene for SYK is r = 0.41, for Lck is r = 0.51 and for cleaved Caspase 7 r = 0.51. p < 0.01. Immune metagene = Metagene formed by the expression of IKZF3, SCML4, SP140 and ZNF831.

The analysis of the PAM50 breast cancer molecular subtypes in the TCGA-BRCA showed that the pattern of expression of the two groups of TFs and the related GSEA annotations were independent of the molecular subtype (Fig. 3 and Supplementary Figures S6–S10). The expression of the TFs in the immune transcription network correlated positively with that of the ESTIMATE immune score and of the immune response proteins in the TCGA-BRCA dataset (Fig. 4). The ESTIMATE immune score showed that increased expression of the immune module TFs was related to a greater infiltration of immune cells into tumour tissues (r^2^ = 0.73, p < 0.01), independently of the molecular subtype (Fig. 4a). Using RPPA data from TCGA-BRCA we found that the expression of the Tyrosine-protein kinases SYK and LCK as well as of the Caspase 7 protein (cleaved at D198) was correlated with the expression of the immune metagene, independently of the molecular subtype (Fig. 4b; r = 0.41, r = 0.51 and r = 0.51, respectively; p < 0.001; Supplementary Table S4). This strongly suggested that expression of the Immune response transcription module corresponded to increased infiltration of the tumour tissue by activated immune cells.

### Association of the bCSC Transcription Factor Modules expression with the clinical outcome of breast cancer patients

**Figure 5:**
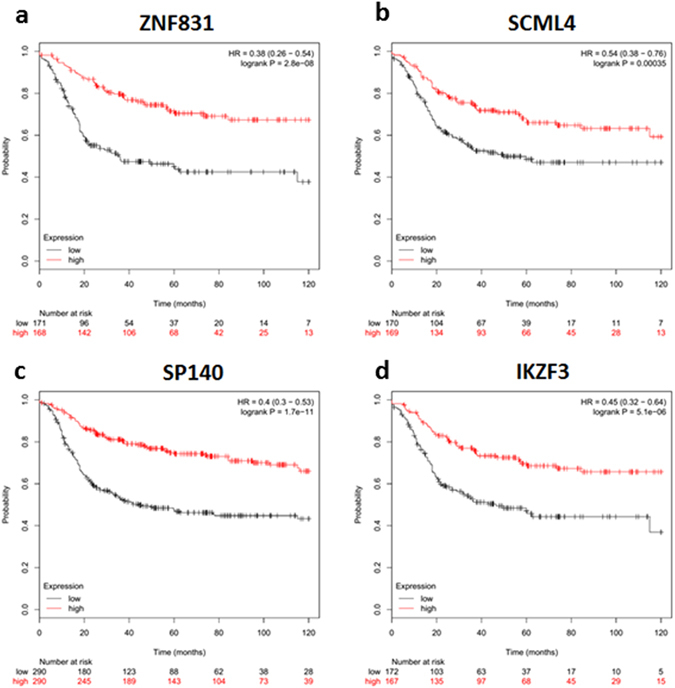
Survival plots depicting a better relapse free survival in breast cancer of the basal subtype with a high expression of: (**a**) - ZNF831, (**b**)- SCML4, (**c**) - SP140 and (**d**) - IKZF3.

We evaluated clinical associations with the activation of the two TF networks in a large compendium of breast cancer transcriptomes using the Kmplot tool^21^. We noted that expression of the mesenchymal TF module was not correlated to any patient outcome difference in the dataset, although the expression *TWIST1* and *BNC2* had an impact in the HER2-enriched and Basal subtypes (Supplementary Figures S11–S14). Contrastingly, expression of each TF from the immune response transcription module in the Basal tumours correlate with improved recurrence free survival (RFS) in a 10 year end point (Fig. 5; logrank p ≤ 0.01). It is also interesting to note that the high expression *ZNF831* has the lower hazard ratio (HR) in all molecular subtypes (Supplementary Figures S15–S17). Interestingly, in Luminal B patients expression of *IKZF3* was the only gene of the immune module that was significantly correlated with prolonged RFS (HR = 0.7 (0.51–0.96), logrank p = 0.028; Supplementary Figure S12).

### Impact on patient derived xenograft take

**Figure 6:**
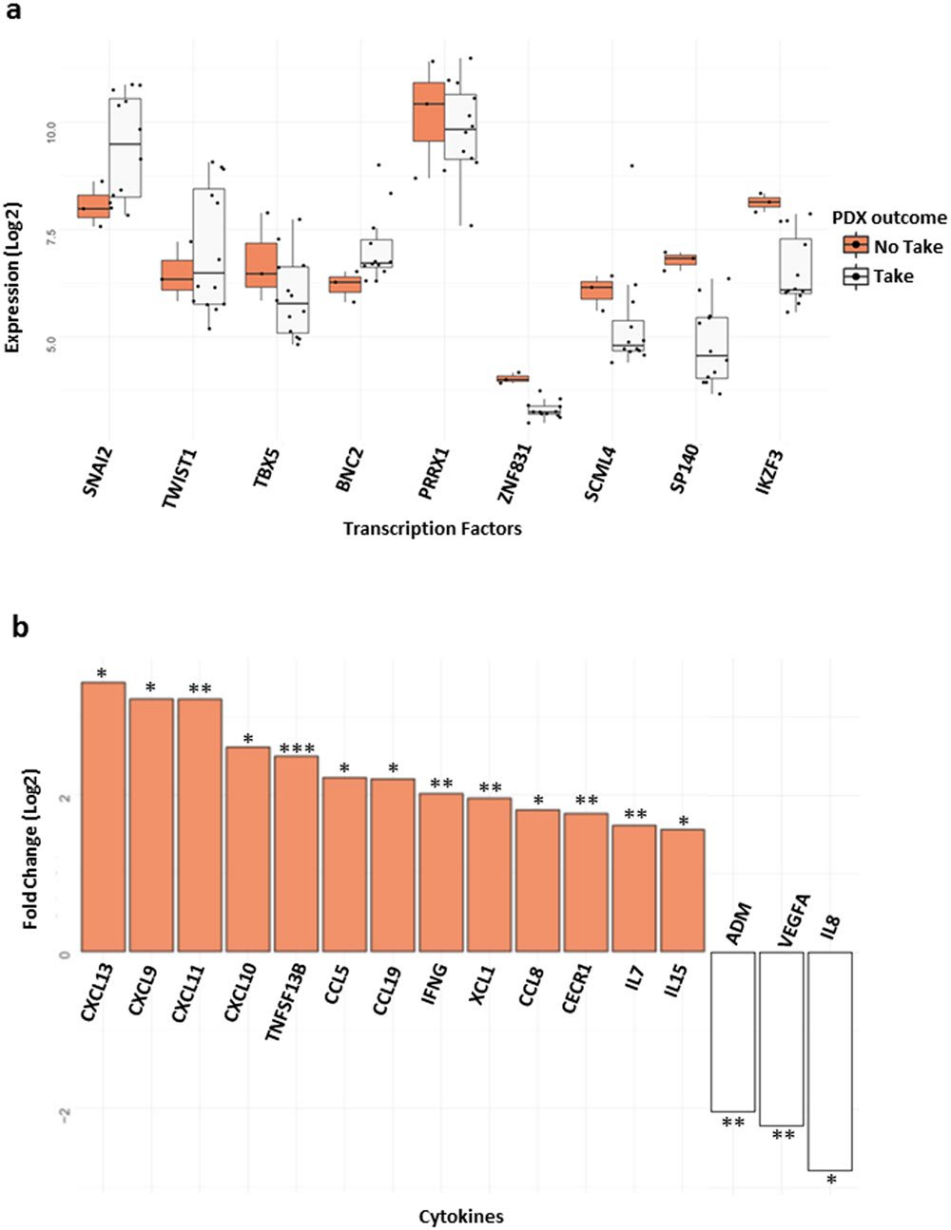
The association between TFs expression and the capacity to form patient-derived xenografts in Basl samples. (**a**) - Boxplot comparing the expression of the TFs of both modules in human tumour samples before implantation in the animal model ZNF831, IKZF3, and SP140 have a p < 0.05 in a t-test comparing “No take vs take” samples. TBX5, SNAI2, TWST1, BNC2 and SCML4 do not present statistical significant difference. (**b**) - histogram depicting the fold change of cytokines comparing “No take vs take” samples, with Fold Change > 1.5 and p < 0.05. “*” is for p < 0.05, “**” for p < 0.01 and “***” for p < 0.001.

Next, we evaluated the correlation of both signatures with the capacity of basal-like primary tumours to produce patient derived xenografts (PDX) upon graft on immunodeficient mice. Indeed, it has been  previously noted that tumours associated to early metastatic relapse were most likely to take in the mouse and produce a PDX line^20^. Remarkably, in this study we observed that expression of the immune response module was correlated to the failure of producing PDXs in Basal tumours (Fig. 6a). We stratified basal tumours in Take and No Take groups and noted that the expression of *ZNF831*, *IKZF3* and *SP140* was lower in the Take compared with the No Take group (p < 0.05). On the other hand, genes of the EMT/CSC signature TBX5, SNAI2, TWIST1, BNC2 and SCML4 did not show any significant expression difference between the two groups. However, the expression of the regulons of the mesenchymal module are enriched in the human tumours giving rise to tumour in mice and inversely, the regulons of the immune module are enriched in the No Takegroup (GSEA analysis based on rank, Supplementary Figure S18 and Supplementary Table S9 and Supplementary Figure S19 and Supplementary Table S10).

We then annotated the whole transcriptome differences between successfully Take and no take tumours, to delineate their prominent characteristics. Consistent with our previous result using other datasets, human tumours giving rise to tumour in mice present a mesenchymal profile (Supplementary Figure S20 and Supplementary Table S11) and No Take tumours depict an immune profile (Supplementary Figure S21 and Supplementary Table S12). In fact, we found a striking list of 20 Gene Ontology Biological process terms involved in immune response (Supplementary Figure S22 and Supplementary Table S13) suggesting an active tumoral immune response including cellular cytotoxicity and natural killer cell activity in No take samples. On the other hand, take samples exhibit an inactive adaptive and innate immunity profile (Supplementary Figure S23).

We then wonder what could be the influence of this active immune response in the human tumour when grafted on nude mice knowing that nude mice are T-cells deficient, have B-cells and proficient NK-cells. We hypothesize that chemokines/cytokines from human tumours may contribute to the activation of the remaining immune system in nude mice.

Accordingly, we analyse the cytokine expression changes (more than 1.5 log2, 2.8 fold, p < 0.05, t-test) between No take and Take tumours and found a large series of chemokine/cytokine overexpressed in the No take samples, among them IFNG, CXCL13, CXCl9, CXCl11, CXCL10 and CCL5 (Fig. 6b). Interestingly, most of these cytokine/chemokine belongs to the genes of the immune regulons (9/13) we previously defined (see Supplementary Table S1 for details, genes in bold in the table). These data lead us to propose the hypothesis that chemokine produced by the human tumour may stimulate the mice innate immune system, principally the NK cells, suggesting a rational explanation for the failure of engraftment in mice of the human tumour with highly active immune system (see Discussion).

## Discussion

Breast cancer is a heterogeneous disease^1, 3^ and a number of molecular classifications have been proposed in the past ten years in order to better characterize the disease and identify patients at risk of adverse outcome^3, 22^. The existence of a small population of cancer cells with self-renewal ability and increased tumour initiating capacity raised the hypothesis of the existence within malignant tumours of a subpopulation of CSCs possibly positioned at the top of the apex^5–7^. CSCs have been assigned traits that are believed to confer them increased capacity to cope with chemotherapy, such as semi-quiescence, increased expression of efflux pump, anti-apoptotic and DNA repair genes^2, 5, 7^. Furthermore, CSCs are believed to bear increased phenotypic plasticity and, thus, be associated to metastatic spread^6, 7, 9, 10^. These characteristics formed a strong rationale to identify CSCs and design therapeutic strategies that specifically target them.

In this study, based on transcriptome analyses from sorted ALDH1-positive cell subpopulations, we first identified a set of TFs differentially expressed between bCSC and bulk breast cancer. The regulons of these TFs were determined by reverse-engineering on a large dataset of breast cancer. Finally, we demonstrated that regulons from 9 TFs contributing significantly to the bCSC transcriptome, were organized in two networks showing inverse expression patterns. This pattern of expression could be confirmed in the NACT and the TCGA-BRCA datasets taken as a whole or stratified in breast cancer molecular subtypes.

The first network is controlled by the *SNAI2, TWIST1, BNC2, PRRX1* and *TBX*5 TFs. As 4 of these genes have been described as master TFs of epithelial to mesenchymal transition, we thus designated this network “mesenchymal transcription module”.

Remarkably, *SNAI2* and *TWIST1* were principal inputs in this module. *SNAI2* and *TWIST1* are major effectors of TGF-β induced EMT, which has been shown to be key to stem cell maintenance^2, 7, 23, 24^. Moreover, PRRX1 has been shown to induce EMT in a SNAI1-independent manner and to cooperate with TWIST1 to drive invasiveness^25^.

However, we could not show association of the mesenchymal module with patient outcome. This finding was in line with previous work concluding that *SNAI2, PRRX1 and TWIST1 were* poor predictors of recurrence-free survival (RFS) in breast cancer^26^.

The second network we identified comprised TFs SCML4, ZNF831, SP140 and *IKZF3* which are involved in active adaptive immune response. We, therefore, named this network “immune response transcription module”. *IKZF3* is the third member of the Ikaros family of transcription factors, which controls cell fate decision in haematopoiesis, via chromatin remodeling^27, 28^. *SP140* is part of the promyelocytic leukaemia protein nuclear body (PML-NB)^29, 30^. PML-NB is a tumour suppressor, linked to regulation of apoptosis and senescence^29, 31^. Recently *SP140* and *IKZF3* were recognized as master regulators of immune response in ovarian cancer^32^. S*CML4*, Sex Comb On Midleg-Like Protein 4, is related to the Polycomb group complexes which are known to act in chromatin remodelling and play important roles in stem cell plasticity, cell fate decision and cancer^33, 34^. Information about *ZNF831* is scarce in the literature^35^.

The expression of the TFs from this immune response module is correlated with an increase in the number of Tumour-infiltrating lymphocytes in the TCGA-BRCA dataset and the expression of activated Caspase-7, LCK and SYK proteins indicating an active immune response. Caspase-7 is an effector caspase whose expression culminates in apoptosis. It is activated by DNA-damage induced by chemotherapy^36, 37^. LCK is a member of the Src family of protein tyrosine kinases and is essential to the function of CD4 and CD8 signalling in mature T-cells^37^. SYK is critical for B cell antibody responses and memory B cells^38^ and also in innate immunity^39^. It is of note that SYK is expressed in normal luminal breast epithelial cells and in breast cancer cells its expression is associated to the luminal subtype and reduced invasiveness and metastasis^40^. We also observed increased expression of genes related to the presentation and processing of antigens, as well as increased T cell receptor pathway and natural killer cells mediated cytotoxicity. The activation of these pathways was reminiscent of that observed in transplanted organ rejection. The association with prolonged RFS strongly suggest an active immune editing mechanism modulating the tumour microenvironment^2, 41^. Aggressive tumours subvert the immune response, inducing naïve T cells to differentiate into Treg cells, rather than T helper 1 cells. This results in immune tolerance, which orchestrates the local adaptive immune response to the tumour^2, 41^.

We wondered what are the clinical implications of the expression of the immune module. Reduced expression of the mesenchymal module associated to increased expression of the immune module is associated with improved response to chemotherapy in NACT patients. This is in agreement with the data from Teschendorff *et al*.^23^ who reported an antagonism between Th1 response pathways and TGF-β pathway classically associated with EMT and cancer stem cells^2, 7, 23^. They demonstrated a better prognosis in patients with low activation of the TGF-β pathway and high activation Th1 response, in particular in Basal and HER2-enriched.

In a larger dataset, we demonstrated that the expression of the immune module was strongly associated with longer RFS in patients with Basal breast cancer. The good prognosis emphasizes the possible therapeutic or prognostic value of these genes.

PDXs are being used as preclinical models because they efficiently reflect the genetic and phenotypic structure of the tumours they originated from^42, 43^. The relationship of the tumour with its microenvironment, including the presence and active state of the hematopoietic cells, is one of the factors involved in the engraftment frequency and growth rate of implanted tumours^43^. Du Manoir and co-workers identified an IL8 signature in engrafted Basal-Like samples^42^, showing the importance of the immune system for engraftment. With the exception of SCML4, the other three TFs of the immune response network were differentially expressed in tumours that took upon grafting. This suggested that the levels of expression of these genes was related to the capacity to form a PDX, a process that is reminiscent of metastatic colonization^42–44^.

One puzzling point remains: what is the mechanism explaining why the very active immune system of human tumours contribute to the failure of take in the nude mice. Nude mice are mainly immune deficient but have functional NK cells that may participate to the destruction of the tumour cells^45^. We favour the hypothesis that cytokine/chemokines produced by the human tumour may activate the innate nude mice immune system via chemokines.

In fact, several cytokines were found highly overexpressed in the No take human tumours and most of these genes belong to the regulons of the immune module.

Among them IFNg, CXCL9, CXCL10, CXCL11, CCL5 and IL15 are noticeable. These three chemokines, CXCL9, CXCL10, and CXCL11 are IFNg inducible^46^ and are among the most potent attractant and activator of NK cells^47^, in addition, they have an angiostatic effect^46^. These three cytokines are present in mice and cross-reactivity have been demonstrated between these human and mice chemokines and their receptor CXCR3, in contrast to IFNg, that is inefficient in the mouse context^48^.

In xenograft models, CXCL9 and CXCL10 has been shown to inhibit Burkitt’s lymphoma tumours established in nude mice^49^. Intratumoral CCL 5 overexpression delays tumour growth and increases immune cell infiltration, including NK cell^50^. The expression of IL-15 was shown to remarkably retard the grown of prostate PC3 cells in the nude mice, an effect that was reverted by the inhibition of mice NK cells^51^.

In contrast, CXCL8/IL-8 which is over expressed in tumours of the Take group and CXCL12/SDF-1alpha are found in some mesenchymal regulons and have been shown to cooperatively promote invasiveness and angiogenesis in pancreatic cancer^52^. Also, IL-8 was reported to increased mammosphere formation and ALDH1+ cell population in breast cancer cell lines^53^.

We are aware that this hypothesis of chemokine from human tumours acting on mice NK, is only one of many possible explanations in this situation comprising complex populations of tumour cells and their interaction with a partially deficient immune system in nude mice. Further experiments will be needed to confirm this.

Altogether therapeutic response in NACT, recurrence free survival analysis in TCGA-BRCA and low expression of the immune module in primary tumours that successfully grafted on mice all point to the importance of immune system modulation in breast cancer. We, hence, propose that the balance between these networks controls the tumour microenvironment between a better differentiated and immune reactive state (a condition susceptible to immune equilibrium or elimination and better prognosis) and a more undifferentiated and less immune reactive state (a condition susceptible to tumour immune escape and progression)^2, 41^. This is in agreement with recent publications that pinpoint to a mechanistic link between EMT and immunosuppression^54, 55^.

System’s Biology has a deep impact in life sciences contributing in particular to the understanding on how biological circuits that control cell homeostasis^2, 15, 17^ are corrupted in cancer cells. Cancer stem cells are believed to form a subpopulation of cells within tumours that are more prone to initiate metastasis and resist to chemotherapy^5–7^. Transcription Factors are key elements at the molecular level^15–17^ of cell fate determination. Using this approach we determined the inverted expression patterns of the mesenchymal/bCSC and immune modules in breast cancer uncovering the existence of two subgroups of tumours differing in aggressiveness and outcome, with more pronounced effects in the Basal Subtype. Furthermore, we identified new partners (TBX5, BNC2, and PRRX1) of the well-known determinants of EMT and the bCSC, SNAI2 and TWIST1. The control of the expression of both modules has a relevant clinical potential and further studies are necessary to evaluate their possible impact on human health.

## Methods

### Patients

We prospectively sampled breast tumours from 40 patients at the Hospital das Clínicas, in the city of Ribeirão Preto, Brazil. The Ethics Committee on Human Research at the Hospital das Clínicas de Ribeirão Preto and the Medical School of Ribeirão Preto has approved the study with the protocol number: 2467/09. All experiments were performed in accordance with the Resolution no. 466/12 of the Brazilian National Health Council. Informed consent was obtained from all participants before their inclusion. One patient refused to be part of the study for religious reasons.

### Samples collection and Processing

All samples were obtained by percutaneous ultrasound-guided biopsy as a routine procedure in the hospital. One core fragment per patient was used in this study. The fragments were separated into halves. One-half was used for purification of bCSC and the other used as total tissue for paired comparison.

Fresh tissue samples were minced and then mixed in a solution of Collagenase IV (1 mg/ml) at 37 °C for 1 hour in agitation. After digestion, the cellular suspension was filtered and sedimented by centrifugation, and then resuspended in Aldefluor Assay Buffer©. The total number of live cells was estimated by trypan-blue exclusion using a haemocytometer in an inverted microscope.

To identify the bCSC and sort them by FACS we used the ALDEFLUOR© kit (Aldagen), the mouse anti-human EpCam conjugated with APC (eBioscience), and a pool of antibodies anti-Lin anti-CD31 and anti-CD45 conjugated with APC (eBioscience) was used to exclude hematopoietic cell lineage and endothelial cells. We considered de bCSC population the ALDH1high/LIN-/EpCam+ cells. The flow cytometry assay was performed in the FACSAria II (BD Biosciences, San Jose, CA) and the analysis of the data was performed using the FlowJo software (TreeStar, USA).

The total RNA was extracted using the Mirvana Kit (Ambion, USA) as specified by the manufacturer. We estimated the RNA quantity and purity by UV spectrometry by A220/A260 and A260/A230 ratios. RNA integrity was evaluated using the RNA 6000 Nano Kit, RNA 6000 Pico Kit and 2100 Bioanalyzer (Agilent Technologies, USA).

Of the initial 40 patient samples, for logistical problems we were not able to realise the cell sorting in 19 of them. From the 21 sorted samples, we had to exclude 18 samples: 2 because the diagnostic of the patients were not IDC, 4 because of RNA degradation, 11 because of the low quantity of RNA and 1 because of low quality of chip hybridization.

## Datasets

### Discovery Datasets

#### bCSC/Bulk dataset

We extracted the RNA from the patients’ samples, both from bCSC and from the bulk of the tumour, and analysed utilizing the GeneChip® Human Gene 2.0 ST Array (Affymetrix, USA) in the International Research Center (CIPE), A.C. of the Camargo Cancer Center, São Paulo, SP, Brazil. This analysis was performed with the transcriptome information from 3 samples (ER+/HER2+, HER2+ and TN) paired as bCSC against the bulk, and the two groups were analysed one against the other, as described below. The clinical data from patients is depicted in Supplementary Table S6. The dataset was deposited in GEO repository under the accession number GSE83811.

#### bCSC/CC dataset

The “bCSC/CC” dataset was taken from the public available GEO repository (GSE52327)^56^. It is composed of paired samples of 8 patients. The samples were divided into bCSC (ALDH1+/LIN-/ESA+) and cancer cells (CC, ALDH1-/LIN-/ESA+) and the two groups were analysed one against the other, as described below. The RNA extract was analysed with the Human Genome U133 Plus 2.0 Array chip (Affymetrix, USA). The Immunohistochemical status of the samples is described in Supplementary Table S7.

#### Neoadjuvant chemotherapy (NACT) dataset

The “NACT dataset” was taken from public available GEO repository (GSE32646). The dataset consists of transcriptome information acquired with Human Genome U133 Plus 2.0 Array chip (Affymetrix, USA) of 115 tissue samples from patients with breast cancer acquired by core biopsy prior to chemotherapy^19^. The patients were treated with neoadjuvant chemotherapy and divided by response two groups, the group with pathological complete response (pCR) and the group with non-pathological complete response (nCR)^19^. The Immunohistochemical status of the samples is described in Supplementary Table S8.

### Validation Datasets

#### TCGA - BRCA Dataset

From “The Cancer Genome Atlas” (TCGA), we used public available RNAseq data from Illumina platform. The normalised RNAseqV2 data from 1198 patients was downloaded and processed using the TCGA-Assembler package in R environment. Clinical, pathological and proteomic data were downloaded with the TCGA-Assembler package and we selected 621 samples with histological confirmed invasive ductal carcinoma from females. We classified the samples in the PAM50 subset using the “intrinsic.cluster” function in the “genefu” R package.

The proteomic Data were downloaded already normalised, in the level 3. The RPPA technology was used. The ESTIMATE is a method that uses gene expression signatures to infer the fraction of stromal and immune cells in tumour samples and its immune score were used as describe previously (check Supplementary material and methods).

### Survival Dataset

The survival analysis was made using the web tool “Kaplan-Meyer Plotter“^21^, which uses 4142 breast samples from the GEO repository, the technology used was the HGU133 Plus 2.0 array from Affymetrix. To make the survival curve we used the median of the expression of the SCML4, IKFZ3, SP140, ZNF831 genes.

#### Xenografts

We used the data from breast cancer samples generated in the Theillet group at the *Institut de Recherche en Cancérologie de Montpellier* (IRCM) for the building of a collection of PDXs^42^. We compared the initial transcriptome profile of human tumour samples before the implantation in the mouse, that have generated PDXs (Take samples) against those that did not (Not Take samples). The xenografts were generated by the implantation of a single fragment of fresh human tumour (approximately 8 mm3) into the inter-scapular fat pads of 3 to 4-week-old female Swiss-nude mice (NU/NU, Crl:NU-Foxn1nu Charles Rivers, France)^42^. The project was approved by the IRCM internal ethics committee and by the University of Montpellier animal ethics committee under registration number 1144.

#### Molecular Profile Analysis of the Datasets

We normalised the samples values by robust multi-array average (RMA) using the R package “affy” for GeneChip® Human Gene U133 Plus 2 and “oligo” package for GeneChip® Human Gene 2.0 ST. The annotation of the genes was made using the information accessible in the Affymetrix website (http://www.affymetrix.com/support/technical/annotationfilesmain.affx). We summarised the data by max strategy. Using the “limma” function on the POMELOS2 tool. We applied a paired t-test in the “bCSC/Bulk” and “bCSC/CC” datasets, then selected the transcription factor genes as described by Vasquerizas and colleagues in 2009^15, 16^ -with p ≤ 0.05, a fold change ≥2, considering the size of datasets, 6 and 16, respectively, we accept an FDR ≥ 0.2, and with the same pattern, up or down-regulated, in both datasets. The regulons were inferred using the ARACNE algorithm using genes selected from the intersection between DE genes in each dataset^15, 20^. We used the Adaptive Partitioning Algorithm with using p ≤ 0.01 correct by the number of markers as the threshold and not accepting DPI tolerance. Each regulon was analysed by Fisher’s exact test method of master regulator analysis (MRA-FET)^15^. Expression networks were constructed based on the relationships of the TFs inferred by ARACNE and confirmed by MRA-FET.

The value of the metagene, based on the expression of nine TFs evaluated as master regulators, were calculated in the NATC dataset. We used as a coefficient for each gene its fold change estimated in bCSC/CC dataset using the genefu package in R. We ordered the samples by that score and depicted them as a heatmap using the gplots package in R Hierarchical clustering was performed using the complete linkage method^.^ We divided the ordered dataset into two halves and calculated the difference in pathological complete response between them using Chi-square test. Finally, we used the score of the metagene as a continuous phenotype (using Pearson metrics) in GSEA analyses. Same methods were used to analyse the “TCGA-BRCA” dataset. The technical references are listed in the Supplementary document.

We downloaded the complete list of “cytokines and growth factors” from GSEA gene families documentation^57, 58^.

## Electronic supplementary material

Supplementary Methods, Figures and Tables
