# Supplementary material for: Transcription Factor Networks derived from Breast Cancer Stem Cells control the immune response in the Basal subtype
Source: Sci Rep. 2017 Jun 6;7:2851. doi: 10.1038/s41598-017-02761-6 (PMC5460106; doi:10.1038/s41598-017-02761-6)
Supplement: Supplementary file 1 — Supplementary Methods, Figures and Tables [file 41598_2017_2761_MOESM1_ESM.pdf]

Transcription Factor Networks derived from Breast Cancer Stem Cells control the immune response in the Basal subtype.

Silveira W.A.[1,2], Palma P.V.B.[3], Sicchieri R.D.[1], Villacis R.A.R[4,5], Mandarano L.R.M.[1], Oliveira T.M.G.[1], Antonio H.M.R.[1], Andrade J.M.[1], Muglia V.F.[1], Rogatto S.R.[4,6], Theillet C.[2,7,8], du Manoir S.[2,7 +*], Tiezzi D.G.[1,9 +*] .

[1] Ribeirão Preto Medical School, University of São Paulo, Brazil.

[2] INSERM U1194, 34298 Montpellier cedex 5, France;

[3] National Institute of Science and Technology in Stem Cell and Cell Therapy, Center for Cell Therapy and Regional Blood Center, Ribeirao Preto, Brazil

[4] Department of Clinical Genetics, Vejle Sygehus, Vejle, and Institute of Regional Health, University of Southern Denmark, Denmark

[5] Department of Genetics and Morphology, Institute of Biological Sciences, University of Brasília - UnB, Brasília, DF, Brazil

[6] Department of Urology, Faculty of Medicine, UNESP, Sao Paulo State University, Botucatu, Sao Paulo, Brazil

[7] Institut de Recherche en Cancérologie de Montpellier, Université de Montpellier, 34298 Montpellier cedex 5, France

[8] Institut de Cancérologie de Montpellier (ICM), 34298 Montpellier cedex 5, France.

[9] CISBi – Center for Integrative Systems Biology – Ribeirão Preto Medical School, University of São Paulo

*stanislas.dumanoir@inserm.fr / dtiezzi@usp.br

[+] these authors contributed equally to this work

Corresponding authors : **Tiezzi D.G. du Manoir S.**

Supplementary Materials and Methods

We used Metacore software[1], version V6.29.68613. We use as input the fold changes expression of Take vs No take human tumors (fold changes higher or lower than 1.5 log2). The pathway Immune response_Inhibitory PD-1 signaling in T cells is presented

The technical references are listed in the end of the document[2-13]

Supplementary Figures and Tables

A

SYMBOL	bCSC/Bulk	bCSC/CC
PRRX1	7.31	3.22
SNAI2	6.47	2.93
TWIST1	5.05	3.31
ID4	3.9	2.38
BNC2	3.76	2.13
GATA6	3.22	2.05
ZNF503	3.07	2.05
FOXF2	2.89	3.2
TBX5	2.87	5
HOXA5	2.74	3.02
HOXB3	2.48	2.57
TSC22D1	2.32	2.54
CREB3L1	2.17	2.99
SCML4	-4.34	-6.62
ZNF831	-5.98	-2.02
IKZF3	-7.67	-2.46
SP140	-7.89	-2.66

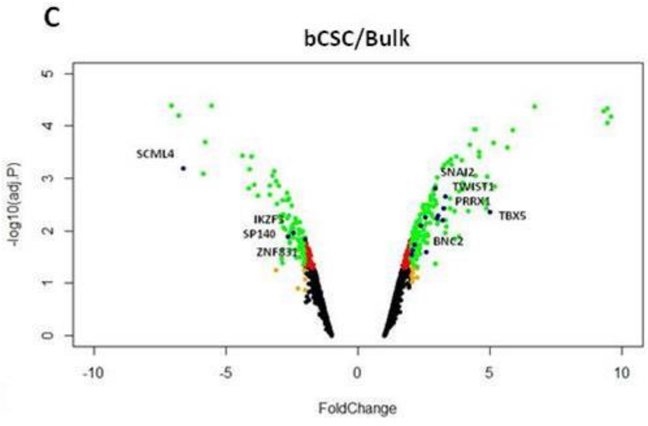

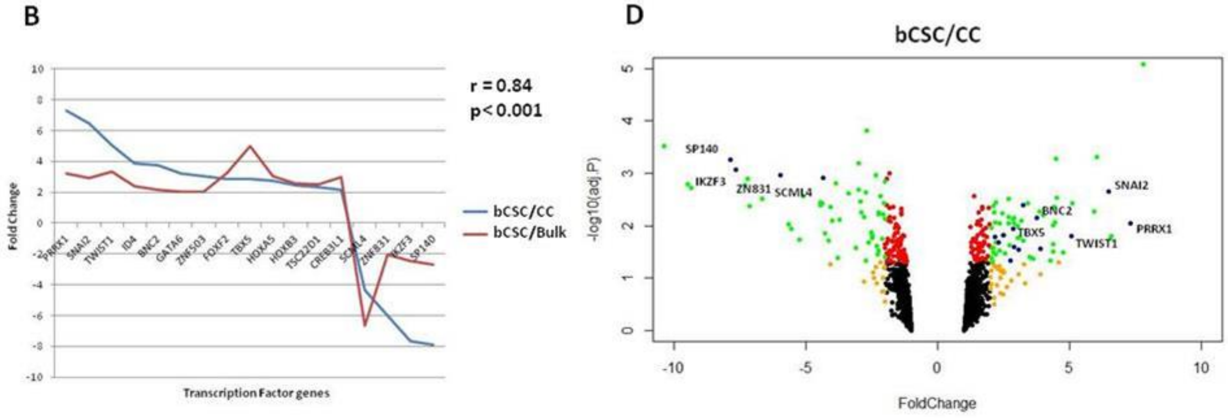

Supplementary Figure S1: bCSC specific Transcription Factors. A – Table containing the list with the 17 transcription factors differentially expressed going in the same direction in both “bCSC/Bulk dataset” and “Wicha - bCSC/CC” datasets, comparing the fold change presented in each dataset. Red = Up-regulated, Green = Down-regulated. p≤0.05. **B** – Graphic depicting the expression patterns of the TFs in both datasets, r = Pearson correlation coefficient. **C** and **D** – Volcano plots depicting the expression behavior, in fold change and p-value, of the TFs in **“**bCSC/Bulk” (**C**) and “Wicha - bCSC/CC” (**D**) datasets. Green dots = TFs with p≤0.05 and fold change ≥ 2.0, Red dots: TFs with p≤0.05, Yellow dots = TFs with fold change ≥ 2.0, Blue Dots = The 17 TFs in both datasets with p≤0.05 and fold change ≥ 2.0, with special attention to SCML4, ZNF831, SP140, IKZF3, SNAI2, TWIST1, BNC2, TBX5 and PRRX1. Black dots = All the others. Paired t-test, limma.

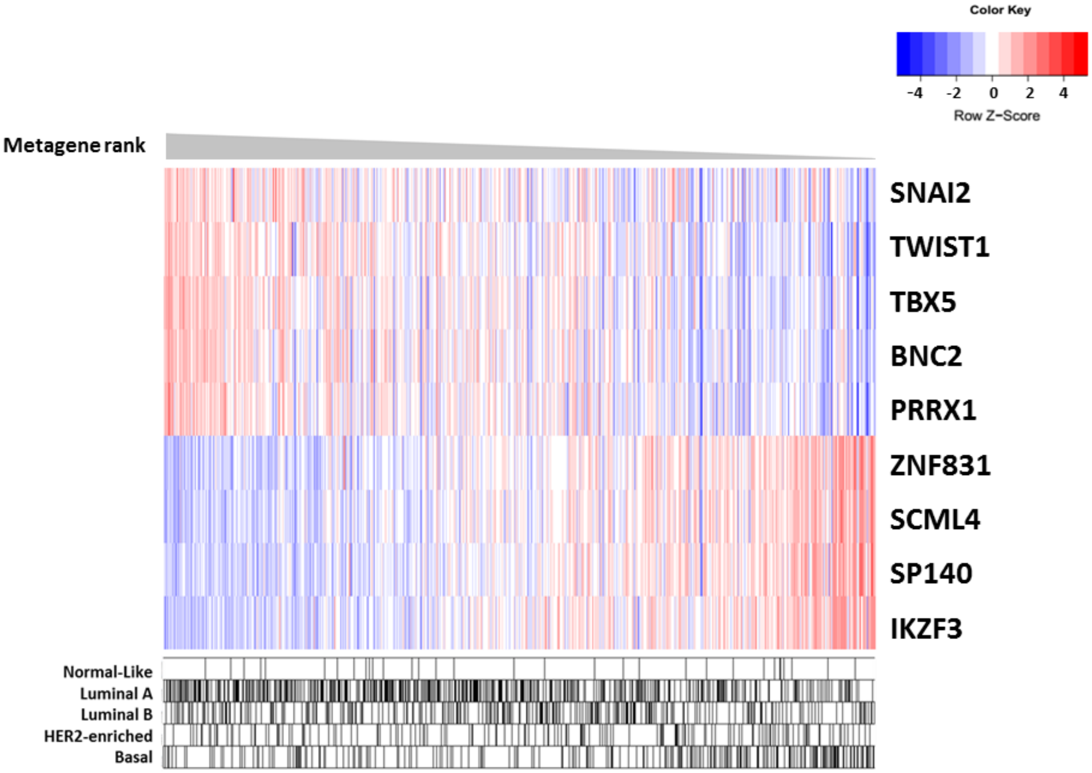

Supplementary Figure S2: TCGA-BRCA dataset - coordinated expression of the TFs in the two validated networks. Heatmap of 621 invasive ductal breast cancer tissue samples from females of the TCGA-BRCA dataset ordered by the rank of the metagene of all TFs in the two validated networks.

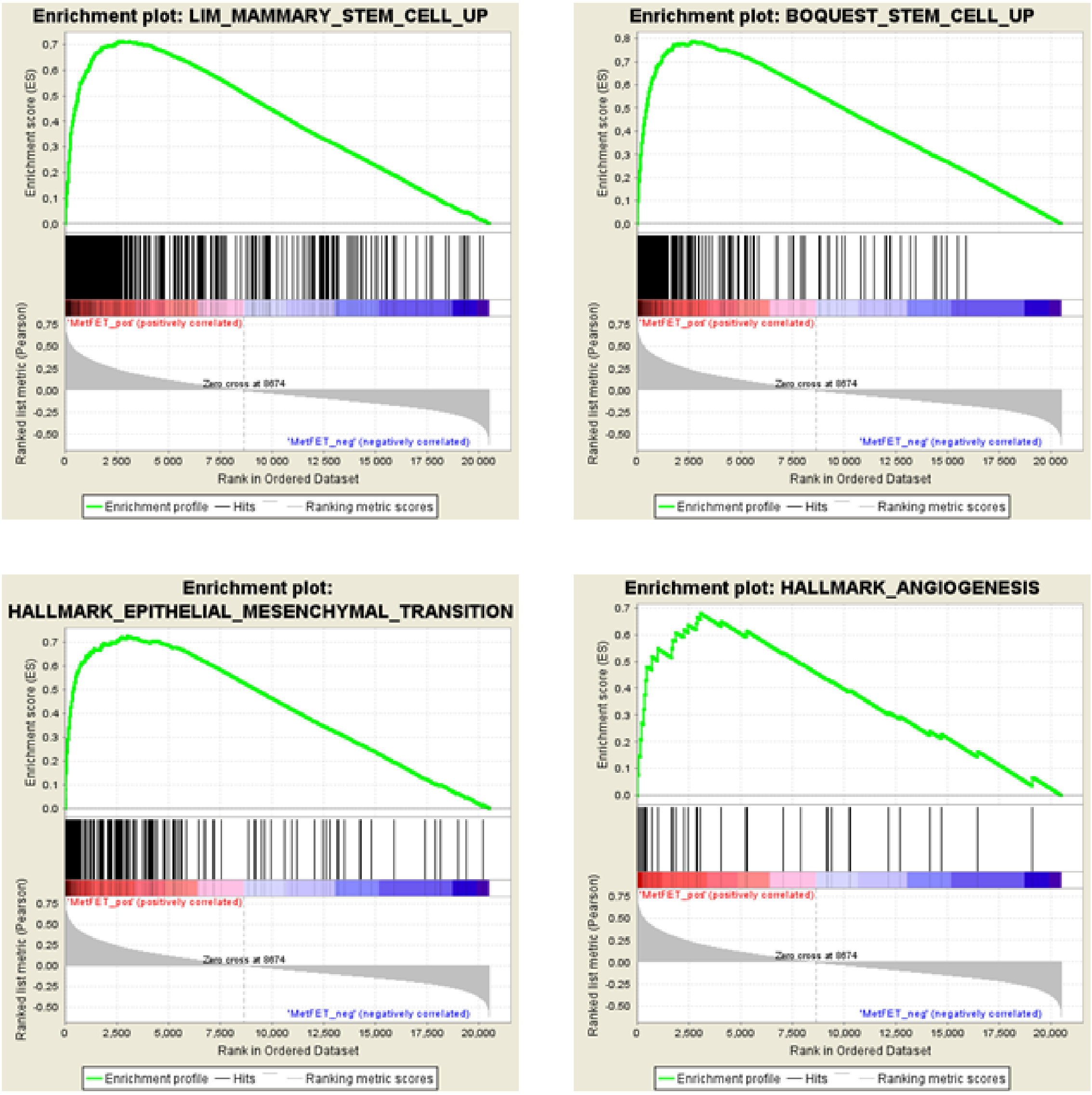

Supplementary Figure S3: GSEA datasets positively correlated with high values for the metagene in the NACT dataset. High expression of the “mesenchymal transcription factors module” and low expression of the “immune response transcription module”, evaluated by GSEA in an continuous way using Pearson metrics to rank the genes. p ≤ 0.05, FDR ≤ 0.01.

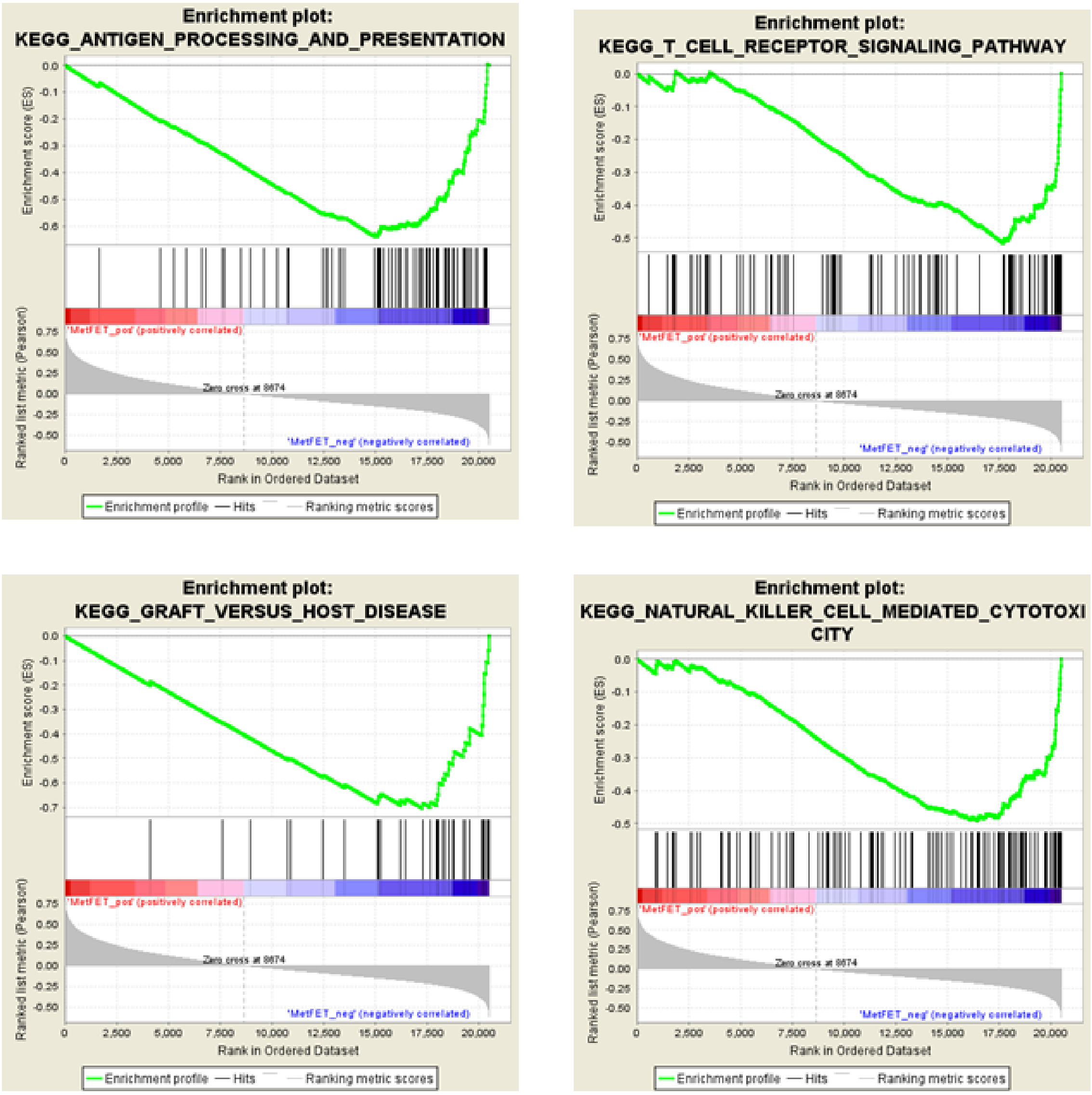

Supplementary Figure S4: GSEA datasets negatively correlated with high values for the metagene in the NACT dataset. Low expression of the “mesenchymal transcription factors network” and high expression of the “immune response transcription network”, evaluated by GSEA in a continuous way using Pearson metrics to rank the genes. p ≤ 0.05, FDR ≤ 0.01.

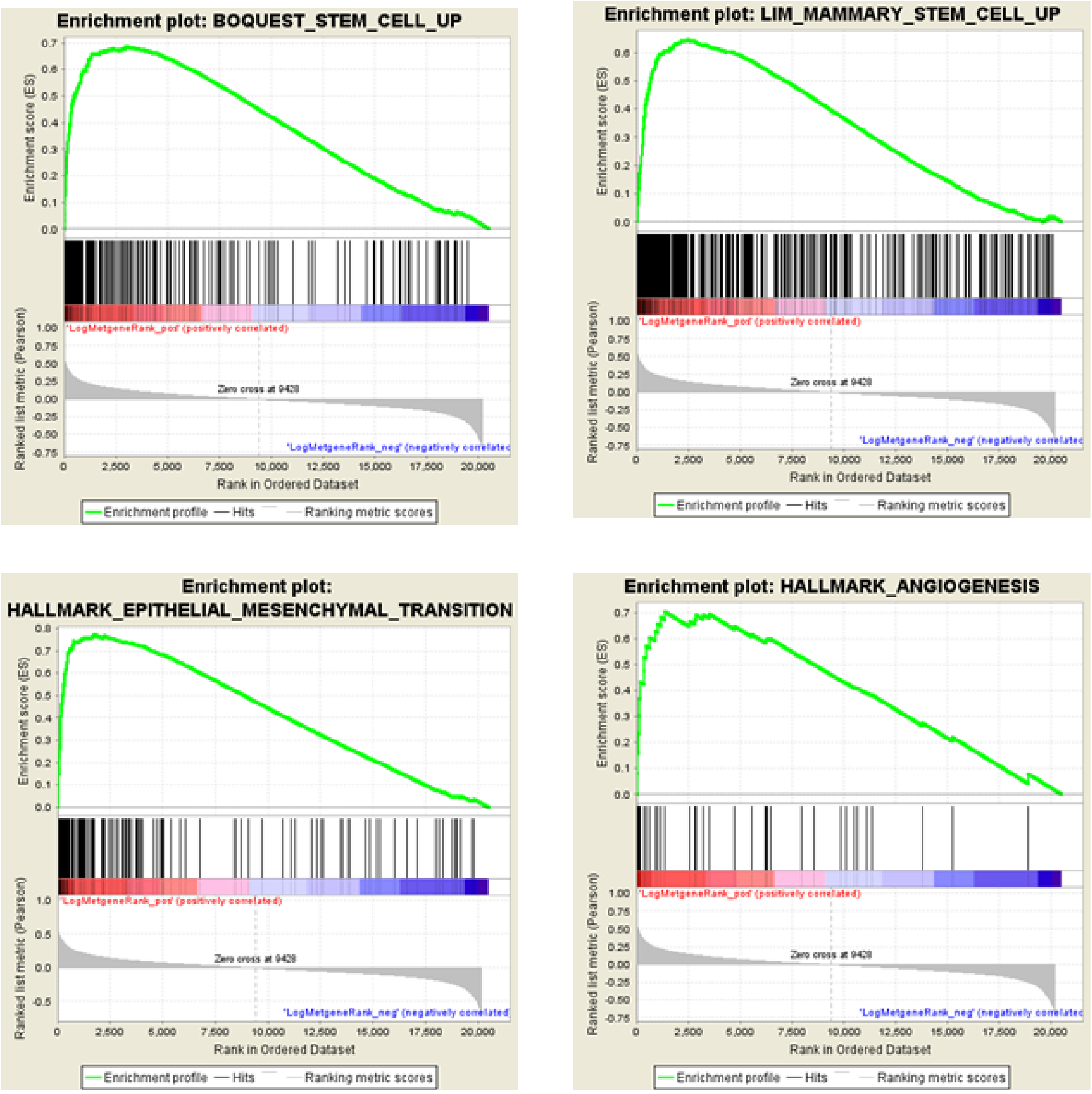

Supplementary Figure S5: GSEA analysis, TCGA-BRCA. Positive correlation. Snapshot of enrichment results of the positive correlation of GSEA analysis using the Hallmarks (H5) signatures in the 621 invasive ductal breast cancer tissue samples from females of the TCGA-BRCA dataset, ordered by the rank of the metagene of all TFs in the two validated networks. FDR < 0.05, nominal pvalue < 0.01.

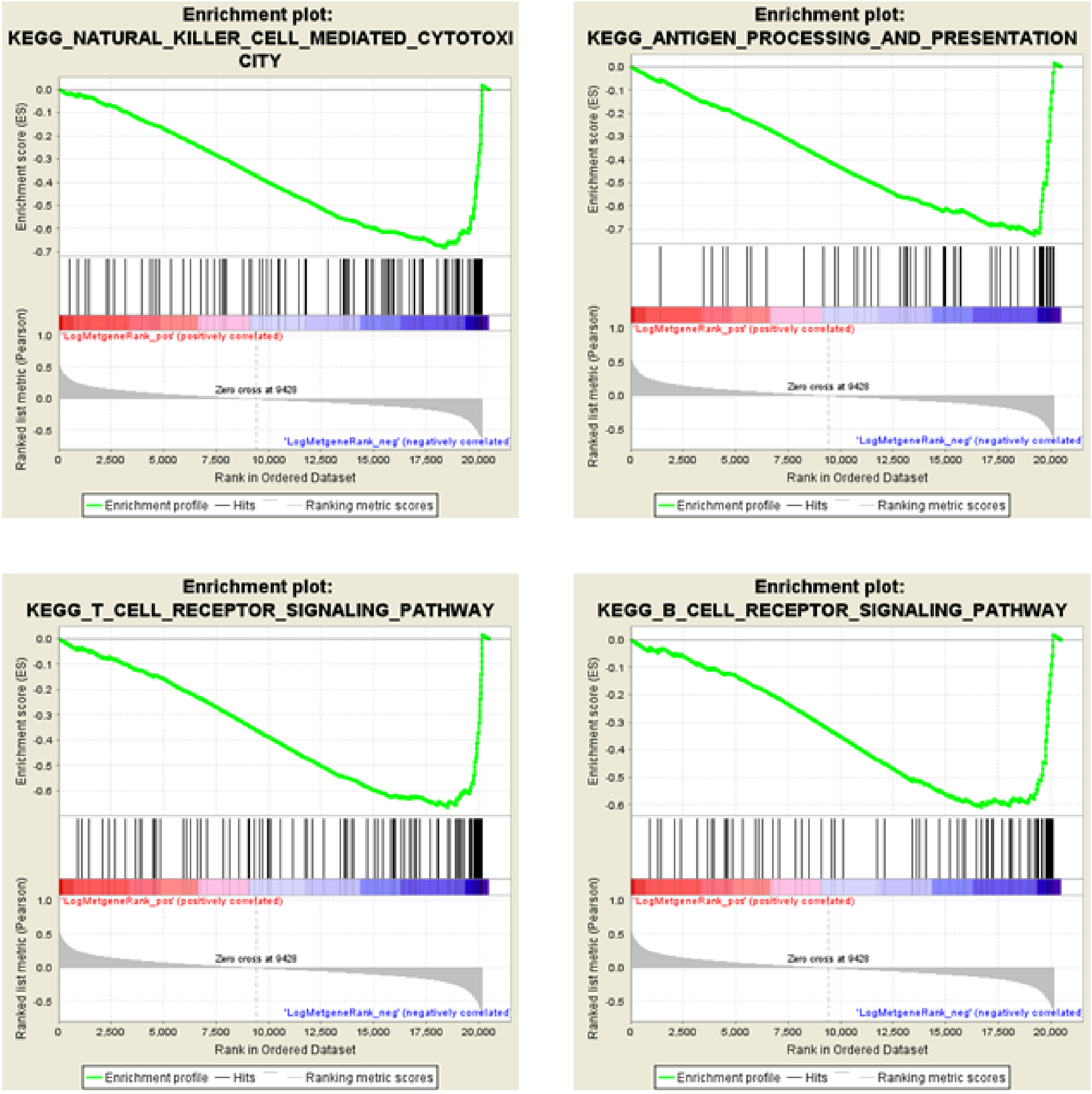

Supplementary Figure S6: GSEA analysis, TCGA-BRCA. Negative correlation. Snapshot of enrichment results of the positive correlation of GSEA analysis using the KEGG signatures in the 621 invasive ductal breast cancer tissue samples from females of the TCGA-BRCA dataset, ordered by the rank of the metagene of all TFs in the two validated networks. FDR < 0.05, nominal pvalue < 0.01.

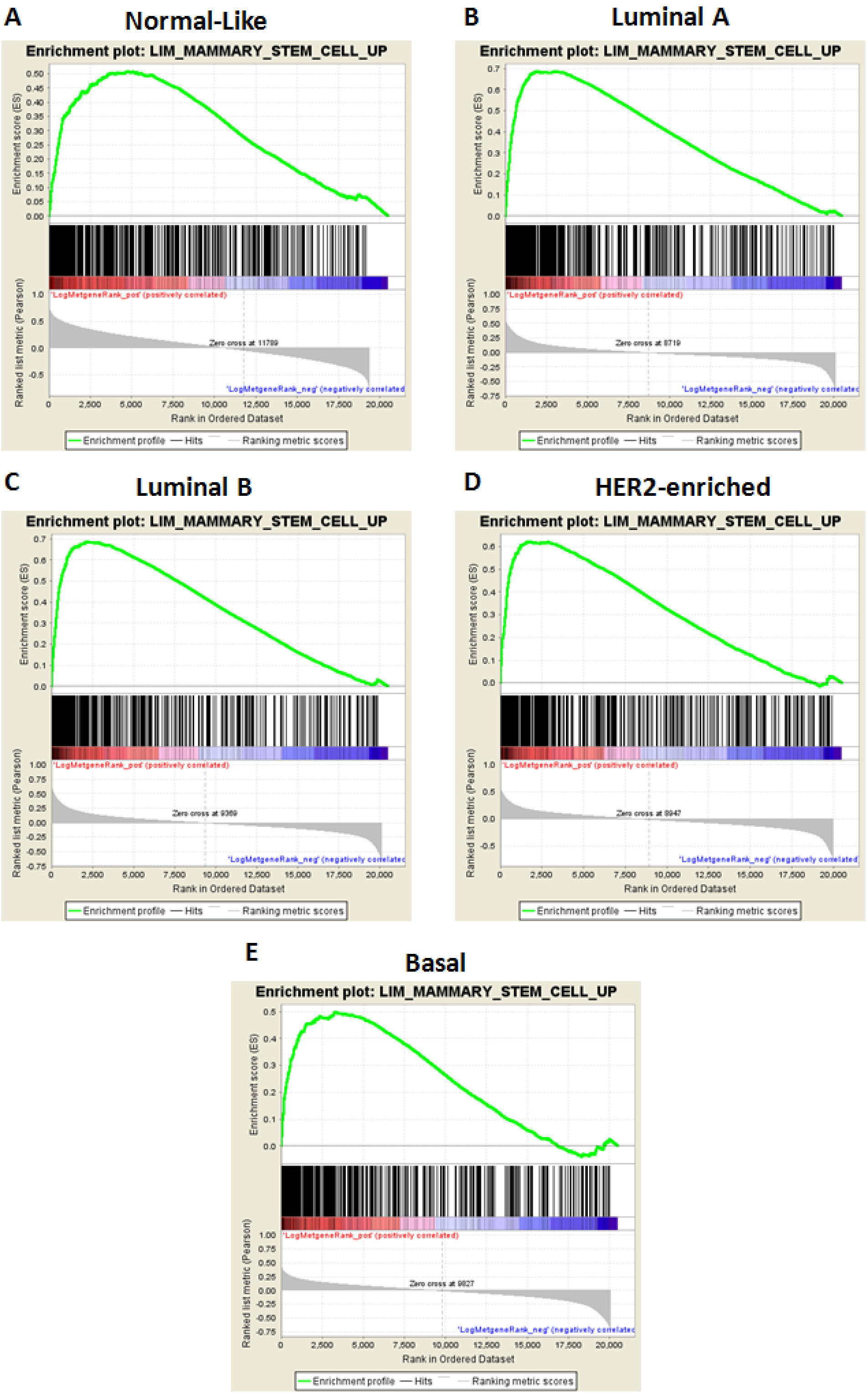

Supplementary Figure S7: Mammary stem cell signature. GSEA analysis, PAM50. TCGA-BRCA.

Positive correlation. Snapshot of enrichment results of the positive correlation of GSEA analysis using the "LIM mammary stem cell up signature" in cancer tissue samples from females of the TCGA-BRCA dataset, ordered by the rank of the metagene of all TFs in the two validated networks. **A: Normal-Like**, 32 Samples, $p \leq 0.1$, FDR ≤ 0.25. **B: Luminal A,** 404 samples FDR ≤ 0.01, nominal pvalue < 0.01. **C: Luminal B**. FDR ≤ 0.005, nominal pvalue < 0.001. **D: HER2-enriched** FDR < 0.05, nominal pvalue < 0.01, **E: Basal**: FDR ≤ 0.1, nominal pvalue < 0.05.

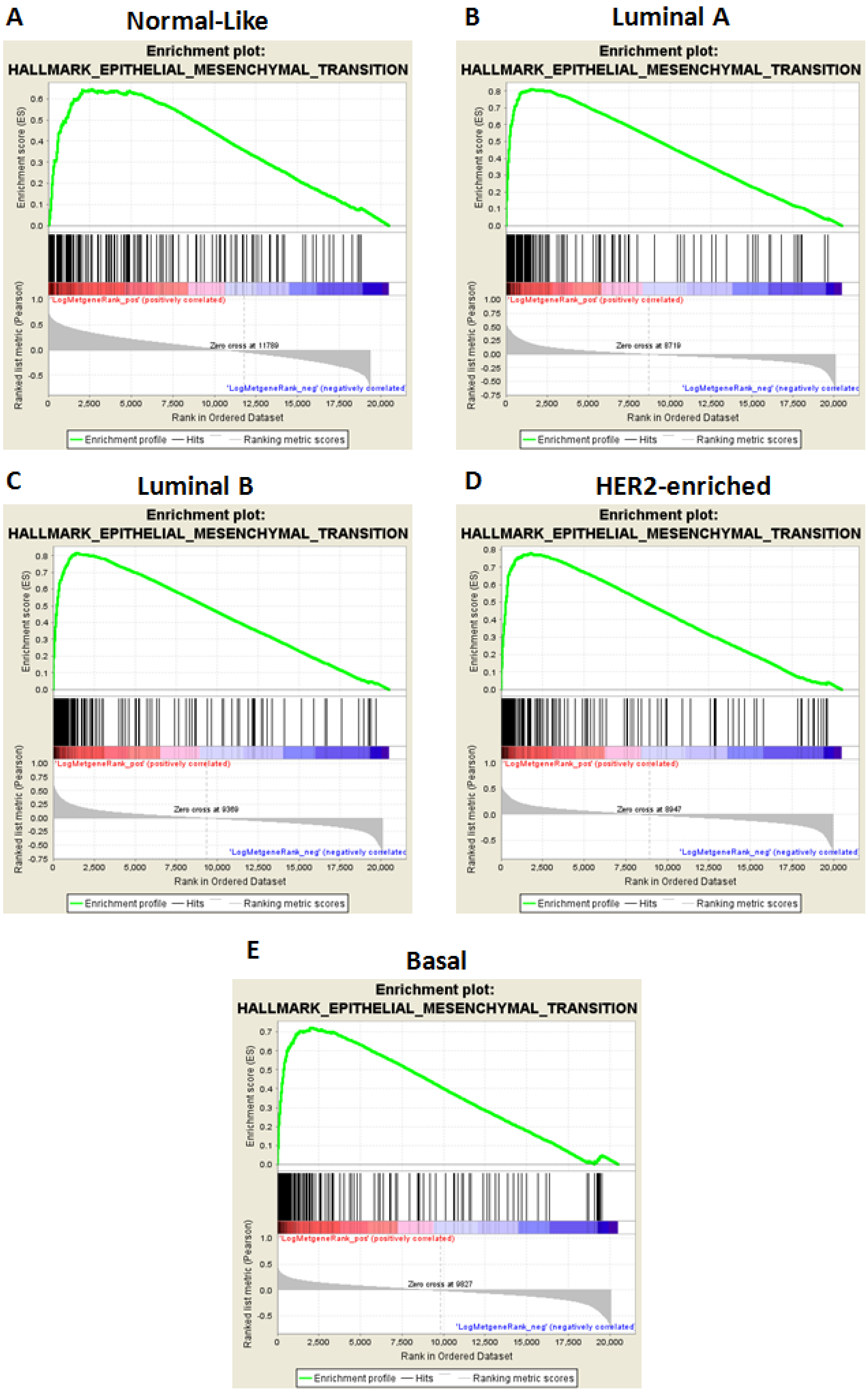
A
Normal-Like
Enrichment plot:
HALLMARK_EPITHELIAL_MESENCHYMAL_TRANSITION
Enrichment score (ES)
Ranked list metric (Pearson)
'LogMetgeneRank_pos' (positively correlated)
Zero cross at 11789
'LogMetgeneRank_neg' (negatively correlated)
Rank in Ordered Dataset
Enrichment profile
Hits
Ranking metric scores
B
Luminal A
Enrichment plot:
HALLMARK_EPITHELIAL_MESENCHYMAL_TRANSITION
Zero cross at 8719
C
Luminal B
Enrichment plot:
HALLMARK_EPITHELIAL_MESENCHYMAL_TRANSITION
Zero cross at 9369
D
HER2-enriched
Enrichment plot:
HALLMARK_EPITHELIAL_MESENCHYMAL_TRANSITION
Zero cross at 8947
E
Basal
Enrichment plot:
HALLMARK_EPITHELIAL_MESENCHYMAL_TRANSITION
Zero cross at 9827

Supplementary Figure S8: EMT signature. GSEA analysis, PAM50. TCGA-BRCA. Positive correlation. Snapshot of enrichment results of the positive correlation of GSEA analysis using EMT Hallmark signature in cancer tissue samples from females of the TCGA-BRCA dataset, ordered by the rank of the metagene of all TFs in the two validated networks. A: Normal-Like, 32 Samples, $p<0.05$. B: Luminal A, 404 samples FDR ≤ 0.001, nominal pvalue < 0.001. C: Luminal B. FDR ≤ 0.005, nominal pvalue < 0.001. D: HER2-enriched FDR < 0.005, nominal pvalue < 0.001, E: Basal: FDR < 0.01, nominal pvalue < 0.01.

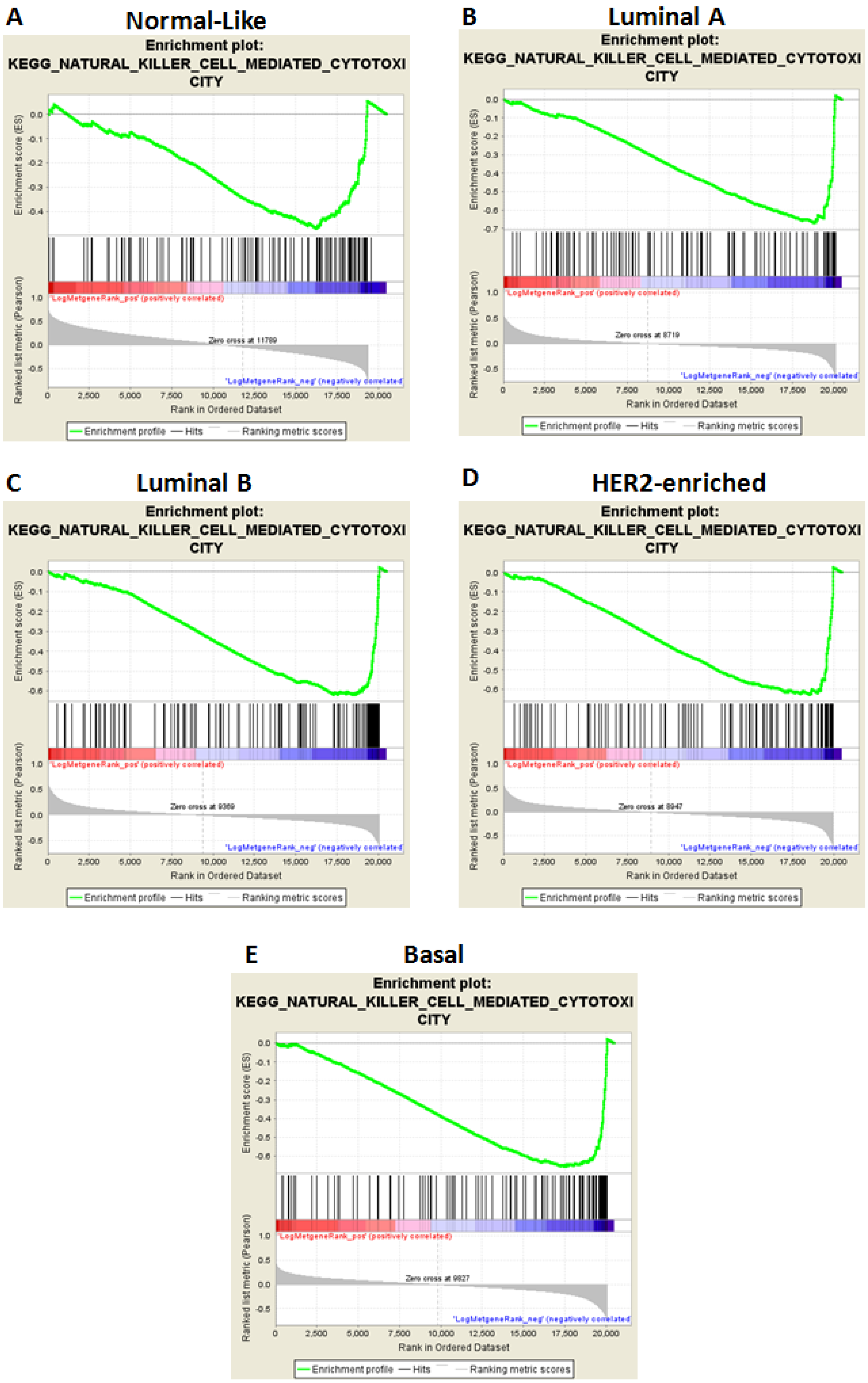
A
Normal-Like
Enrichment plot:
KEGG_NATURAL_KILLER_CELL_MEDIATED_CYTOTOXICITY
B
Luminal A
Enrichment plot:
KEGG_NATURAL_KILLER_CELL_MEDIATED_CYTOTOXICITY
C
Luminal B
Enrichment plot:
KEGG_NATURAL_KILLER_CELL_MEDIATED_CYTOTOXICITY
D
HER2-enriched
Enrichment plot:
KEGG_NATURAL_KILLER_CELL_MEDIATED_CYTOTOXICITY
E
Basal
Enrichment plot:
KEGG_NATURAL_KILLER_CELL_MEDIATED_CYTOTOXICITY
Enrichment score (ES)
Ranked list metric (Pearson)
Rank in Ordered Dataset
Enrichment profile
Hits
Ranking metric scores

Supplementary Figure S9: Natural killer cell mediated cytotocity signature. GSEA analysis, PAM50. TCGA-BRCA. Negative correlation. Snapshot of enrichment results of the negative correlation of GSEA analysis using NK cell mediated cytotocity signature in cancer tissue samples from females of the TCGA-BRCA dataset, ordered by the rank of the metagene of all TFs in the two validated networks. **A: Normal-Like**, 32 Samples, $p<0.05$. **B: Luminal A,** 404 samples FDR ≤ 0.01, nominal pvalue < 0.001. **C: Luminal B**. FDR ≤ 0.005, nominal pvalue < 0.001. **D: HER2-enriched** FDR < 0.001, nominal pvalue < 0.001, **E: Basal**: FDR < 0.001, nominal pvalue < 0.01.

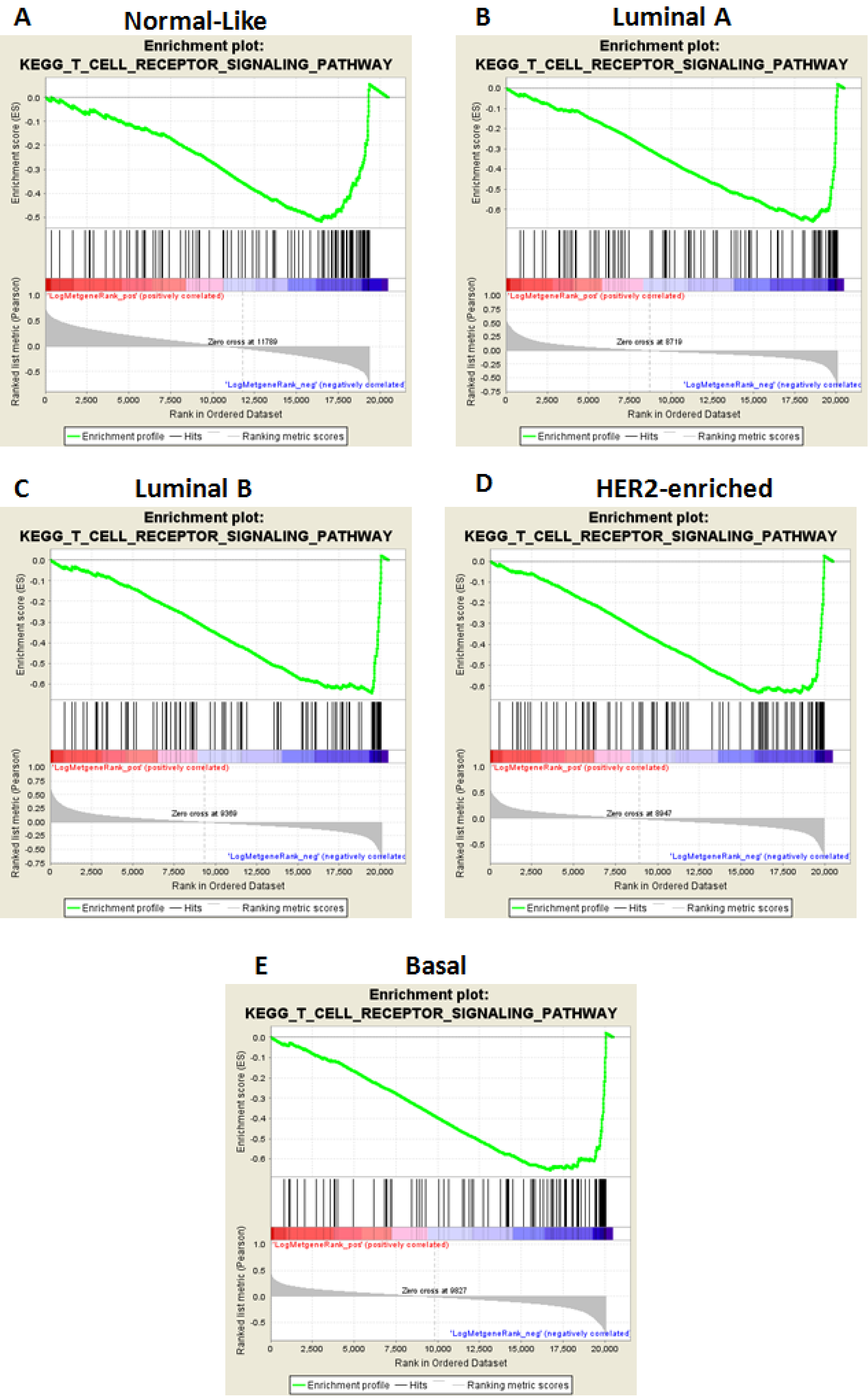

A
Normal-Like
B
Luminal A
C
Luminal B
D
HER2-enriched
E
Basal
Enrichment plot:
KEGG_T_CELL_RECEPTOR_SIGNALING_PATHWAY
Enrichment score (ES)
Ranked list metric (Pearson)
Rank in Ordered Dataset
Enrichment profile
Hits
Ranking metric scores

Supplementary Figure S10: KEGG T cell Receptor Signaling Pathway Signature . GSEA analysis, PAM50. TCGA-BRCA. Negative correlation. Snapshot of enrichment results of the positive correlation of GSEA analysis using **EMT** Hallmark signature in cancer tissue samples from females of the TCGA-BRCA dataset, ordered by the rank of the metagene of all TFs in the two validated networks. **A: Normal-Like**, 32 Samples, FDR ≤ 0.1, p<0.01. **B: Luminal A,** 404 samples FDR < 0.001, nominal pvalue < 0.001. **C: Luminal B**. FDR ≤ 0.005, nominal pvalue < 0.001. **D: HER2-enriched** FDR < 0.001, nominal pvalue < 0.001. **E: Basal**: FDR < 0.01, nominal pvalue < 0.001.

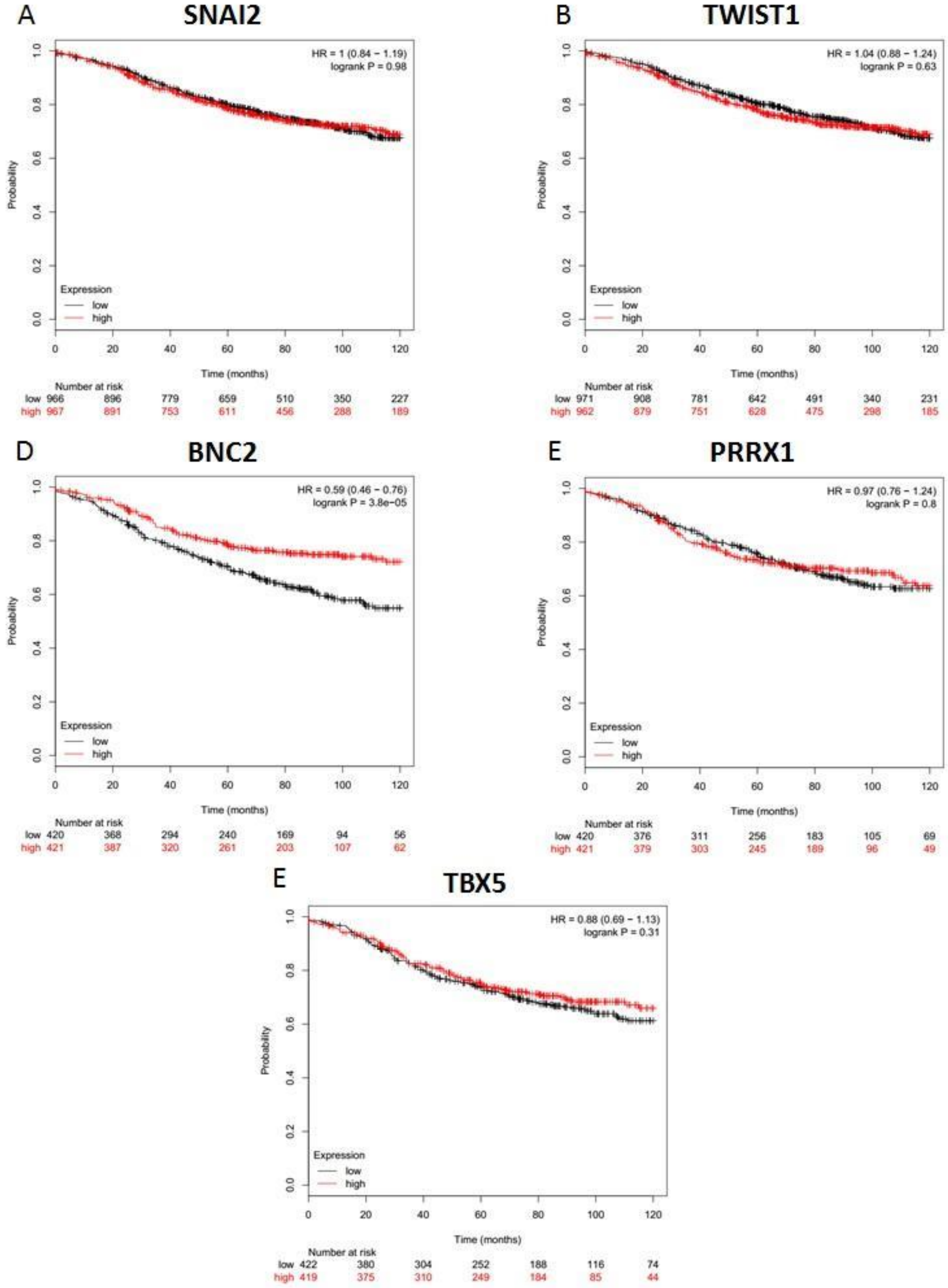
A
SNAI2
HR = 1 (0.84 − 1.19)
logrank P = 0.98
Probability
Expression
low
high
Time (months)
Number at risk
low 966 896 779 659 510 350 227
high 967 891 753 611 456 288 189
B
TWIST1
HR = 1.04 (0.88 − 1.24)
logrank P = 0.63
Probability
Expression
low
high
Time (months)
Number at risk
low 971 908 781 642 491 340 231
high 962 879 751 628 475 298 185
D
BNC2
HR = 0.59 (0.46 − 0.76)
logrank P = 3.8e−05
Probability
Expression
low
high
Time (months)
Number at risk
low 420 368 294 240 169 94 56
high 421 387 320 261 203 107 62
E
PRRX1
HR = 0.97 (0.76 − 1.24)
logrank P = 0.8
Probability
Expression
low
high
Time (months)
Number at risk
low 420 376 311 256 183 105 69
high 421 379 303 245 189 96 49
E
TBX5
HR = 0.88 (0.69 − 1.13)
logrank P = 0.31
Probability
Expression
low
high
Time (months)
Number at risk
low 422 380 304 252 188 116 74
high 419 375 310 249 184 85 44

Supplementary Figure S11: Survival Plots (RFS) depicting the relapse free survival in breast cancer of the **Luminal A** Subtype relation with the expression of: **A -** SNAI2, **B-** TWIST1, **C** – BNC2 , **D** – PRRX1 and TBX5.

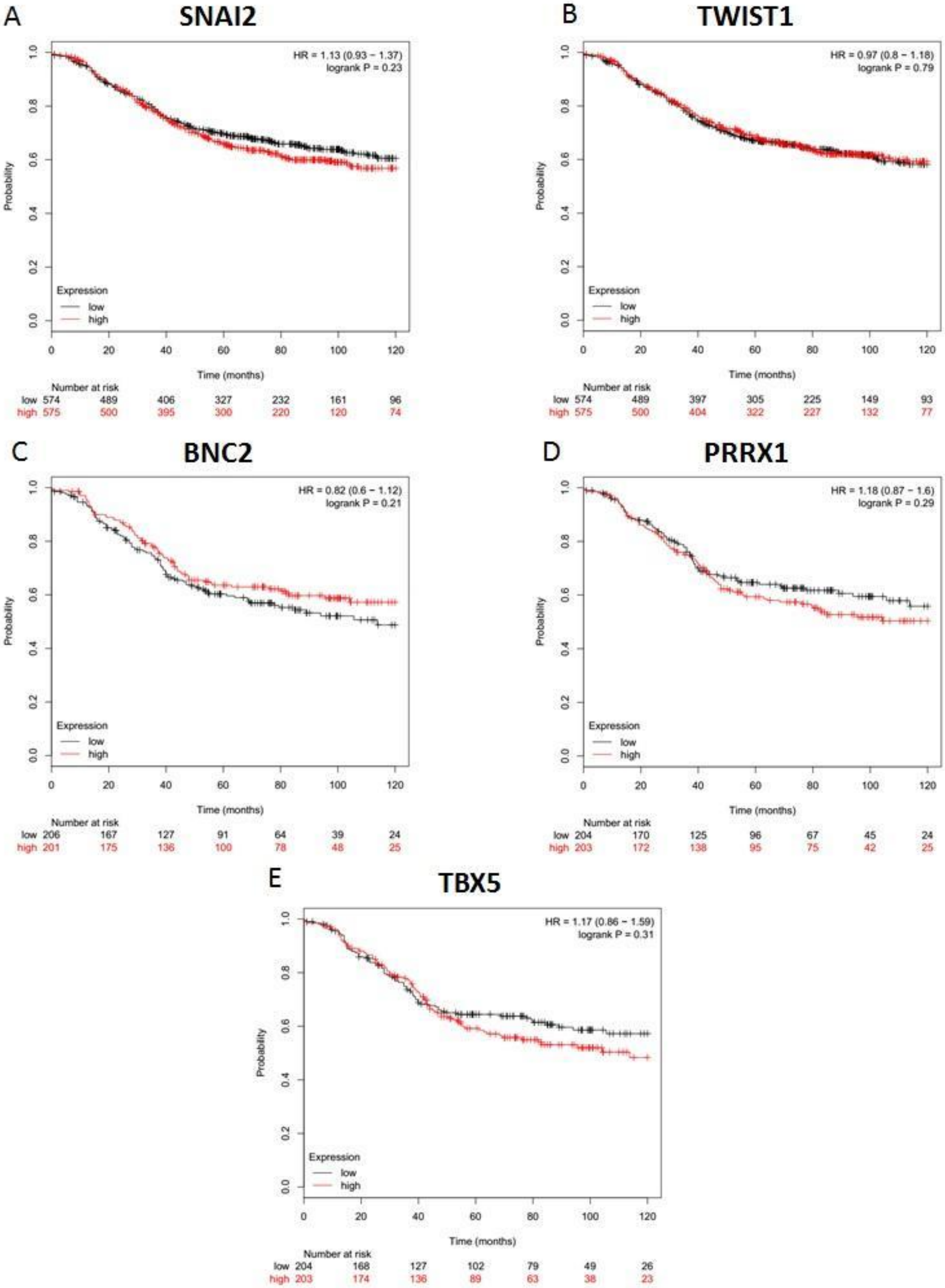

A
SNAI2
HR = 1.13 (0.93 − 1.37)
logrank P = 0.23
Probability
Time (months)
Expression
low
high
Number at risk
low 574 489 406 327 232 161 96
high 575 500 395 300 220 120 74
B
TWIST1
HR = 0.97 (0.8 − 1.18)
logrank P = 0.79
Number at risk
low 574 489 397 305 225 149 93
high 575 500 404 322 227 132 77
C
BNC2
HR = 0.82 (0.6 − 1.12)
logrank P = 0.21
Number at risk
low 206 167 127 91 64 39 24
high 201 175 136 100 78 48 25
D
PRRX1
HR = 1.18 (0.87 − 1.6)
logrank P = 0.29
Number at risk
low 204 170 125 96 67 45 24
high 203 172 138 95 75 42 25
E
TBX5
HR = 1.17 (0.86 − 1.59)
logrank P = 0.31
Number at risk
low 204 168 127 102 79 49 26
high 203 174 136 89 63 38 23

Supplementary Figure S12: Survival Plots (RFS) depicting the relapse free survival in breast cancer of the **Luminal B** Subtype relation with the expression of: **A -** SNAI2, **B-** TWIST1, **C** – BNC2 , **D** – PRRX1 and TBX5.

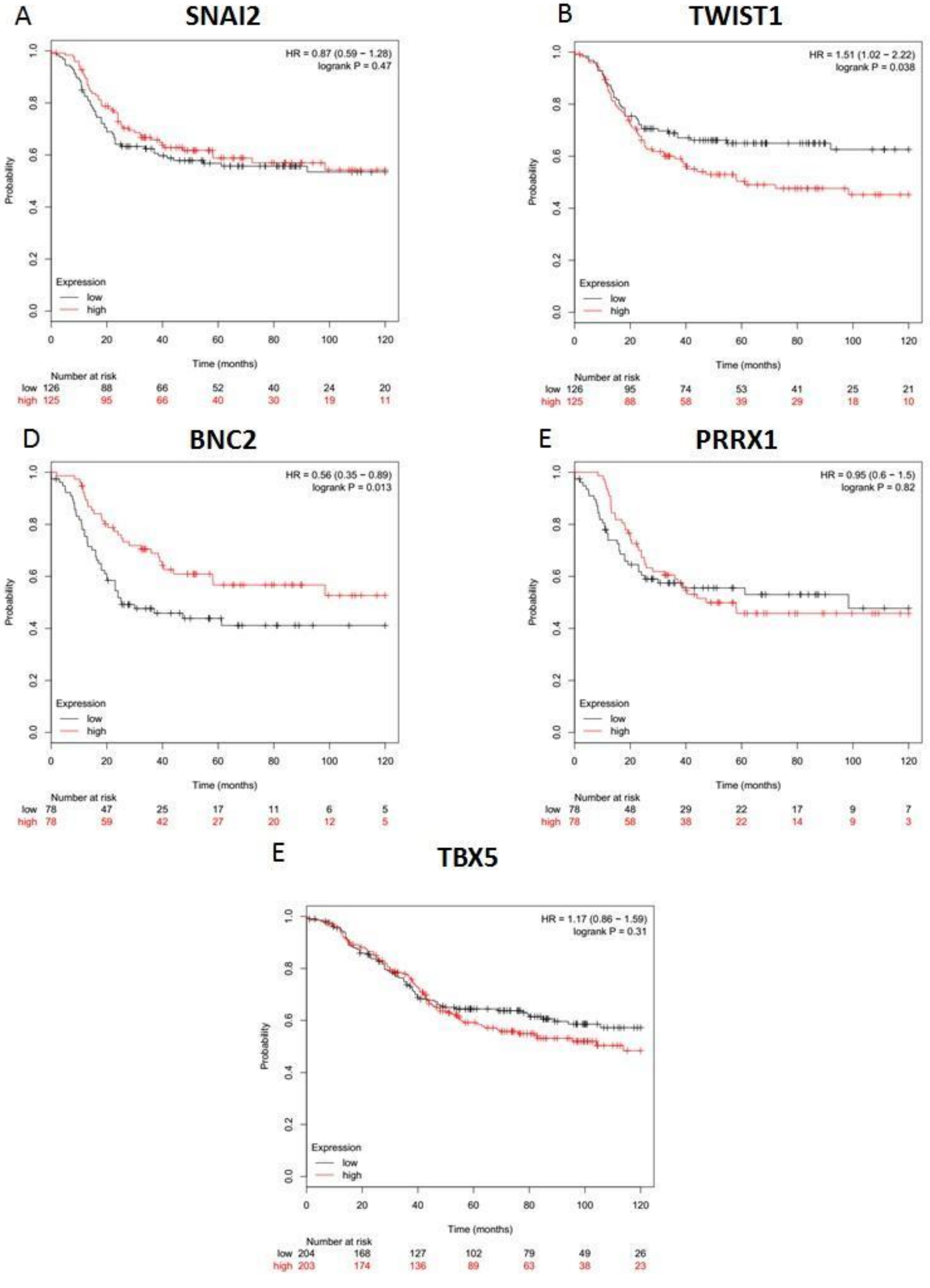
A
SNAI2
HR = 0.87 (0.59 − 1.28)
logrank P = 0.47
Probability
Expression
low
high
Time (months)
Number at risk
low 126 88 66 52 40 24 20
high 125 95 66 40 30 19 11
B
TWIST1
HR = 1.51 (1.02 − 2.22)
logrank P = 0.038
Probability
Expression
low
high
Time (months)
Number at risk
low 126 95 74 53 41 25 21
high 125 88 58 39 29 18 10
D
BNC2
HR = 0.56 (0.35 − 0.89)
logrank P = 0.013
Probability
Expression
low
high
Time (months)
Number at risk
low 78 47 25 17 11 6 5
high 78 59 42 27 20 12 5
E
PRRX1
HR = 0.95 (0.6 − 1.5)
logrank P = 0.82
Probability
Expression
low
high
Time (months)
Number at risk
low 78 48 29 22 17 9 7
high 78 58 38 22 14 9 3
E
TBX5
HR = 1.17 (0.86 − 1.59)
logrank P = 0.31
Probability
Expression
low
high
Time (months)
Number at risk
low 204 168 127 102 79 49 26
high 203 174 136 89 63 38 23

Supplementary Figure S13: Survival Plots (RFS) depicting the relapse free survival in breast cancer of the **HER2-Enriched** Subtype relation with the expression of: **A** - SNAI2, **B-** TWIST1, **C** – BNC2 , **D** – PRRX1 and TBX5.

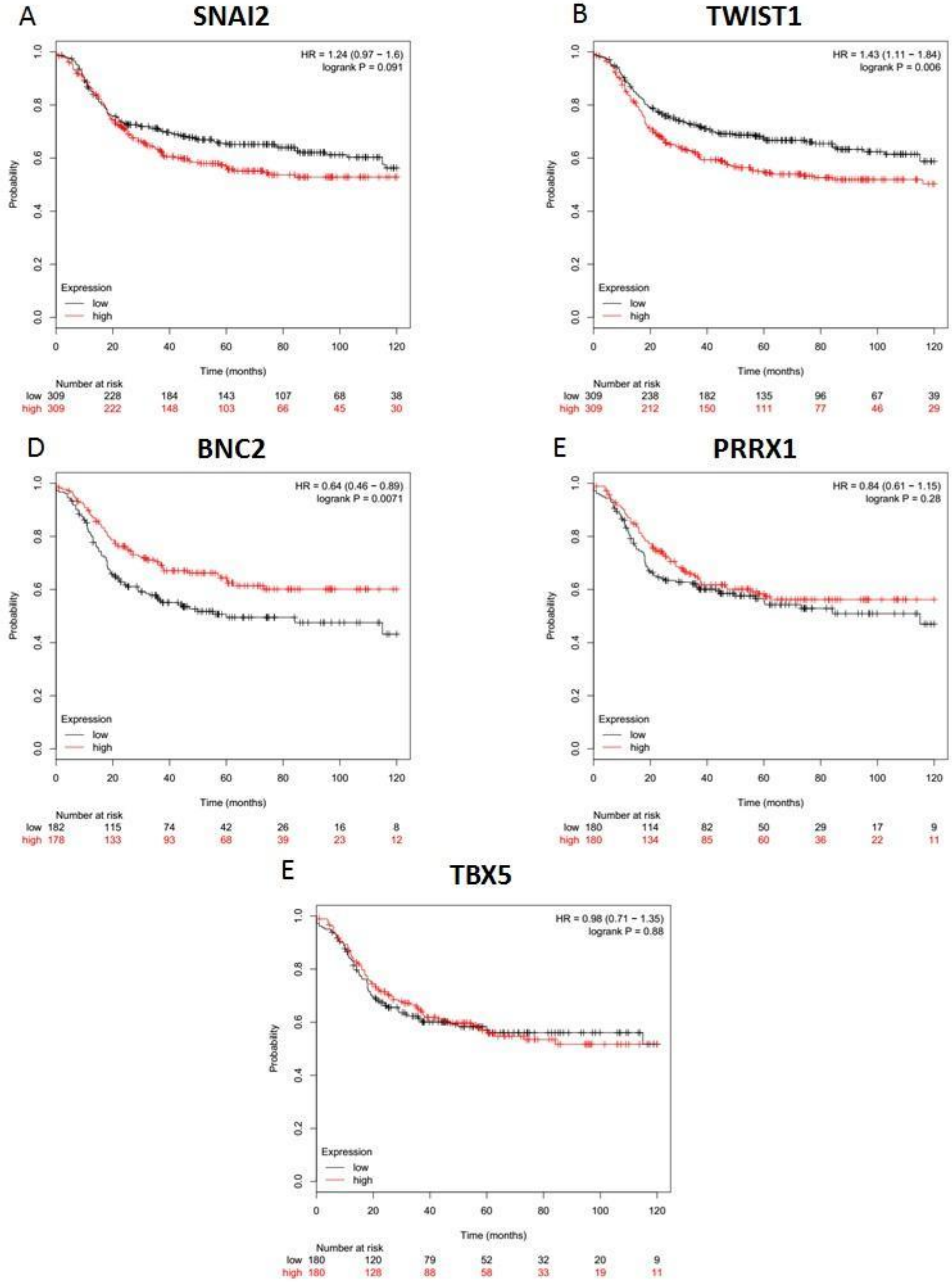
A
SNAI2
HR = 1.24 (0.97 − 1.6)
logrank P = 0.091
Probability
Time (months)
Expression
low
high
Number at risk
low 309 228 184 143 107 68 38
high 309 222 148 103 66 45 30
B
TWIST1
HR = 1.43 (1.11 − 1.84)
logrank P = 0.006
Probability
Time (months)
Expression
low
high
Number at risk
low 309 238 182 135 96 67 39
high 309 212 150 111 77 46 29
D
BNC2
HR = 0.64 (0.46 − 0.89)
logrank P = 0.0071
Probability
Time (months)
Expression
low
high
Number at risk
low 182 115 74 42 26 16 8
high 178 133 93 68 39 23 12
E
PRRX1
HR = 0.84 (0.61 − 1.15)
logrank P = 0.28
Probability
Time (months)
Expression
low
high
Number at risk
low 180 114 82 50 29 17 9
high 180 134 85 60 36 22 11
E
TBX5
HR = 0.98 (0.71 − 1.35)
logrank P = 0.88
Probability
Time (months)
Expression
low
high
Number at risk
low 180 120 79 52 32 20 9
high 180 128 88 58 33 19 11

Supplementary Figure S14: Survival Plots (RFS) depicting the relapse free survival in breast cancer of the **Basal** Subtype relation with the expression of: **A** - SNAI2, **B-** TWIST1, **C** – BNC2 , **D** – PRRX1 and TBX5.

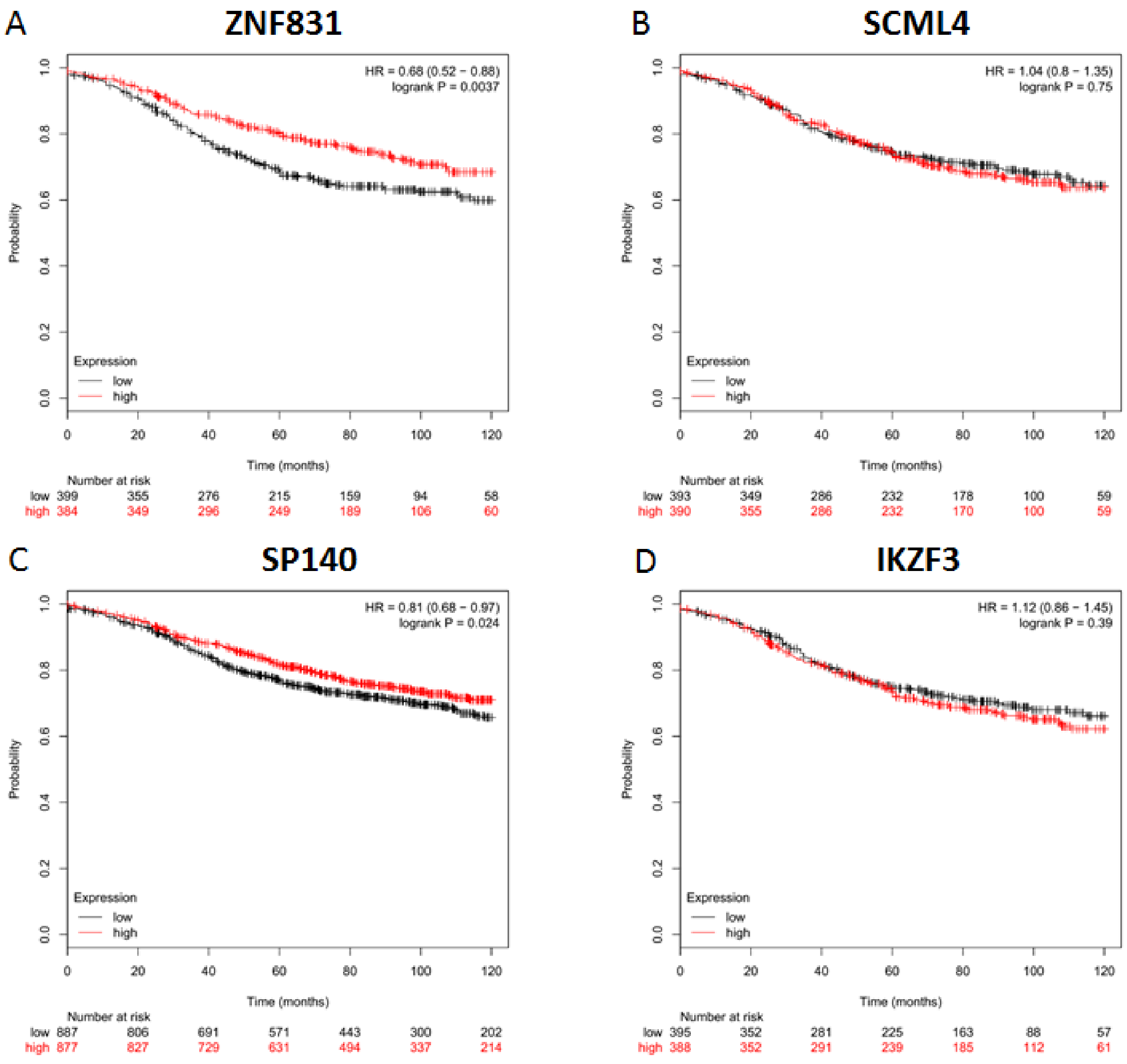

Supplementary Figure S15: Survival Plots (RFS) depicting the relapse free survival in breast cancer of the **Luminal A** Subtype relation with the expression of: **A** - ZNF831, **B**- SCML4, **C** - SP140 and **D** - IKZF3.

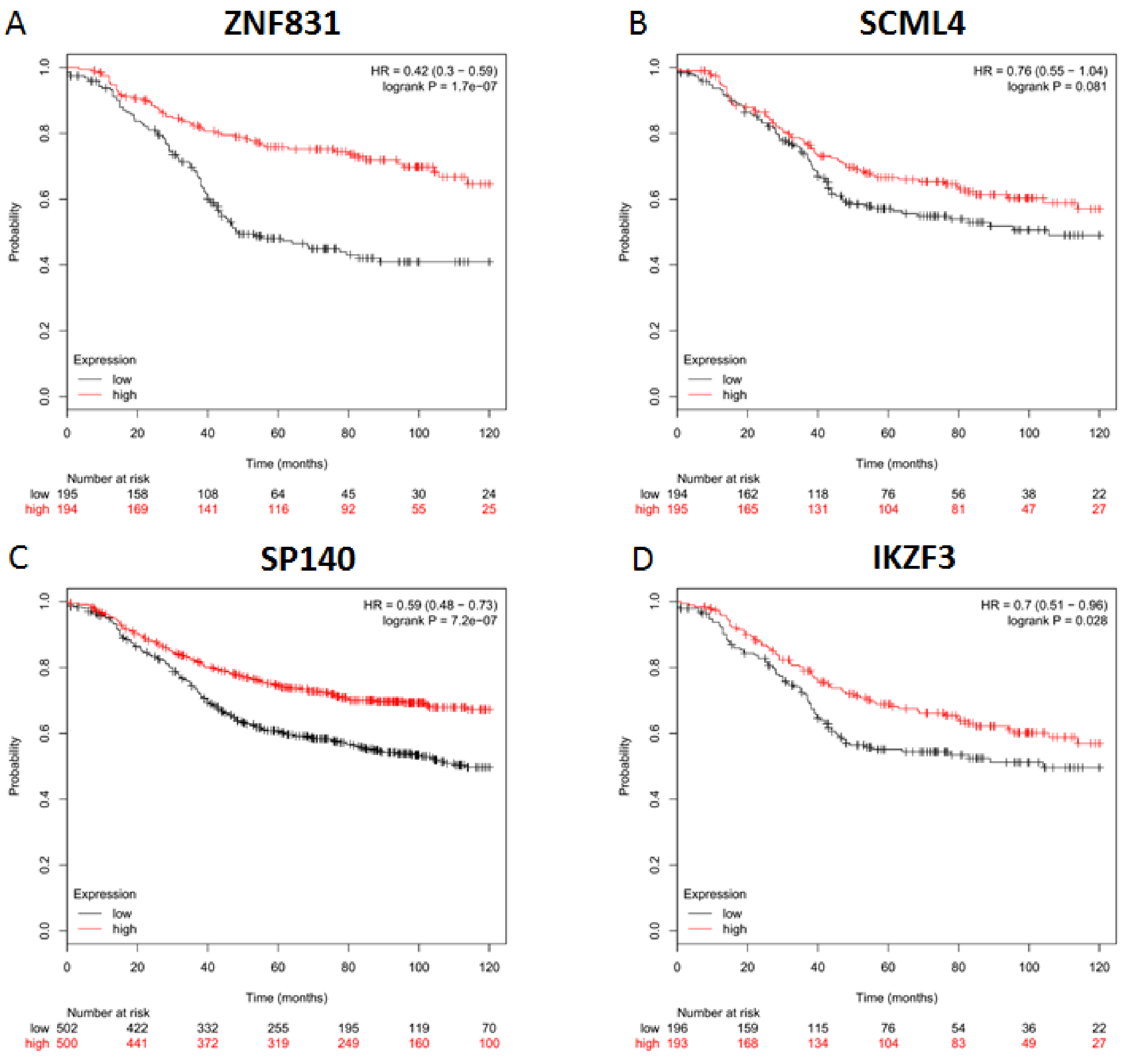

Supplementary Figure S16: Survival Plots (RFS) depicting the relapse free survival in breast cancer of the **Luminal B** Subtype relation with the expression of: **A** - ZNF831, **B**- SCML4, **C** - SP140 and **D** - IKZF3.

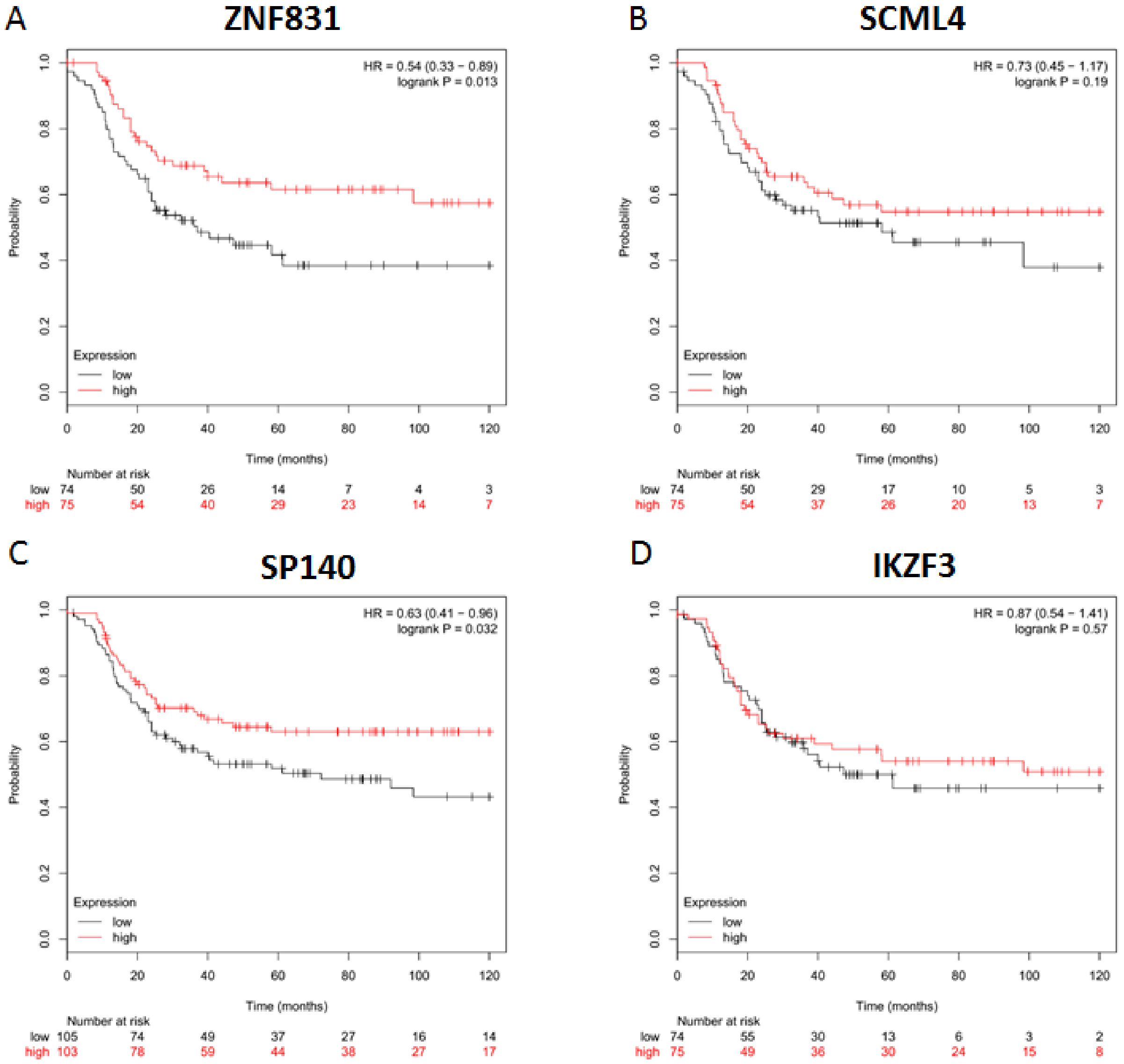

Supplementary Figure S17: Survival Plots (RFS) depicting the relapse free survival in breast cancer of the **HER2-Enriched** Subtype relation with the expression of: **A** - ZNF831, **B**- SCML4, **C** - SP140 and **D** - IKZF3.

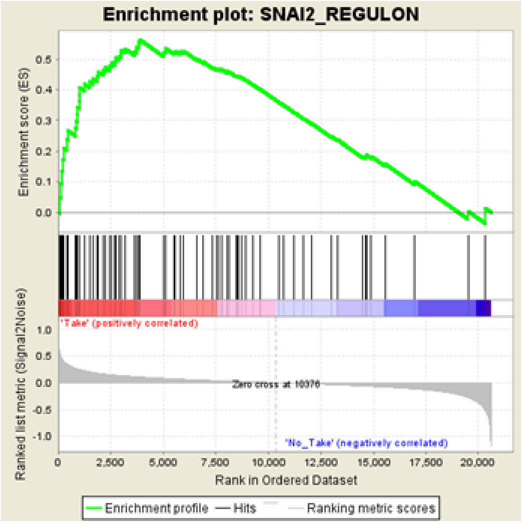
Enrichment plot: SNAI2_REGULON
Enrichment score (ES)
Ranked list metric (Signal2Noise)
'Take' (positively correlated)
Zero cross at 10376
'No_Take' (negatively correlated)
Rank in Ordered Dataset
Enrichment profile
Hits
Ranking metric scores

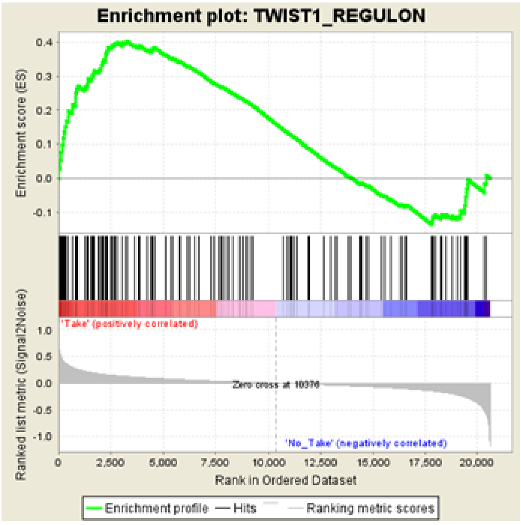
Enrichment plot: TWIST1_REGULON
Enrichment score (ES)
Ranked list metric (Signal2Noise)
'Take' (positively correlated)
Zero cross at 10376
'No_Take' (negatively correlated)
Rank in Ordered Dataset
Enrichment profile
Hits
Ranking metric scores

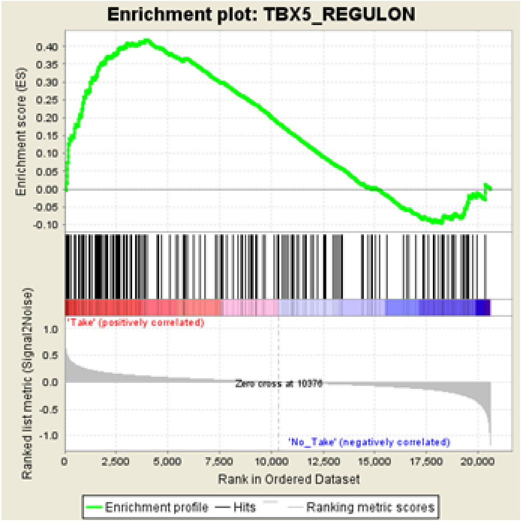
Enrichment plot: TBX5_REGULON
Enrichment score (ES)
Ranked list metric (Signal2Noise)
'Take' (positively correlated)
Zero cross at 10376
'No_Take' (negatively correlated)
Rank in Ordered Dataset
Enrichment profile
Hits
Ranking metric scores

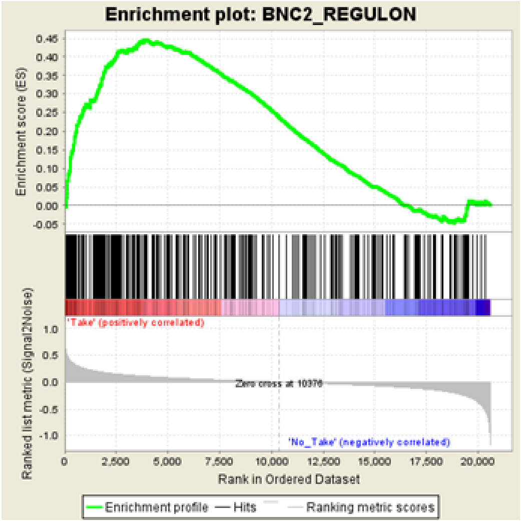
Enrichment plot: BNC2_REGULON
Enrichment score (ES)
Ranked list metric (Signal2Noise)
'Take' (positively correlated)
Zero cross at 10376
'No_Take' (negatively correlated)
Rank in Ordered Dataset
Enrichment profile
Hits
Ranking metric scores

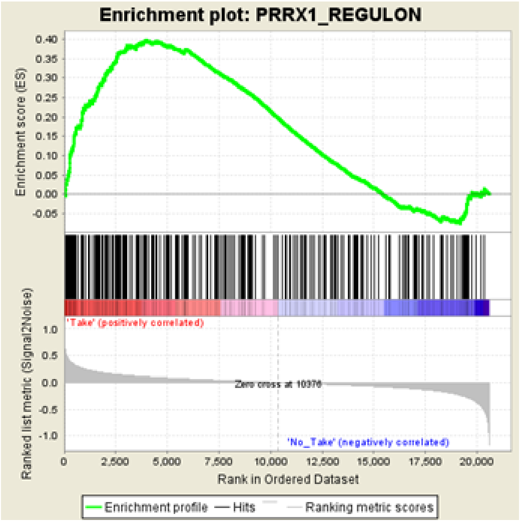
Enrichment plot: PRRX1_REGULON
Enrichment score (ES)
Ranked list metric (Signal2Noise)
'Take' (positively correlated)
Zero cross at 10376
'No_Take' (negatively correlated)
Rank in Ordered Dataset
Enrichment profile
Hits
Ranking metric scores

Supplementary Figure S18: GSEA analysis, Xenografts – Grown vs Engrafted. Positive correlation – regulons of the mesenchymal module transcription factors. Enrichment results of the positive correlation to Grown samples of GSEA analysis using the mesenchymal module transcription factor regulons in the xenograft dataset.

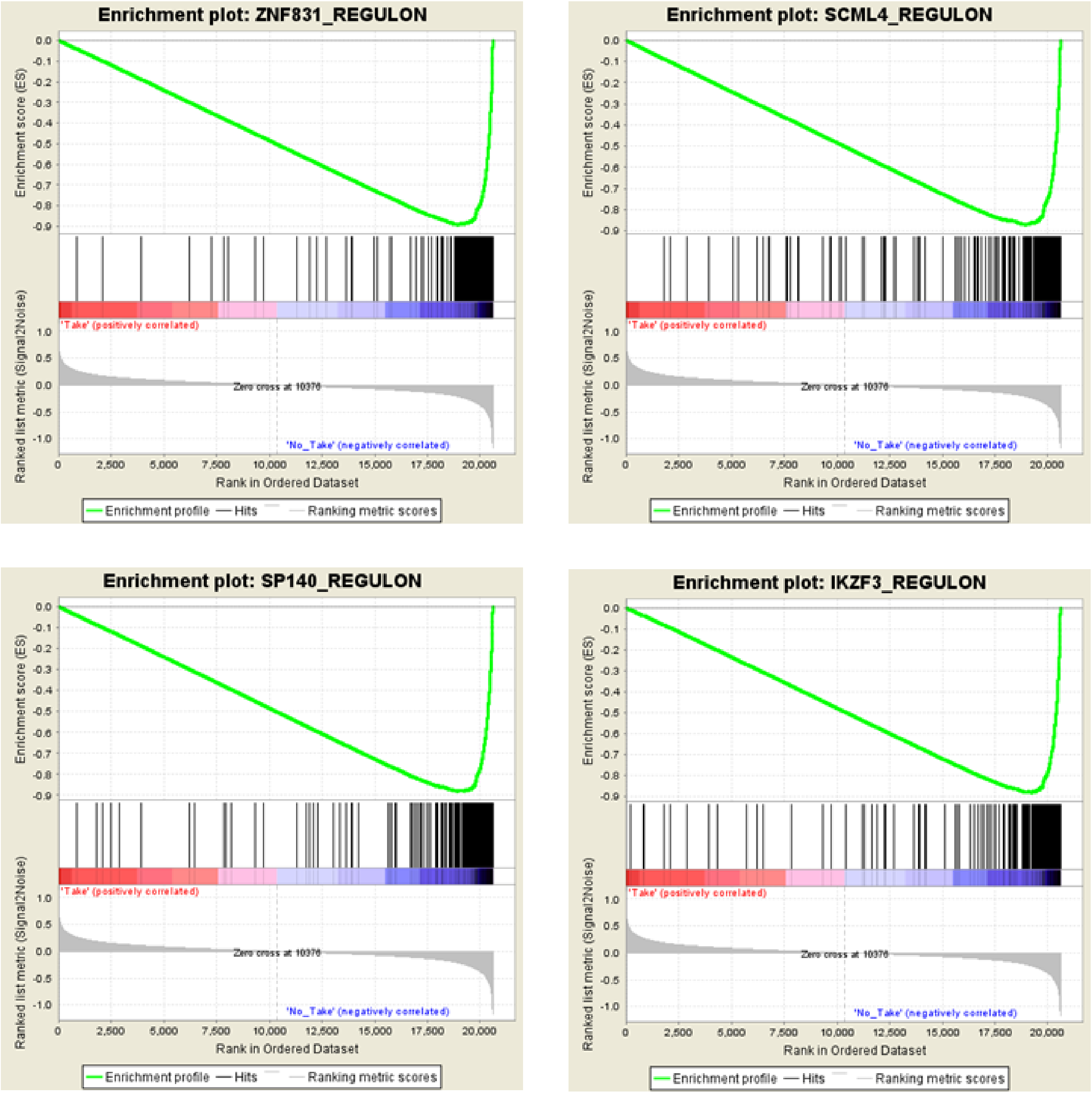

Supplementary Figure S19: GSEA analysis, Xenografts – Grown vs Engrafted. Negative correlation – regulons of the mesenchymal module transcription factors. Enrichment results of the negative correlation to Grown samples of GSEA analysis using the mesenchymal module transcription factor regulons in the xenograft dataset.

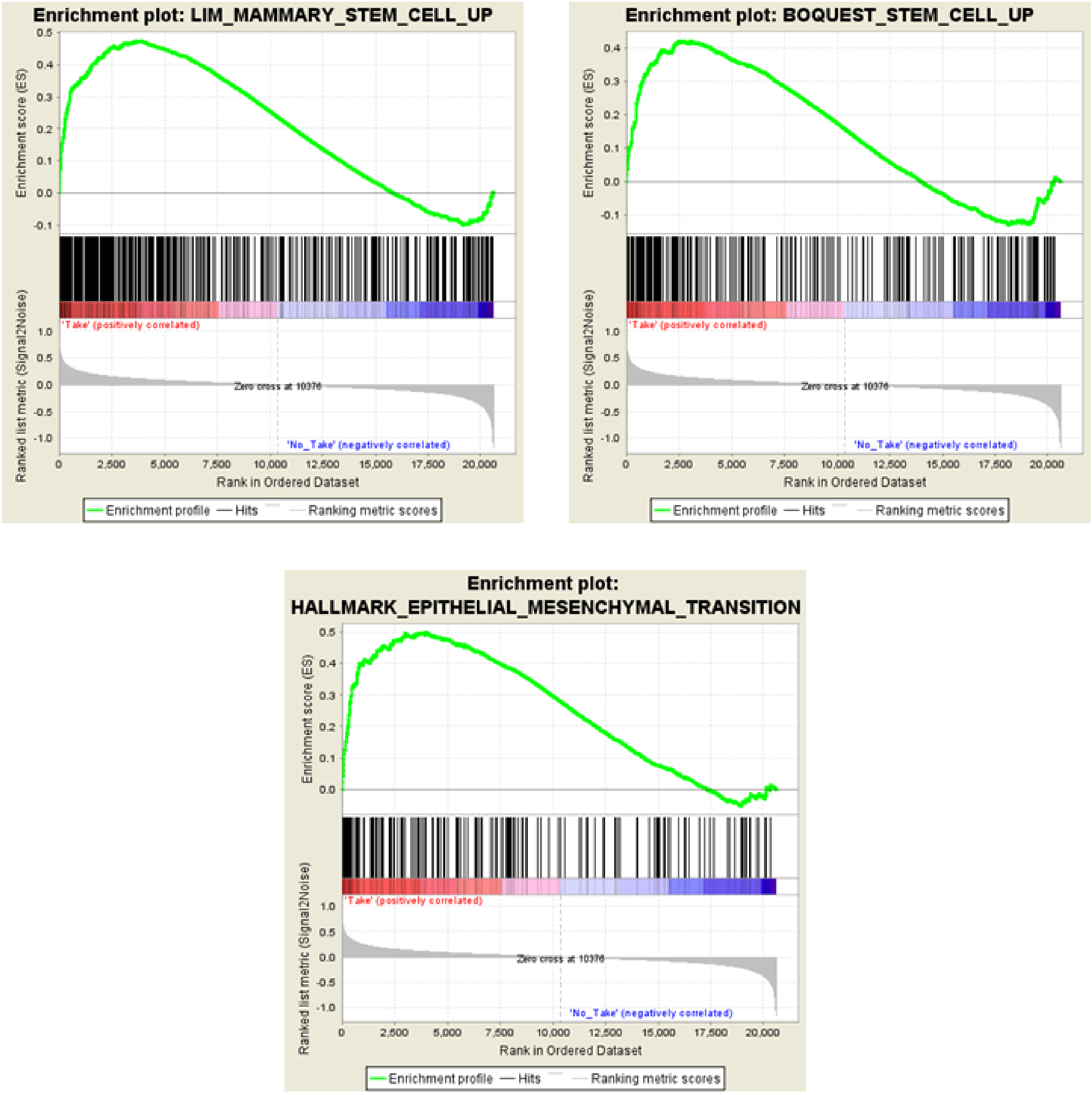

Supplementary Figure S20: GSEA analysis, Xenografts – Grown vs Engrafted. Positive correlation – Mesenchymal and Stem Cells genesets.

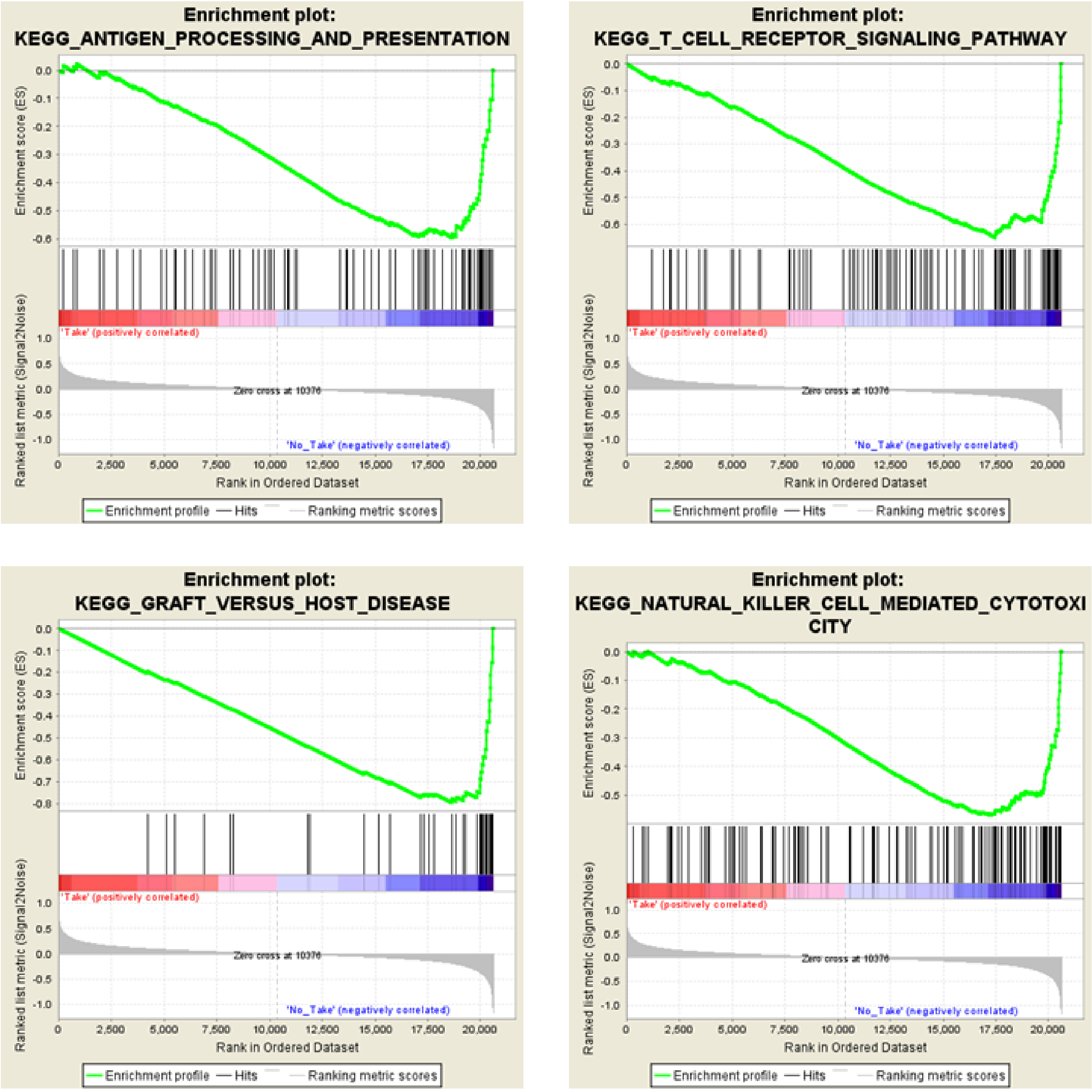

Supplementary Figure S21: GSEA analysis, Xenografts – Grown vs Engrafted. Negative correlation – KEGG Imunne genesets.

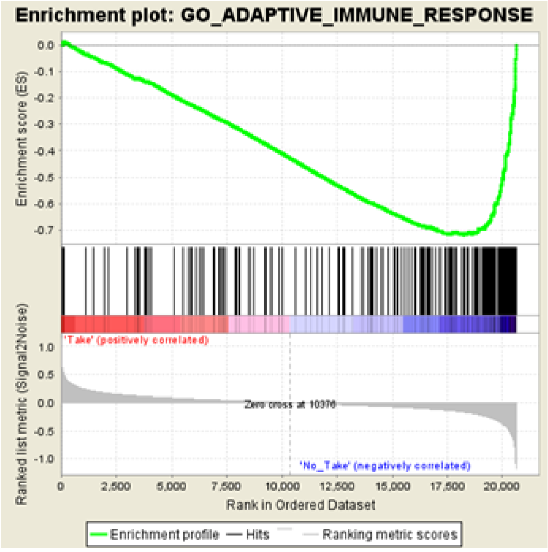
Enrichment plot: GO_ADAPTIVE_IMMUNE_RESPONSE
Enrichment score (ES)
Ranked list metric (Signal2Noise)
'Take' (positively correlated)
Zero cross at 10376
'No_Take' (negatively correlated)
Rank in Ordered Dataset
Enrichment profile
Hits
Ranking metric scores

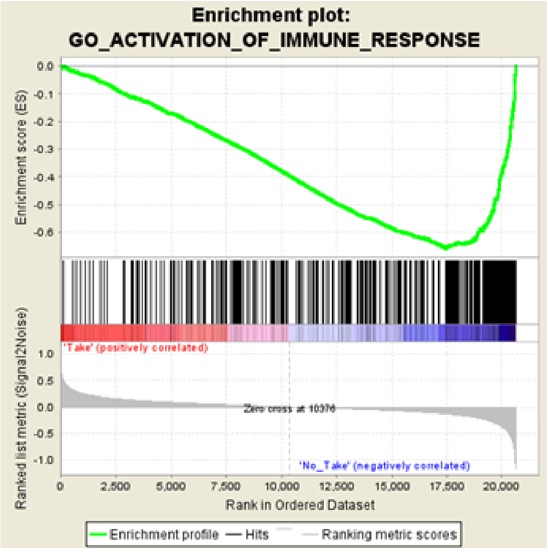
Enrichment plot: GO_ACTIVATION_OF_IMMUNE_RESPONSE
Enrichment score (ES)
Ranked list metric (Signal2Noise)
'Take' (positively correlated)
Zero cross at 10376
'No_Take' (negatively correlated)
Rank in Ordered Dataset
Enrichment profile
Hits
Ranking metric scores

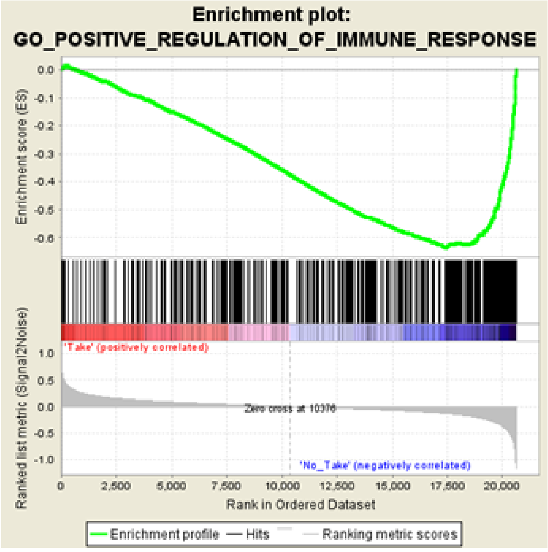
Enrichment plot: GO_POSITIVE_REGULATION_OF_IMMUNE_RESPONSE
Enrichment score (ES)
Ranked list metric (Signal2Noise)
'Take' (positively correlated)
Zero cross at 10376
'No_Take' (negatively correlated)
Rank in Ordered Dataset
Enrichment profile
Hits
Ranking metric scores

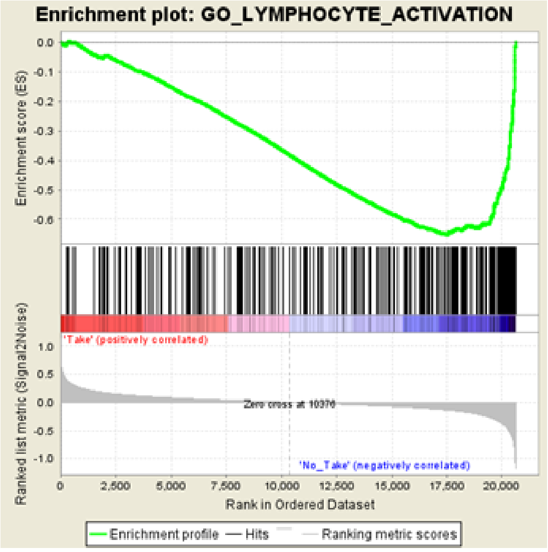
Enrichment plot: GO_LYMPHOCYTE_ACTIVATION
Enrichment score (ES)
Ranked list metric (Signal2Noise)
'Take' (positively correlated)
Zero cross at 10376
'No_Take' (negatively correlated)
Rank in Ordered Dataset
Enrichment profile
Hits
Ranking metric scores

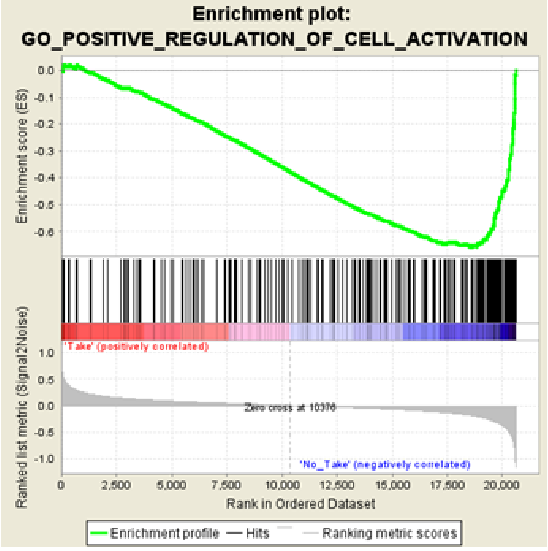
Enrichment plot: GO_POSITIVE_REGULATION_OF_CELL_ACTIVATION
Enrichment score (ES)
Ranked list metric (Signal2Noise)
'Take' (positively correlated)
Zero cross at 10376
'No_Take' (negatively correlated)
Rank in Ordered Dataset
Enrichment profile
Hits
Ranking metric scores

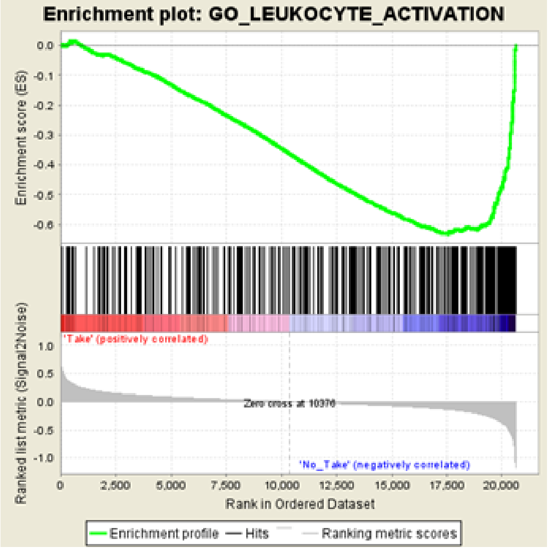
Enrichment plot: GO_LEUKOCYTE_ACTIVATION
Enrichment score (ES)
Ranked list metric (Signal2Noise)
'Take' (positively correlated)
Zero cross at 10376
'No_Take' (negatively correlated)
Rank in Ordered Dataset
Enrichment profile
Hits
Ranking metric scores

Supplementary Figure S22: Top 6 G.O terms. GSEA analysis, Xenografts – Grown vs Engrafted. Negative correlation.

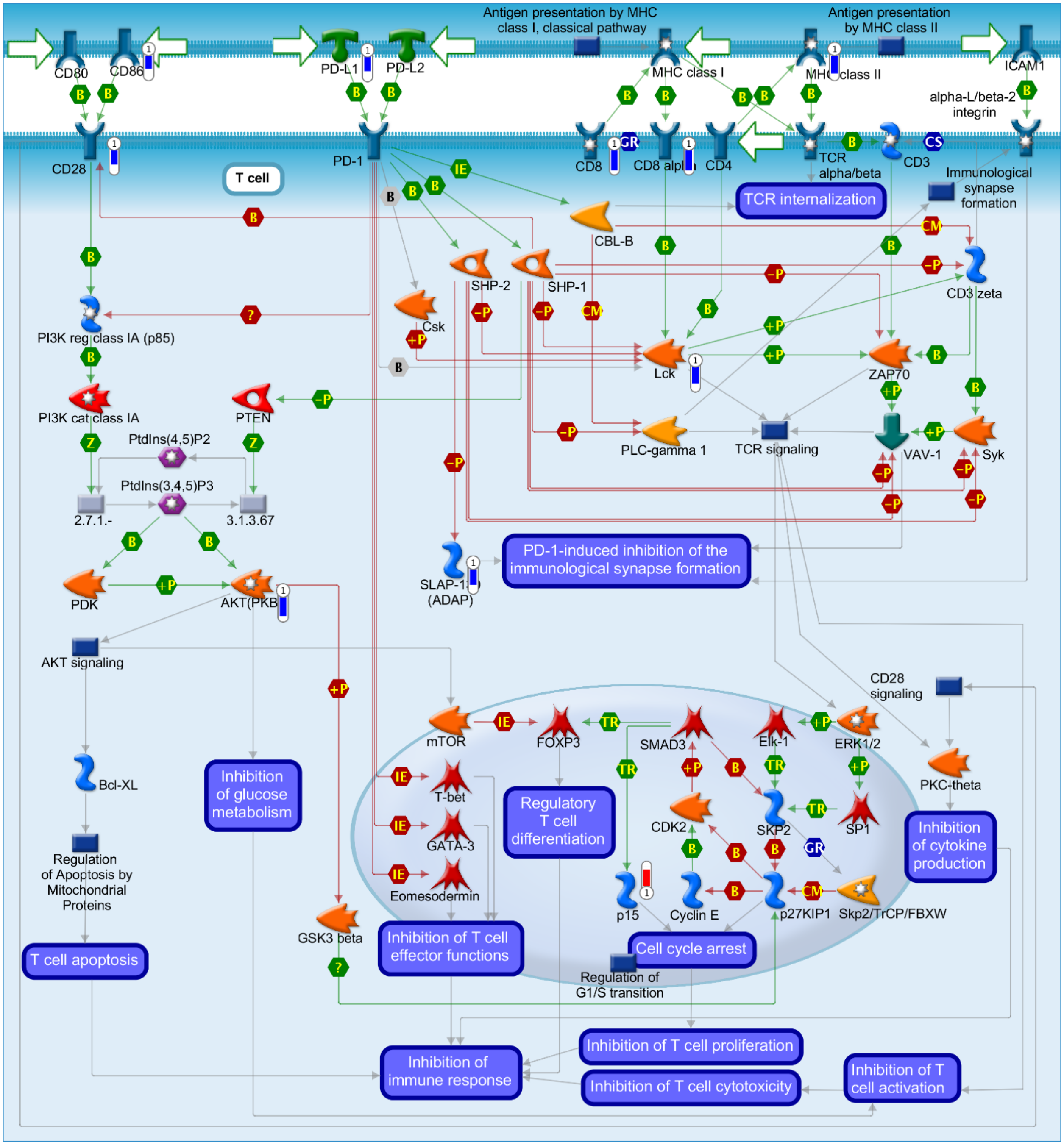

Supplementary Figure S23: METACORE analysis using transcriptome differences as input (Take vs No take human tumors with fold changes higher or lower than 1.5 log2). MHCII genes are downregulated with low levels of CD8 expression, what indicates the immune system did not recognize tumour new epitopes and, consequently, did not activate the adaptive immunity in Take samples. Blue bar represents decreased transcriptome expression in Take tumors and present in transcriptome input dataset.

**SNAI2_Regulon**	**TWIST1_Regulon**	**TBX5_Regulon**	**PRRX1_Regulon**	**BNC2_Regulon**
ADAM12	ABCA6	ABCC9	ABCC9	ABCA6
AGPAT4	ABCA8	ACACB	ACTA2	ABHD6
AKT3	ABCC9	ADAMTS7	ADAM12	ABI2
ANAPC7	ADCY4	ADRA2A	ADAMTS12	ADAM12
ANTXR1	ADRA2A	AKAP12	ADAMTS2	ADAMTS12
ARHGAP28	AKAP12	AKT3	ADAMTS5	ADAMTS2
BICC1	AKAP2	ANGPTL2	ADAMTS6	ADAMTS6
BNC2	AKT3	ANK2	ADAMTSL1	ADAMTS9.AS2
CALD1	ANGPTL2	ANTXR1	AEBP1	AEBP1
CDH11	ANK2	ANTXR2	AGPAT4	AKAP12
COL3A1	ANTXR2	ANXA1	AHNAK2	AKT3
COL6A3	APOB	ARHGAP20	AKT3	ALDH1L2
CRISPLD2	ARHGAP20	ARHGAP24	ALDH1L2	AMPH
CTSK	ATAD2	ASCL1	ANGPTL2	ANGPTL2
CYTH3	ATOH8	AXL	ANK2	ANK2
DACT1	AVPR1A	BHLHE41	ANTXR1	ANTXR1
DCN	BEND6	BIRC5	ANTXR2	ANTXR2
DDR2	BICC1	BMP1	ARHGAP24	ARHGAP20
DZIP1	BNC2	BNC2	ARHGAP28	ARHGAP24
EFEMP2	C13orf33	C1orf151-NBL1	ARHGAP31	ARHGAP28
EMP1	CCDC50	C1QTNF5	ARHGEF40	ARHGEF40
FBLN1	CCDC80	C3orf62	ASPN	ARL10
FBN1	CCL14	C5orf62	AXL	ASPN
FERMT2	CDC14B	CACNA2D1	BACE1	AXL
FEZ1	CDH5	CCDC25	BGN	BACE1
FIBIN	CEP112	CCDC36	BHLHE22	BEND6
FKBP7	CFH	CCDC80	BICC1	BGN
FKBP9	CLEC1A	CDH11	BMP1	BHLHE22
FSTL1	CNRIP1	CDK13	BMPR2	BICC1
GAS1	COLEC12	CDR1	BNC2	BMP1
GAS7	CPED1	CFH	C13orf33	BNC2.1
GJA1	CPXM1	CHL1	C14orf37	C14orf37
GLIS3	CXCL12	CKAP2	C1orf151-NBL1	C1orf135
GLT8D2	DAB2	CLMP	C1QTNF5	C1orf151.NBL1
GOLIM4	DCN	CLU	C1S	C1QTNF5
GXYLT2	DDR2	CNRIP1	C5orf62	C20orf194
HIST1H2BD	DLC1	COL5A3	C9orf3	C5orf62

HMCN1	DLG3	COLEC12	CALD1	CACNA2D1
HOXA10-HOXA9	DST	COPZ2	CAV2	CALD1
HTRA1	EBF1	CTSK	CCDC69	CCDC8
KDELC1	EBF3	CWF19L2	CCDC80	CCDC80
LAMB1	ECSCR	CXCL12	CD109	CD99
LAMC1	EFEMP1	DAAM2	CD248	CDCA5
LOC100132116	EFHA2	DAB2	CD99	CDH11
LOC100505813	EGFLAM	DAD1	CDH11	CDH5
LOX	EHD2	DCHS1	CDR1	CDK6
LUM	ENPEP	DCN	CELF2	CFH
MEG3	EPDR1	DDR2	CFH	CHRNA1
MEG8	ERG	DFFB	CFI	CHSY3
MIR100HG	ESAM	DLC1	CHSY3	CLMP
MSRB3	ESRP1	DNM3OS	CLDN5	CMTM3
MXRA7	ESYT1	DPT	CLMP	CNRIP1
NAP1L3	EVC2	DPYSL2	CMTM3	CNTN1
NID1	F13A1	DPYSL3	CNRIP1	COL10A1
NID2	FAM13A-AS1	DSEL	CNTN1	COL12A1
NT5E	FAM162B	DST	COL10A1	COL14A1
OLFML2A	FBLN1	EFEMP2	COL12A1	COL16A1
PAM	FBLN2	EFHA2	COL1A1	COL18A1
PARVA	FBN1	EHD2	COL1A2	COL1A1
PCDH7	FERMT2	EMP1	COL3A1	COL1A2
PDGFRL	FEZ1	EMX2	COL5A1	COL3A1
PLS3	FGF7	ERG	COL5A2	COL5A1
POSTN	FLRT2	ESRP1	COL6A1	COL5A2
PRKD1	FOXN3	ESYT2	COL6A2	COL6A1
PRRX1	FOXO1	F13A1	COL6A3	COL6A2
PTPRG	FSTL1	FAM41AY1	COL8A1	COL6A3
QKI	GALNTL2	FAM82A1	COL8A2	COL8A1
RECK	GAS1	FAP	COPZ2	COL8A2
RFTN2	GNG11	FAT4	CPXM1	COPZ2
SACS	GPR116	FBLN1	CPZ	CORIN
SEPT11	GPR124	FBN1	CRISPLD2	CPXM1
SFRP2	GPR146	FBXL5	CRTAP	CPZ
SGCD	HDAC9	FETUB	CSRNP3	CRTAP
SH3PXD2A	HS2ST1	FEZ1	CSRP2	CSTF3
SH3PXD2B	HSPB6	FFAR3	CTSK	CTDSP2
SHOX2	IGF2	FGF10	CXCL12	CTHRC1
SNX18	IPO11	FHL1	CXorf36	CTSK
SPARCL1	ITGA1	FIBIN	CYP2U1	CTTNBP2

SPON1	KIAA1462	FMOD	CYS1	CXCL12
SRPX	KLHL3	FOXN3	CYTH3	CYBRD1
SSPN	LAMA4	FOXO1	DAB2	CYGB
TCF4	LDB2	FSTL1	DACT1	CYS1
THBS2	LEPR	GALNTL2	DCN	DAB2
TIMP2	LEPROT	GAS1	DDR2	DACT1
TRPC1	LHFP	GAS7	DLC1	DCHS1
TSHZ3	LOC100505679	GJC1	DNM3OS	DCN
WWTR1	LRP1	GOLGA8IP	DPT	DDR2
XYLT1	MAF	GPR124	DPYSL3	DKK3
ZEB1	MAGI2-AS3	HIC1	DST	DLC1
ZNF521	MAP1B	HIST1H2BC	EDNRA	DNM3OS
	MAP4	HIST1H2BH	EFEMP2	DPT
	MAP4K4	HMCN1	EHD2	DPYSL3
	MCAM	HNMT	ELK3	DSEL
	MECOM	HOXA10-HOXA9	EMCN	DST
	MEF2C	HOXA7	EMILIN2	DZIP1
	MEG3	HSPA12B	EMP1	EBF1
	MIR100HG	IFFO1	ERG	EDNRA
	MME	IGFBP4	ESAM	EFEMP2
	MMRN2	ISLR	EVC	EFHA2
	MRVI1	ITGBL1	F13A1	EHD2
	MSRB3	JAM2	FAM26E	EIF5A2
	MXRA7	JAM3	FAP	EMX2
	MYCT1	KANK2	FBLN1	ENTPD1
	NDN	KCTD12	FBLN2	EVC
	NLRP1	KIAA1462	FBN1	EVC2
	NR3C1	KIRREL	FERMT2	FABP4
	NRP1	KRR1	FEZ1	FAM198B
	NT5E	KRT84	FGD5	FAM26E
	OLFML1	LAMA4	FHL1	FANCA
	PARVA	LAMB1	FIBIN	FAP
	PCDH18	LDB2	FILIP1L	FAS
	PCSK5	LHFP	FLRT2	FAT3
	PDE3A	LIMA1	FMOD	FBLN1
	PDGFRA	LOC100505813	FN1	FBLN2
	PEAK1	LOC100507630	FOXN3	FBN1
	PECAM1	LPAR1	FOXO1	FER
	PELI2	LRP1	FSTL1	FERMT2
	PGM5	LUM	GAS1	FEZ1
	PLAGL1	MAF	GAS7	FGD5

	PRDM5	MAGI2-AS3	GFPT2	FGF1
	PRRX1	MAP1A	GLT8D2	FGF14
	PTPRB	MAP4K4	GNG2	FGF18
	PTPRG	MEG3	GOLGA8IP	FHL1
	PTRF	MEG8	GPR124	FHL5
	RARRES2	MEIS2	GXYLT2	FIBIN
	RBMS2	MIR10A	HEG1	FILIP1L
	RECK	MIR143HG	HMCN1	FKBP7
	RHOJ	MLL3	HNMT	FLRT2
	ROBO4	MMP19	HOTAIRM1	FMOD
	RUNX1T1	MRVI1	HOXA10-HOXA9	FN1
	S1PR1	MSRB3	HRH1	FRMD6
	SEMA6A	NAP1L3	HSD17B11	FSTL1
	SEPT11	NAV3	HSPG2	GAS1
	SERPING1	NDN	HTRA1	GAS7
	SH3RF3	NEGR1	IFFO1	GFPT2
	SLC16A7	NID1	IGF2	GJB2
	SLIT3	NID2	IGFBP7	GLIS3
	SOD3	NNMT	IL1R1	GLT8D2
	SOX7	NPR2	ISLR	GPC6
	SRPX	NRP2	ITGA1	GPX8
	SSPN	NUAK1	ITGBL1	GXYLT2
	SULT1C4	OLFML1	JAM2	H2AFZ
	SVEP1	OLFML3	JAM3	HEG1
	SYNE1	PALM	KANK2	HEPH
	TCEAL7	PARP1	KCTD12	HGF
	TCF4	PARVA	KIAA1462	HMCN1
	TEK	PCSK5	KIRREL	HRH1
	TENC1	PDE11A	LAMA4	HSD17B12
	TFPI	PDE3A	LAMB1	HSPA12B
	TGFBR2	PDGFRB	LAMC1	HTRA1
	THSD7A	PDGFRL	LATS2	HTRA3
	TIMP2	PEAK1	LDB2	IGDCC4
	TRPC1	PELI2	LEPROT	IGF2
	TSHZ3	PLSCR4	LHFP	IGFBP4
	TSPAN7	PNMA6A	LINC00478	IL1R1
	TWIST2	PRDM2	LINC00514	ISLR
	VIM	PRICKLE2	LIX1L	ITGA1
	WHAMMP2	PRKD1	LOC100132116	ITGB5
	ZEB1	PRPF4B	LOC100132891	ITGBL1
	ZEB2	PRRX1	LOC100505813	JAM2

	ZFHX4	PTGIS	LOC100506941	JAM3
	ZNF471	PTPRG	LOC100653217	JAZF1
		PTPRM	LOC728392	KANK2
		PTRF	LOX	KCND2
		PURG	LOXL1	KCNJ12
		QKI	LPAR1	KCNMB4
		RAP1A	LRCH2	KIAA1199
		RARRES2	LRP1	KIAA1462
		RASGRF2	LRRC15	KIF26B
		RBMS2	LRRC32	KLHL3
		RCAN2	LUM	LAMA2
		RCBTB2	MAF	LAMA4
		RECK	MAGEL2	LAMB1
		RFTN2	MAGI2-AS3	LATS2
		RNF180	MAN1A1	LDB2
		RSPO3	MAN2A1	LEPREL1
		RUNX1T1	MAP1A	LHFP
		RUNX2	MAP4K4	LMO1
		SEPT11	MBNL1	LMOD1
		SERPINF1	MCAM	LOC100130872
		SFRP4	MEG3	LOC100132891
		SGCD	MEG8	LOC100287221
		SHOX2	MEIS2	LOC100505813
		SLIT2	MFAP4	LOC100506941
		SLIT3	MFAP5	LOC100509635
		SMC4	MGC24103	LOC283867
		SNCA	MIR100HG	LOX
		SORBS1	MIR143HG	LOXL1
		SPARCL1	MME	LOXL2
		SPATA9	MMP2	LPAR1
		SPON1	MN1	LRCH1
		SRSF2	MNF1	LRCH2
		SSPN	MRGPRF	LRP1
		STON1	MRVI1	LRRC15
		SVEP1	MSRB3	LRRC17
		SYNE1	MTMR7	LRRC32
		TBC1D2B	MXRA5	LSAMP
		TBX15	MXRA7	LTBP2
		TBX5	MXRA8	LUM
		TBX5-AS1	MYCT1	MAGEL2
		TCF4	MYL9	MAGI2.AS3

		TEK	NAP1L3	MAN1A1
		TGFBR2	NBLA00301	MAN2A1
		TIMP2	NDN	MAP4K4
		TLN2	NEGR1	MED17
		TNS1	NHS	MEG3
		TRPC1	NID1	MEG8
		TSHZ3	NID2	MFAP4
		TWIST2	NNMT	MFAP5
		UBE3C	NOX4	MGC24103
		USP1	NRN1	MGC4294
		VCAN	NRP1	MIR100HG
		VGLL3	NRP2	MIR143HG
		VSTM4	NT5E	MMP19
		ZEB1	NUAK1	MMP2
		ZFHX4	ODZ4	MN1
		ZFHX4-AS1	OLFML1	MRC2
		ZFPM2	OLFML2B	MRGPRF
		ZMAT3	OLFML3	MRVI1
		ZNF423	OSBP2	MSRB3
		ZNF697	PALLD	MTHFD2
			PAM	MTMR2
			PARVA	MXRA5
			PCDH7	MXRA7
			PCOLCE	MXRA8
			PCSK5	MYH2
			PDE3A	MYL6
			PDE7B	MYL9
			PDGFC	MYLK
			PDGFRA	NAP1L3
			PDGFRB	NAV1
			PDGFRL	NAV3
			PDLIM2	NBL1
			PDPN	NBLA00301
			PEAK1	NDN
			PECAM1	NDNF
			PLAGL1	NEGR1
			PLXDC1	NID1
			PLXDC2	NID2
			POSTN	NNMT
			PPAP2B	NOVA1
			PPAPDC1A	NOX4

			PRDM5	NPR2
			PRICKLE1	NRP2
			PRICKLE2	NT5E
			PRKD1	NUAK1
			PRKG1	ODZ4
			PRNP	OLFML1
			PRRX1	OLFML2B
			PRTFDC1	OLFML3
			PTPN21	P4HA3
			PTPRD	PALLD
			PTPRG	PAM
			PTRF	PARVA
			RAB23	PCDH7
			RARRES2	PCSK5
			RASAL2	PDE1A
			RASGRF2	PDE3A
			RBMS2	PDE4DIP
			RBMS3	PDGFC
			RECK	PDGFRA
			RFTN2	PDGFRB
			RHOJ	PDGFRL
			RNF144A	PDLIM2
			RNF146	PDPN
			ROR1	PELI2
			RRAGA	PELO
			RUNX1T1	PHLDB1
			RUNX2	PLXDC2
			S100A6	PLXNC1
			SAMD4A	POSTN
			SEPT11	PPAPDC1A
			SERPINF1	PRDM5
			SFRP2	PRDM6
			SGCD	PRELP
			SH3PXD2A	PRICKLE1
			SH3RF3	PRICKLE2
			SHOX2	PRKD1
			SLC4A4	PRKG1
			SLIT3	PRND
			SNAI2	PROS1
			SNED1	PRRX1
			SNORD114-3	PTGER3

			SNX18	PTPN21
			SNX29	PTPRD
			SPARCL1	PTPRG
			SPOCK1	PTRF
			SPON1	PURG
			SRPX	RAI2
			SSC5D	RARRES2
			SSPN	RASGRF2
			STAB1	RBMS2
			STGC3	RBMS3
			STON1	RCAN2
			SYNE1	RECK
			SYT11	RFTN2
			TAGLN	RNF144A
			TBC1D2B	RORB
			TBX18	RPS6KA2
			TBX5	RRAGA
			TBX5-AS1	RUNX1T1
			TCEAL7	RUNX2
			TCF12	SALL1
			TCF4	SATB2
			TENC1	SEC23A
			TGFBR2	Sep.11
			THBS2	SERPINE1
			THSD7A	SERPINF1
			THY1	SFRP2
			TIE1	SFRP4
			TIMP2	SGCD
			TLL1	SH3D19
			TMEM200A	SH3PXD2A
			TMEM204	SHOX2
			TMEM37	SLIT2
			TMEM47	SLIT2.IT1
			TNS1	SLIT3
			TPM2	SNED1
			TPST1	SNX29
			TRPC1	SPARCL1
			TSEN54	SPATS2L
			TSHZ3	SPOCK1
			TSPAN18	SPON1
			TUBB6	SRPX

			TWIST2	SRPX2
			VCAN	SRXN1
			VGLL3	SSC5D
			VIM	ST6GAL2
			VSTM4	STON1
			WISP1	SULF1
			WWTR1	SYNE1
			ZCCHC24	SYNPO
			ZEB1	SYT11
			ZEB2	TAGLN
			ZFHX4	TBX15
			ZFHX4-AS1	TBX18
			ZFPM2	TBX2
			ZNF423	TBX5
			ZNF521	TBX5.AS1
			ZNF781	TCF4
				TGFB1I1
				THBS2
				THSD7A
				THY1
				TIMP2
				TMEM200A
				TMEM204
				TNS1
				TP53I3
				TPM2
				TRO
				TRPC1
				TSHZ3
				UBE2QL1
				VCAN
				VIM
				VSTM4
				WISP1
				ZCCHC24
				ZEB1
				ZFHX4
				ZFHX4.AS1
				ZFPM2
				ZNF385D
				ZNF423

				ZNF521
				ZNF781

IKZF3_Regulon	SP140_Regulon	SCML4_Regulon	ZNF831_Regulon
ABI3	ACAP1	ACAP1	ACAP1
ACAP1	ACSL5	ADAM28	ADCY7
ADAMDEC1	ADAMDEC1	AIM2	AIM2
AIM2	ADCY7	AKNA	AKNA
AKNA	AIM2	AMICA1	AMICA1
ANKRD36BP2	AKNA	ANKRD44	ANKRD44
ANKRD44	ANKRD44	AOAH	AOAH
AOAH	ANKRD55	APOBEC3G	APOBEC3G
APOBEC3F	AOAH	ARHGAP15	ARHGAP15
APOBEC3G	APBB1IP	ARHGAP25	ARHGAP25
ARHGAP15	APOBEC3G	ARHGAP27	ARHGAP30
ARHGAP25	APOL3	ARHGAP30	ARHGAP9
ARHGAP30	APOL6	ARHGAP9	B2M
ARHGAP9	ARHGAP15	ATM	BANK1
B2M	ARHGAP25	ATP8A1	BCL11B
BANK1	ARHGAP30	B2M	BIN2
BCL11B	ARHGAP9	BANK1	BIRC3
BCL2A1	ATM	BCL11B	BTLA
BIN2	B2M	BCL2A1	BTN3A1
BIRC3	BANK1	BEND4	BTN3A2
BMP6	BCL11B	BIN2	BTN3A3
BTK	BCL2A1	BIRC3	BZRAP1-AS1
BTLA	BEND4	BTLA	C11orf21
BTN3A1	BIN2	BTN2A2	C16orf54
BTN3A3	BIRC3	BTN3A2	C1orf186
BZRAP1-AS1	BTK	BTN3A3	C5orf58
C11orf21	BTLA	C11orf21	CAMK4
C16orf54	BTN2A2	C16orf54	CCDC69
C1orf186	BTN3A1	C16orf62	**CCL19**
C5orf20	BTN3A2	C5orf20	**CCL5**
C5orf58	BTN3A3	C5orf58	CCR2
CAMK4	BZRAP1-AS1	C7orf44	CCR5
CARD11	C11orf21	CAMK4	CD19
CCDC88B	C16orf54	CARD11	CD2
**CCL19**	C1orf162	CARD8	CD200R1
CCL4	C1orf186	CASP1	CD226
**CCL5**	C5orf20	CASP10	CD247

CCR2	C5orf58	CCDC69	CD27
CCR4	CAMK4	CCDC88B	CD28
CCR5	CARD11	**CCL19**	CD38
CCR7	CARD16	**CCL5**	CD3D
CD19	CASP1	CCR2	CD3E
CD2	CASP10	CCR4	CD3G
CD200R1	CCL4	CCR5	CD40
CD226	**CCL5**	CCR7	CD40LG
CD247	CCR2	CD19	CD48
CD27	CCR4	CD1D	CD5
CD274	CCR5	CD2	CD52
CD28	CCR7	CD200R1	CD53
CD37	CCRL2	CD226	CD6
CD38	CD19	CD244	CD69
CD3D	CD2	CD247	CD74
CD3E	CD226	CD27	CD84
CD3G	CD247	CD274	CD8A
CD48	CD274	CD28	CD8B
CD5	CD28	CD38	CD96
CD52	CD38	CD3D	CDC42SE2
CD53	CD3D	CD3E	CEACAM21
CD6	CD3E	CD3G	CELF2
CD69	CD3G	CD40LG	CIITA
CD72	CD40	CD48	CKAP2
CD74	CD48	CD5	CLEC2D
CD79A	CD5	CD52	CLECL1
CD79B	CD52	CD53	CLUAP1
CD80	CD53	CD6	CORO1A
CD84	CD6	CD69	COTL1
CD8A	CD69	CD72	CR1
CD8B	CD72	CD79A	CR2
CD96	CD79A	CD79B	CRTAM
CDC42SE2	CD79B	CD84	CSF2RB
CDON	CD80	CD8A	CST7
CEACAM21	CD83	CD8B	CTLA4
CELF2	CD84	CD96	CTSW
CFLAR	CD86	CDC42SE2	**CXCL9**
CIITA	CD8A	CEACAM21	CXCR3
CKAP2	CD8B	**CECR1**	CXCR5
CLEC2D	CD96	CELF2	CXCR6
CLECL1	CDC42SE2	CIITA	CXorf65

CLUAP1	CEACAM21	CLEC2D	CYAT1
CORO1A	CEP128	CLEC4C	CYLD
CPNE5	CIITA	CLECL1	CYTH4
CR1	CKAP2	CLLU1	CYTIP
CR2	CLEC2D	CLNK	DENND1C
CRTAM	CLECL1	CLUAP1	DERL3
CSF2RB	CLLU1	CNTRL	DGKA
CST7	CLUAP1	COL4A4	DOCK2
CTLA4	CMKLR1	CORO1A	DOCK8
CTSC	CORO1A	COTL1	DTHD1
CTSW	COTL1	CR1	EOMES
**CXCL10**	CR1	CRTAM	EVI2B
**CXCL11**	CR2	CSF2RB	FAM26F
**CXCL13**	CRTAM	CST7	FAM65B
**CXCL9**	CSF2RB	CTLA4	FASLG
CXCR3	CTLA4	CTSC	FCRL1
CXCR4	CTSC	CTSS	FCRL2
CXCR5	CTSS	CTSW	FCRL3
CXCR6	**CXCL10**	CUX2	FCRL5
CXorf65	**CXCL11**	**CXCL13**	FCRLA
CYAT1	**CXCL13**	**CXCL9**	FGD2
CYLD	**CXCL9**	CXCR3	FGL2
CYTIP	CXCR3	CXCR4	FLJ32255
DAPP1	CXCR5	CXCR5	FNBP1
DEF6	CXCR6	CXCR6	FYB
DENND1C	CXorf21	CXorf65	GBP1
DOCK2	CXorf65	CYTH4	GBP2
DOCK8	CYBB	CYTIP	GBP4
DTHD1	CYLD	DEF6	GBP5
EAF2	CYTH4	DOCK10	GFI1
EML4	CYTIP	DOCK2	GIMAP1-GIMAP5
EOMES	DAPP1	DOCK8	GIMAP6
EVI2B	DDX26B	DPEP2	GIMAP7
FAM129C	DENND1C	DTHD1	GLYR1
FAM26F	DOCK10	EML4	GNLY
FAM46C	DOCK2	EOMES	GPR174
FAM65B	DOCK8	EPSTI1	GPR183
FAM78A	EML4	EVI2B	GPR65
FASLG	EOMES	FAIM3	GVINP1
FCRL1	EPHA1-AS1	FAM129C	GZMA
FCRL2	EPSTI1	FAM26F	GZMB

FCRL3	ETS1	FAM65B	GZMH
FCRL5	EVI2B	FAM71B	GZMK
FCRLA	F5	FAM78A	HCLS1
FGD2	FAM26F	FASLG	HLA-DOA
FGL2	FAM65B	FCRL1	HLA-DOB
FLJ32255	FAM78A	FCRL2	HLA-DPA1
FMNL1	FASLG	FCRL3	HLA-DPB1
FNBP1	FCHSD2	FCRL5	HLA-DQA1
FYB	FCRL1	FCRLA	HLA-DQB1
GABBR1	FCRL2	FERMT3	HLA-DRA
GBP1	FCRL3	FGD2	HLA-DRB1
GBP2	FCRL5	FGL2	HLA-E
GBP4	FCRLA	FMNL1	HLA-F
GBP5	FGD2	FNBP1	HMHA1
GFI1	FGL2	FYB	ICOS
GIMAP1-GIMAP5	FLI1	FYN	IDO2
GLYR1	FMNL1	GABBR1	**IFNG**
GNLY	FNBP1	GBP4	IGFLR1
GP1BA	FYB	GBP5	IGHA1
GPR171	GAB3	GFI1	IGHG1
GPR174	GABBR1	GIMAP1	IGHM
GPR52	GBP1	GIMAP1-GIMAP5	IGK@
GRAP2	GBP2	GIMAP4	IGKC
GVINP1	GBP4	GIMAP6	IGLC1
GZMA	GBP5	GIMAP7	IGLV1-44
GZMB	GFI1	GIMAP8	IKZF1
GZMK	GIMAP1	GLYR1	IKZF3
HCLS1	GIMAP1-GIMAP5	GMFG	IL10RA
HLA-DMB	GIMAP2	GNLY	IL12RB1
HLA-DOA	GIMAP4	GP1BA	IL16
HLA-DOB	GIMAP6	GPR171	IL18R1
HLA-DPA1	GIMAP7	GPR174	IL18RAP
HLA-DPB1	GLS	GPR18	IL21R
HLA-DQA1	GMFG	GPSM3	IL23A
HLA-DQB1	GNLY	GRAP2	IL26
HLA-DRA	GPR171	GVINP1	IL2RB
HLA-F	GPR174	GZMA	IL2RG
HMHA1	GPR183	GZMK	IL7R
ICOS	GPR65	HCLS1	INPP4A
IDO1	GRAP2	HIGD1A	INPP5D

IDO2	GVINP1	HLA-DMB	IPCEF1
**IFNG**	GZMA	HLA-DOA	IRF1
IGFLR1	GZMB	HLA-DOB	IRF4
IGH@	GZMH	HLA-DPB1	IRF8
IGHA1	GZMK	HLA-DRA	ITGA4
IGHD	HCLS1	HLA-E	ITGAL
IGHG1	HLA-A	HLA-F	ITGB7
IGHM	HLA-C	HMHA1	ITK
IGHV3-48	HLA-DMB	HS3ST3B1	ITM2A
IGK@	HLA-DOA	HVCN1	JAK3
IGKC	HLA-DOB	ICOS	KCNA3
IGLC1	HLA-DPB1	IDO1	KIAA0125
IGLJ3	HLA-DQA1	IDO2	KIF21B
IGLL3P	HLA-DQB1	IFI16	KLHL6
IGLL5	HLA-DRA	**IFNG**	KLRC1
IGLV1-44	HLA-DRB1	IGFLR1	KLRC3
IGLV6-57	HLA-E	IGH@	KLRC4
IKZF1	HLA-F	IGHA1	KLRC4-KLRK1
IKZF3	HLA-F-AS1	IGHD	KLRD1
IL10RA	HMHA1	IGHG1	KLRG1
IL12B	HS3ST3B1	IGHM	LAMP3
IL12RB1	ICOS	IGK@	LAP3
IL16	IDO1	IGKC	LAT
IL18RAP	IDO2	IGLC1	LAX1
IL21R	IFI16	IGLL5	LCK
IL23A	IFNAR2	IKZF1	LCP1
IL26	**IFNG**	IKZF3	LCP2
IL2RA	IGFLR1	IL10RA	LIMD2
IL2RB	IGH@	IL12RB1	LOC100287723
IL2RG	IGHA1	IL16	LOC100506776
IL7R	IGHD	IL18RAP	LOC100507463
INPP4A	IGHG1	IL21R	LOC100653340
INPP5D	IGHM	IL23A	LOC285972
IPCEF1	IGHV3-48	IL26	LOC642838
IRF1	IGK@	IL2RB	LRRC18
IRF4	IGKC	IL2RG	LTB
IRF8	IGLL5	IL36B	LY9
ITGA4	IKZF1	IL7R	MAP4K1
ITGAL	IKZF3	INPP5D	MBNL1
ITGB7	IL10RA	IPCEF1	MCOLN2
ITK	IL12B	IRF1	MGC40069

JAK3	IL12RB1	IRF4	MGC42157
JAKMIP1	IL16	IRF8	MIR155
KCNA3	IL18RAP	ITGA2B	MIR3929
KCNJ10	IL21R	ITGA4	MPEG1
KIAA0125	IL23A	ITGAL	MS4A1
KIF21B	IL2RB	ITGAX	MYO1G
KIF2A	IL2RG	ITGB7	NCKAP1L
KLHL6	IL7R	ITK	NEDD9
KLRB1	INPP5D	ITM2A	NFATC2
KLRC3	IPCEF1	JAK3	NKG7
KLRC4	IRF1	KCNA3	NLRC5
KLRC4-KLRK1	IRF4	KIAA0125	P2RX5
KLRD1	IRF8	KIAA1191	P2RY10
KLRG1	ITGA4	KIF21B	P2RY8
LAMP3	ITGAL	KIF2A	PAG1
LAP3	ITGB7	KIR3DL1	PARP15
LAT	ITK	KLHL6	PARP8
LAX1	JAK3	KLRB1	PARVG
LCK	KBTBD8	KLRC4-KLRK1	PATL2
LCP1	KCNA3	KLRD1	PAX5
LCP2	KCNJ10	KLRG1	PIK3CD
LGALS2	KIAA0125	LAMP3	PIK3CG
LILRB2	KIF21B	LAT	PIK3R5
LIMD2	KLHL6	LAX1	PIM2
LOC100130100	KLRC3	LCK	PLA2G2D
LOC100287723	KLRC4	LCP1	PLAC8
LOC100505658	KLRC4-KLRK1	LCP2	PLCXD2
LOC100505746	KLRD1	LGALS2	POU2AF1
LOC100506776	LAMP3	LINC00184	POU2F2
LOC100507463	LAP3	LOC100128843	PPP1R16B
LOC100508797	LAPTM5	LOC100287723	PPP2R5C
LOC100653340	LAT	LOC100505746	PRF1
LOC285628	LAT2	LOC100505812	PRKCB
LOC285972	LAX1	LOC100506776	PRKCH
LOC439949	LCK	LOC100507164	PRKCQ
LOC642838	LCP1	LOC100507206	PSTPIP1
LOC96610	LCP2	LOC100507463	PTCSC1
LRRC18	LGALS2	LOC100653340	PTN
LST1	LILRA6	LOC285628	PTPN22
LTB	LILRB1	LOC285972	PTPN7
LY9	LILRB2	LOC401093	PTPRC

LYZ	LOC100129518	LOC439949	PVRIG
MAL	LOC100505501	LOC643733	PYHIN1
MAP4K1	LOC100505812	LOC79015	RAB33A
MBNL1	LOC100506776	LOC96610	RAC2
MCOLN2	LOC100507286	LPXN	RASA2
MEI1	LOC100507463	LRCH4	RASSF5
MGC40069	LOC100507616	LRMP	RNASE6
MGC42157	LOC100653340	LRRC18	RTKN2
MIAT	LOC285628	LST1	RUNX3
MIR155	LOC285972	LTA	SAMD3
MIR3929	LOC401093	LTB	SAMSN1
MS4A1	LOC439949	LY9	SASH3
MYO1F	LPXN	MAL	SCML4
MYO1G	LRMP	MAP4K1	SEL1L3
MZB1	LRRC18	MARC1	SELL
NABP1	LST1	MBNL1	SEMA4D
NAPSB	LTA	MCOLN2	SEPT1
NCF1C	LTB	MEI1	SEPT6
NCLN	LY9	MGC12916	SH2D1A
NDUFA2	LYN	MGC40069	SH2D2A
NEDD9	LYZ	MGC42157	SIRPG
NFATC2	MAP4K1	MIAT	SLA
NKG7	MARC1	MIR155	SLA2
NLRC5	MCOLN2	MIR3929	SLAMF1
NOL3	MEI1	MOG	SLAMF6
P2RX5	MGC12916	MPEG1	SLAMF7
P2RY10	MGC40069	MS4A1	SLC25A53
P2RY8	MGC42157	MYCBP2	SMCHD1
PAG1	MIAT	MYO1G	SNX20
PARP15	MIR155	NABP1	SP140
PARP8	MIR3929	NCKAP1L	SPOCK2
PARVG	MLKL	NCR1	SRGN
PATL2	MNDA	NEDD9	ST8SIA1
PAX5	MPEG1	NEO1	STAMBPL1
PDE7A	MS4A1	NFATC2	STAP1
PIK3CD	MS4A6A	NIN	STAT4
PIK3CG	MYO1G	NKG7	STK17B
PIK3R5	NABP1	NLRC5	STK4
PIM2	NCKAP1L	NLRP7	TAGAP
PLA2G2D	NCLN	NUB1	TARP
PLAC8	NEDD9	OXNAD1	TBC1D10C

PLCXD2	NFATC2	P2RX5	TBX21
PLEK	NKG7	P2RY10	TESPA1
PNOC	NLRC5	P2RY8	THEMIS
POU2AF1	OASL	PAG1	TIGIT
POU2F2	P2RX5	PARP15	TLR10
PPP1R16B	P2RY10	PARVG	TMC8
PPP2R5C	P2RY8	PATL2	TNFAIP3
PRF1	PAG1	PBRM1	TNFAIP8
PRKCB	PARP14	PCBP1	TNFRSF17
PRKCQ	PARP15	PCED1B	TNFSF8
PRO0471	PARP8	PDE3B	TOX
PSTPIP1	PARVG	PIK3CD	TRABD2A
PTCSC1	PATL2	PIK3CG	TRAC
PTK2B	PDCD1LG2	PIK3R5	TRAF1
PTPN22	PIK3AP1	PIM2	TRAF3IP3
PTPN7	PIK3CD	PLA2G2D	TRAT1
PTPRC	PIK3CG	PLAC8	TRAV8-3
PTPRCAP	PIK3R5	PLCG2	TRBC1
PVRIG	PIM2	PLCXD2	TXK
PYHIN1	PLA2G2D	PLEK	UBASH3A
RAB33A	PLAC8	PML	VAV1
RAC2	PLCG2	POU2AF1	WIPF1
RASAL3	PLEK	POU2F2	**XCL1**
RASGRP1	PNOC	PPP1R16B	YME1L1
RASSF5	POU2AF1	PRF1	ZAP70
RCSD1	POU2F2	PRKCB	ZBED2
RGL4	PPP1R16B	PRKCQ	ZBP1
RHOF	PPP2R5C	PSTPIP1	ZC3H12D
RHOH	PRF1	PTCSC1	ZNF831
RUNX3	PRKCB	PTGER4	
SAMD3	PRKCQ	PTPN22	
SAMD9L	PRO1768	PTPN6	
SAMSN1	PSMB9	PTPN7	
SASH3	PSTPIP1	PTPRC	
SEL1L3	PTCSC1	PTPRCAP	
SELL	PTPN22	PVRIG	
SEMA4D	PTPN7	PYHIN1	
SEPT1	PTPRC	PZP	
SEPT6	PVRIG	RAB37	
SH2D1A	PYHIN1	RAC2	
SH2D2A	RAC2	RASAL3	

SIRPG	RASSF5	RASGRP2	
SIT1	RCSD1	RASSF5	
SLA	RUNX3	RCSD1	
SLA2	SAMD3	RGL4	
SLAMF1	SAMD9L	RNF166	
SLAMF6	SAMHD1	RPS15	
SLAMF7	SAMSN1	RUNX3	
SLC15A2	SASH3	SAMD3	
SLC25A53	SEL1L3	SAMD9L	
SMCHD1	SELL	SAMHD1	
SNX20	SELPLG	SAMSN1	
SPIB	SEMA4D	SASH3	
SPOCK2	SEPT6	SCML4	
STAMBPL1	SERPINB9	SEL1L3	
STAP1	SH2D1A	SELL	
STAT4	SH2D2A	SELP	
STK17A	SIRPG	SELPLG	
STK17B	SLA	SEMA4D	
STK4	SLA2	SEPT1	
SYNRG	SLAMF1	SEPT6	
TAGAP	SLAMF6	SH2D1A	
TAP1	SLAMF7	SH2D2A	
TAP2	SLAMF8	SH2D3C	
TARP	SLC15A2	SIRPG	
TBC1D10C	SMAP2	SIT1	
TBX21	SMCHD1	SLA	
TESPA1	SNX20	SLA2	
THEMIS	SP110	SLAMF1	
TIFAB	SP140L	SLAMF6	
TIGIT	SPIB	SLAMF7	
TLR10	SPOCK2	SLC25A53	
TLR8	SRGN	SMTNL1	
TMC8	ST8SIA4	SNX20	
TNF	STAP1	SOCS1	
TNFAIP3	STAT1	SOX15	
TNFAIP8	STAT4	SP110	
TNFAIP8L2	STK4	SP140	
TNFRSF17	SYK	SPIB	
**TNFSF13B**	TAGAP	SPOCK2	
TNFSF8	TAP1	SRGN	
TOX	TAP2	ST8SIA4	

TRAC	TARP	STAMBPL1	
TRAF1	TBC1D10C	STAP1	
TRAF3IP3	TBX21	STAT4	
TRAT1	TESPA1	STK17A	
TRAV8-3	TFEC	STK17B	
TRBC1	THEMIS	STK4	
TXK	TIFAB	TAGAP	
UBASH3A	TIGIT	TAP1	
WDFY4	TLR10	TARP	
WIPF1	TLR8	TBC1D10C	
**XCL1**	TMC8	TBX21	
YME1L1	TMEM156	TCL1A	
ZAP70	TNF	TESPA1	
ZBED2	TNFAIP3	TEX35	
ZBP1	TNFAIP8	TFEC	
	TNFAIP8L2	THEMIS	
	TNFRSF17	TIFAB	
	TNFRSF1B	TIGIT	
	**TNFSF13B**	TLE2	
	TNFSF8	TLE3	
	TNIP3	TLR10	
	TOX	TMC8	
	TRAC	TMEM156	
	TRAF3IP3	TNFAIP3	
	TRAT1	TNFAIP8	
	TRAV8-3	TNFRSF13B	
	TRBC1	TNFRSF17	
	TRIM22	TNFRSF25	
	TRIM69	**TNFSF13B**	
	TSPAN3	TNFSF14	
	TXK	TNIP3	
	UBASH3A	TOX	
	USP15	TRABD2A	
	VAV1	TRAC	
	VCAM1	TRAF1	
	VNN2	TRAF3IP3	
	WARS	TRAT1	
	WAS	TRAV8-3	
	WDFY4	TRBC1	
	WIPF1	TRDV3	
	XAF1	TREML2	

	**XCL1**	TSPAN32	
	YME1L1	TTN	
	ZAP70	TXK	
	ZBED2	UBASH3A	
	ZBP1	VAV1	
	ZC3H12D	VNN2	
		WAS	
		WDFY4	
		WIPF1	
		**XCL1**	
		YME1L1	
		ZAP70	
		ZBED2	
		ZBP1	
		ZC3H12D	
		ZHX2	
		ZNF101	
		ZNF683	
		ZNF831	

Supplementary Table S1: Regulons of the transcription factors inferred via the ARACNE algorithm in the NACT dataset. (Table S1 is a separet file).

Master Regulator	FET P-Value	Markers in regulon	Markers in intersection set	Mode
**IKZF3**	4.60E-152	375	255	-
**SCML4**	4.49E-147	419	266	-
**SP140**	9.48E-145	407	260	-
**ZNF831**	1.74E-138	308	220	-
**BNC2**	7.24E-22	369	108	+
**HOXA5**	4.37E-20	471	124	+
**PRRX1**	2.42E-18	341	96	+
**SNAI2**	1.23E-12	90	36	+
**TBX5**	8.01E-10	222	57	+
**TWIST1**	3.34E-09	162	45	+
**ID4**	9.06E-09	248	60	+
**TEAD1**	7.69E-08	86	30	+

Supplementary Table S2: bCSC Master Regulators – Genes selected as master regulators in the "bCSC/CC" dataset by MRA-FET. Mode "-" = negative correlated with the bCSC phenotype, "+" = positive correlated with the bCSC phenotype.

	pCR	nCR	Marginal Row Totals
Metagene Score High	6 (13.62) [4.26]	52 (44.38) [1.31]	58
Metagene Score Low	21 (13.38) [4.34]	36 (43.62) [1.33]	57
Marginal Column Totals	27	88	115 (Grand Total)

Supplementary Table S3: Pathological Response Group, Chi2 Analysis. When ordered by the rank of the metagene of all TFs in the two validated networks and divided into two groups there is a ratio of 3.57 of pCR in the group expressing less cancer stem cell TFs. p= 0.0008. The Chi-square statistic is 11.2346. pCR = pathological complete response to paclitaxel, nCR = No pathological complete response to paclitaxel.

Protein	Caspase.7_cleavedD198		Lck		Syk	
Subtype	r	p	r	p	r	p
LumA	0.42	< 0.00001	0.58	< 0.00001	0.28	0.0009
LumB	0.47	< 0.00001	0.56	< 0.00001	0.46	< 0.00001
HER2	0.46	< 0.00001	0.48	< 0.00001	0.45	< 0.00001
Basal	0.54	< 0.00001	0.63	< 0.00001	0.35	< 0.00001
Total	0.51	< 0.00001	0.51	< 0.00001	0.41	< 0.00001

Suplementary Table S4: Pearson Correlation between the expression of the Immune Metagene and the level of Cleaved Caspase 7, Lck and Syk in the PAM50 subtype of the TCGA-BRCA dataset.

SYMBOL		
ARHGAP9	GNLY	NKG7
BCL11B	GZMA	P2RX5
BTLA	GZMK	P2RY10
CCL5	ICOS	P2RY8
CCR2	IFNG	PLAC8
CD2	IGHM	POU2AF1
CD247	IGKC	PPP1R16B
CD28	IL18RAP	PRF1
CD3D	IL21R	PTPRC
CD48	IL2RB	PVRIG
CD52	IL2RG	RUNX3
CD69	IL7R	SAMD3
CD8A	INPP5D	SELL
CD96	ITK	SPOCK2
CORO1A	KLRD1	STAT4
CRTAM	LCK	TBC1D10C
CTLA4	LTB	TRAC
CXCR6	LY9	TRAT1
EOMES	MAP4K1	XCL1
FCRL3	MS4A1	ZBED2

Supplementary Table S5: 60 genes from the "Immune response transcription module" with the greater up-regulation in the bCSC/CC dataset. $p<0.05$.

Patient Number	Age	Histologic Grade	ER	PgR	HER2	T	N	M	Clinical Stage
**12**	50	3	POS	POS	POS	4B	3C	1	IV
**14**	61	3	NEG	NEG	POS	4D	2	1	IV
**20**	40	3	NEG	NEG	NEG	2	0	0	IIA

Supplementary Table S6: bCSC/Bulk dataset Clinical Data. Clinical data from the patients whose samples fulfilled all the requirements of the study. These patients were included in the "bCSC/Bulk" dataset. ER: Estrogen receptors, PgR: Progesterone receptors, HER2: HER2 receptors. TNM staging as used by the American Joint Committee on Cancer. T.N.M – AJCC classification, T = Tumor stage (The tumour being 2cm across or less = 1, The tumor being larger than 5cm and Inflammatory = 4D), N = Lymphonod stage (No cancer cells found in any nearby nodes = 0, cancer cells in lymph nodes above the collarbone = 3c), M = Metastasis stage (No signals of mestastasis = 0, Metastasis = 1). Clinical stage: Anatomic stage/prognostic groups based on the T.N.M evaluation. Histological Grade: Nottingham–Bloom–Richardson system, Grade 1 = well differentiated, Grade 3 = poorly differentiated.

Replicates	ER	PR	HER2
REP 1	+	+	-
REP 2	-	-	-
REP 3	-	-	+
REP 4	+	+	-
REP 5	ND	ND	ND
REP 6	+	+	-
REP 7	+	+	-
REP 8	+	+	-

Supplementary Table S7: Immunohistochemical status from the samples in "bCSC/CC " dataset (GSE52327). Breast Cancer Stem Cell X Cancer Cell, ER = Estrogen Receptor, PR = Progesterone Receptor, HER2 = human epidermal growth factor receptor 2, POS = Positive, NEG = Negative. + = positive, - = negative, ND = information Not Available.

Status	pCR	nCR	Total
ER+	5	50	55
HER2+	12	22	34
TN	10	16	26
Total	27	88	115

Supplementary Table S8: Clinical Data from the "Clinical Response" dataset (GSE32646) tissue samples. Status = Expression of Immunohistochemical Markers, ER+ = Estrogen Receptor positive, HER2+ = human epidermal growth factor receptor 2 positive, TN = Triple Negative, negative for the expression of ER, HER2 and Progesterone Receptor; pCR= Pathological Complete Response to paclitaxel, nCR = Non-Complete Response to paclitaxel**.**

NAME	NOM p-val	FDR q-val
SNAI2_REGULON	< 0.001	0.001
TWIST1_REGULON	< 0.001	0.005
TBX5_REGULON	< 0.001	4.94E-04
BNC2_REGULON	< 0.001	6.67E-04
PRRX1_REGULON	< 0.001	5.56E-04

Supplementary Table S9: **GSEA analysis, Xenografts – Grown vs Engrafted. Positive correlation – regulons of the mesenchymal module transcription factors.** Enrichment results of the positive correlation to Grown samples of GSEA analysis using the mesenchymal module transcription factor regulons in the xenograft dataset.

NAME	NOM p-val	FDR q-val
ZNF831_REGULON	< 0.001	< 0.001
SCML4_REGULON	< 0.001	< 0.001
SP140_REGULON	< 0.001	< 0.001
IKZF3_REGULON	< 0.001	< 0.001

Supplementary Table S10: **GSEA analysis, Xenografts – Grown vs Engrafted. Negative correlation – regulons of the mesenchymal module transcription factors.** Enrichment results of the negtive correlation to Grown samples of GSEA analysis using the mesenchymal module transcription factor regulons in the xenograft dataset.

NAME	NOM p-val	FDR q-val
LIM_MAMMARY_STEM_CELL_UP	< 0.001	< 0.001
HALLMARK_EPITHELIAL_MESENCHYMAL_TRANSITION	< 0.001	< 0.001
BOQUEST_STEM_CELL_UP	< 0.001	< 0.001

Supplementary Table S11: **GSEA analysis, Xenografts – Grown vs Engrafted. Positive correlation – Mesenchymal and Stem Cells genesets.**

NAME	NOM p-val	FDR q-val
KEGG_GRAFT_VERSUS_HOST_DISEASE	< 0.001	< 0.001
KEGG_T_CELL_RECEPTOR_SIGNALING_PATHWAY	< 0.001	< 0.001
KEGG_NATURAL_KILLER_CELL_MEDIATED_CYTOTOXICITY	< 0.001	< 0.001
KEGG_ANTIGEN_PROCESSING_AND_PRESENTATION	< 0.001	< 0.001

Supplementary Table S12: **GSEA analysis, Xenografts – Grown vs Engrafted. Negative correlation – KEGG Imunne genesets.**

Top 20	NAME	NOM p-val	FDR q-val
1	GO_ADAPTIVE_IMMUNE_RESPONSE	< 0.001	< 0.001
2	GO_ACTIVATION_OF_IMMUNE_RESPONSE	< 0.001	< 0.001
3	GO_POSITIVE_REGULATION_OF_IMMUNE_RESPONSE	< 0.001	< 0.001
4	GO_LYMPHOCYTE_ACTIVATION	< 0.001	< 0.001
5	GO_POSITIVE_REGULATION_OF_CELL_ACTIVATION	< 0.001	< 0.001
6	GO_LEUKOCYTE_ACTIVATION	< 0.001	< 0.001
7	GO_ANTIGEN_RECEPTOR_MEDIATED_SIGNALING_PATHWAY	< 0.001	< 0.001
8	GO_REGULATION_OF_CELL_ACTIVATION	< 0.001	< 0.001
9	GO_REGULATION_OF_LEUKOCYTE_PROLIFERATION	< 0.001	< 0.001
10	GO_POSITIVE_REGULATION_OF_INTERFERON_GAMMA_PRODUCTION	< 0.001	< 0.001
11	GO_POSITIVE_REGULATION_OF_INNATE_IMMUNE_RESPONSE	< 0.001	< 0.001
12	GO_CELLULAR_RESPONSE_TO_INTERFERON_GAMMA	< 0.001	< 0.001
13	GO_REGULATION_OF_HOMOTYPIC_CELL_CELL_ADHESION	< 0.001	< 0.001
14	GO_LEUKOCYTE_CELL_CELL_ADHESION	< 0.001	< 0.001
15	GO_ADAPTIVE_IMMUNE_RESPONSE_BASED_ON_SOMATIC_RECOMBINATION_OF_IMMUNE_RECEPTORS_BUILT_FROM_IMMUNOGLOBULIN_SUPERFAMILY_DOMAINS	< 0.001	< 0.001
16	GO_REGULATION_OF_INTERFERON_GAMMA_PRODUCTION	< 0.001	< 0.001
17	GO_RESPONSE_TO_INTERFERON_GAMMA	< 0.001	< 0.001
18	GO_T_CELL_DIFFERENTIATION	< 0.001	< 0.001
19	GO_IMMUNE_EFFECTOR_PROCESS	< 0.001	< 0.001
20	GO_IMMUNE_RESPONSE_REGULATING_CELL_SURFACE_RECEPTOR_SIGNALING_PATHWAY	< 0.001	< 0.001

Supplementary Table S13: **Top 20 G.O terms. GSEA analysis, Xenografts – Grown vs Engrafted. Negative correlation.**

Supplementary Technical References

1 Thomson-Reuters. *KEY PATHWAY ADVISOR (KPA) - POWERED BY THOMSON REUTERS METACORE™*, <http://images.info.science.thomsonreuters.biz/Web/ThomsonReutersScience/%7B6ab21059-7923-454a-9e86-f14b482d242f%7D_S021144_KPAFactsheet_Final.pdf> (2017).

2 Shannon, P. *et al.* Cytoscape: a software environment for integrated models of biomolecular interaction networks. *Genome Res* **13**, 2498-2504, doi:10.1101/gr.1239303 (2003).

3 Ma, C. X. & Ellis, M. J. The Cancer Genome Atlas: clinical applications for breast cancer. *Oncology (Williston Park)* **27**, 1263-1269, 1274-1269 (2013).

4 Zhu, Y., Qiu, P. & Ji, Y. TCGA-assembler: open-source software for retrieving and processing TCGA data. *Nat Methods* **11**, 599-600, doi:10.1038/nmeth.2956 (2014).

5 Haibe-Kains, B. *et al.* A three-gene model to robustly identify breast cancer molecular subtypes. *J Natl Cancer Inst* **104**, 311-325, doi:10.1093/jnci/djr545 (2012).

6 Li, J. *et al.* TCPA: a resource for cancer functional proteomics data. *Nat Methods* **10**, 1046-1047, doi:10.1038/nmeth.2650 (2013).

7 Yoshihara, K. *et al.* Inferring tumour purity and stromal and immune cell admixture from expression data. *Nat Commun* **4**, 2612, doi:10.1038/ncomms3612 (2013).

8 Gautier, L., Cope, L., Bolstad, B. M. & Irizarry, R. A. affy--analysis of Affymetrix GeneChip data at the probe level. *Bioinformatics* **20**, 307-315, doi:10.1093/bioinformatics/btg405 (2004).

9 Carvalho, B. S. & Irizarry, R. A. A framework for oligonucleotide microarray preprocessing. *Bioinformatics* **26**, 2363-2367, doi:10.1093/bioinformatics/btq431 (2010).

10 Morrissey, E. R. & Diaz-Uriarte, R. Pomelo II: finding differentially expressed genes. *Nucleic Acids Res* **37**, W581-586, doi:10.1093/nar/gkp366 (2009).

11 Pawitan, Y., Michiels, S., Koscielny, S., Gusnanto, A. & Ploner, A. False discovery rate, sensitivity and sample size for microarray studies. *Bioinformatics* **21**, 3017-3024, doi:10.1093/bioinformatics/bti448 (2005).

12 Warnes, M. G. R., Bolker, B. & Bonebakker, L. Package ‘gplots’. *Various R Programming Tools for Plotting Data* (2016).

13 Murtagh, F. & Contreras, P. Algorithms for hierarchical clustering: an overview. *Wiley Interdisciplinary Reviews: Data Mining and Knowledge Discovery* **2**, 86-97 (2012).
